# EDA Complex–Mediated C(sp^3^)–H Cross-Dehydrogenative Coupling Enables Synthesis of Noncanonical *α*,*β*-Diamino Acids

**DOI:** 10.1002/ejoc.70356

**Published:** 2026-03-06

**Authors:** Krishnakumar Sachidanandan, Cole Stenftenagel, Athul Joshy, Alexander M. Cluff, Sébastien Laulhé

**Affiliations:** Department of Chemistry & Chemical Biology, Indiana University Indianapolis, Indianapolis, Indiana, USA

**Keywords:** *α*,*β*-diamino acid, aryl radical, cross-dehydrogenative coupling, electron donor–acceptor complex

## Abstract

Nonproteinogenic *α*,*β*-diamino acids are key motifs in bioactive compounds. We report a photoinduced cross-dehydrogenative coupling (CDC) of *N*-arylglycine derivatives with amides. This reaction proceeds via an electron donor–acceptor (EDA) complex that enables regioselective C(sp^3^)–C(sp^3^) cross-coupling, providing access to non-canonical *α*,*β*-diamino acids from simple precursors.

## 1 | Introduction

**SCHEME 1 |:**
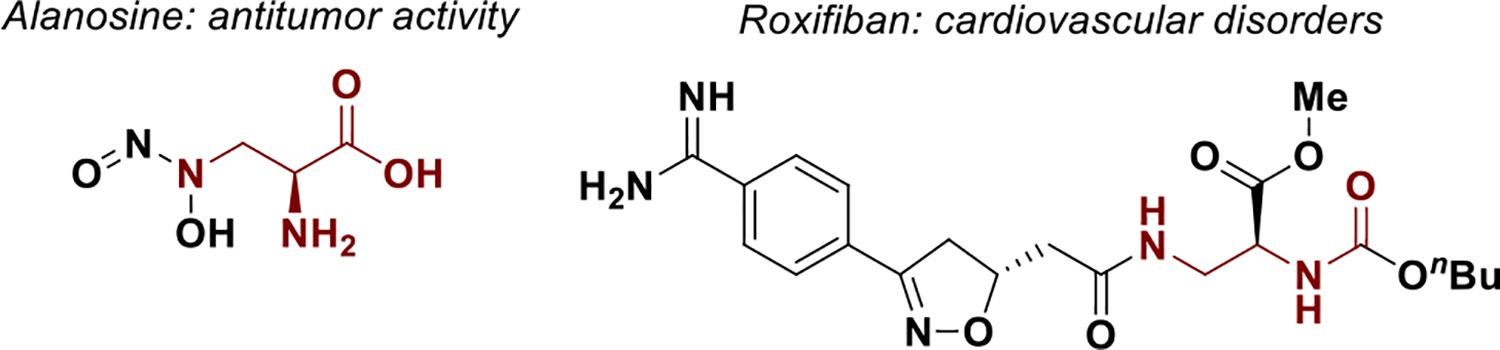
Drugs motifs containing *α*,*β*-diamino acid derivatives.

Noncanonical amino acids (nCAAs) have emerged as pivotal structural motifs in drug discovery, due to their enhanced versatility compared to their natural counterparts [1–3]. This has accelerated their integration into medicinal chemistry libraries for the development of novel therapeutics [4–8]. In particular, *α*,*β*-diamino acid containing small molecules have garnered sustained attention due to their diverse pharmacological profiles [9, 10]. Notable examples include alanosine, which exhibits antitumor activity [11, 12], and roxifiban, a synthetic agent developed for cardiovascular disorders (Scheme 1) [13–16]. Furthermore, *α*,*β*-diamino acids serve as valuable precursors for the synthesis of 1,2-diamines, which find broad application as chiral auxiliaries and as ligands in asymmetric metal catalysis [17].

**SCHEME 2 |:**
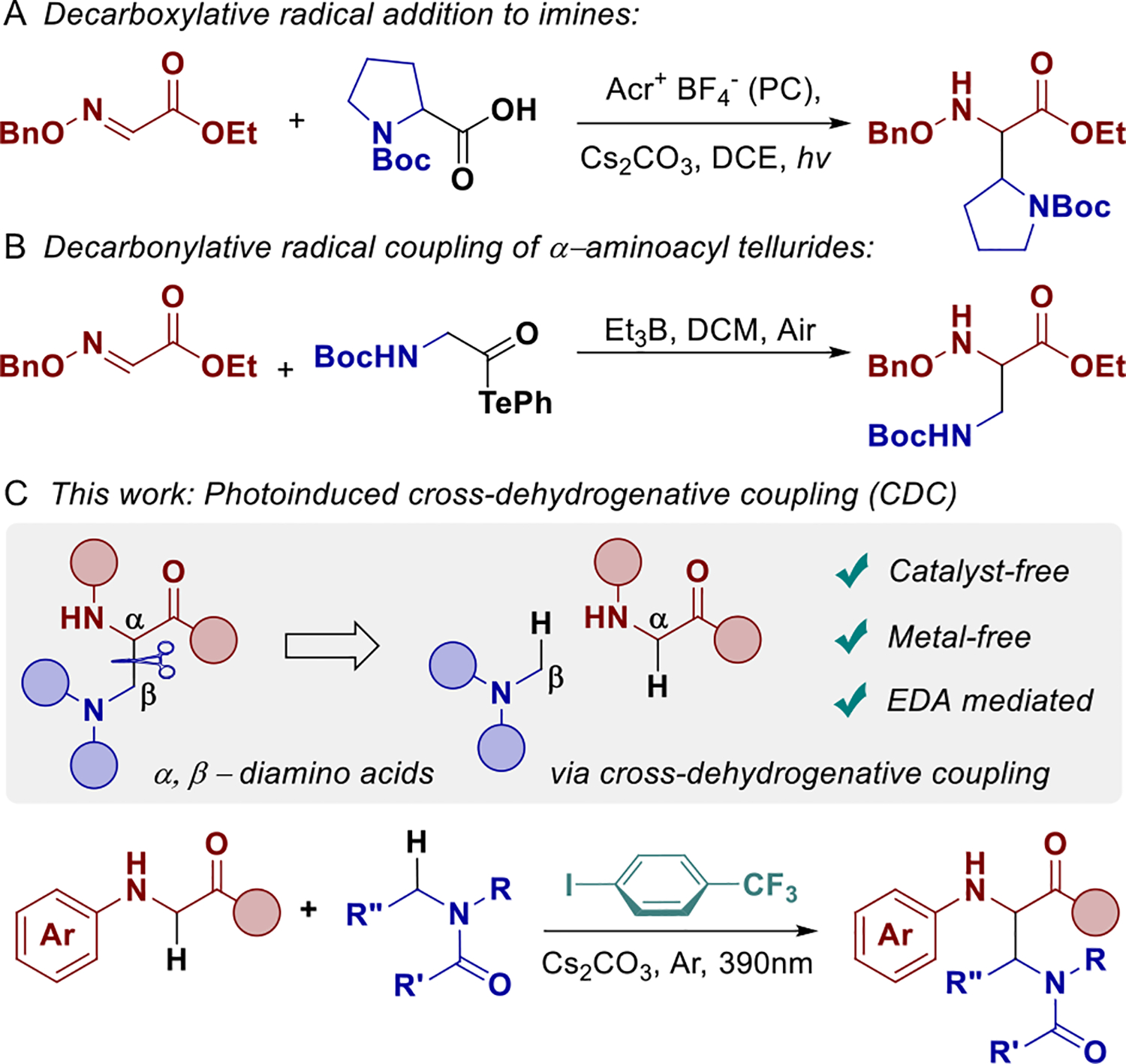
(A) Previous decarboxylative reaction with oximes. (B) Previous α-aminoacyl tellurides reaction with oximes. (C) Our cross-dehydrogenative coupling approach.

In recent years, there has been increased interest in accessing *α*,*β*-diamino acid scaffolds using imines and oximes as reactive coupling partners [18]. Among these methods, only two radical-based transformations have been developed, and they require the use of oxime derivatives [19, 20]. These examples proceed via (i) photo-catalytic decarboxylation (Scheme 2A) [19] and (ii) borane-mediated decarbonylation (Scheme 2B) [20].

Unfortunately, these strategies suffer from limited versatility given that they are inherently restricted to noncommercially available oxime-based systems, reliant on pre-functionalized radical precursors, and in one case they require highly flammable Et_3_B (auto ignites at −20°C in air). In contrast, a more straightforward approach would involve a cross-dehydrogenative coupling strategy [21], whereby two C(sp^3^)–H bonds are simultaneously activated enabling direct bond formation between both species. This eliminates the need for prefunctionalized auxiliaries and mitigates associated synthetic drawbacks such as accessing the oxime intermediates.

Electron donor–acceptor (EDA) complexes have prompted considerable attention over the past decade, unlocking a broad spectrum of molecular transformations that were once confined to metal-catalyzed or otherwise intricate systems [22–26]. Our group has actively contributed to this growing toolbox, developing versatile methodologies that emphasize the operational simplicity and environmentally benign nature of aryl halide-based EDA strategies [27–29]. Electron-deficient aryl halides proved to be particularly versatile as hydrogen-atom transfer (HAT) agents in cross-dehydrogenative processes as we demonstrated previously in our work [27], and elegantly re-emphasized recently by Gevorgyan and Wang [30–32]. Building on these advances, we report a photoinduced synthesis of noncanonical *α*,*β*-diamino acids via EDA complex–mediated cross-dehydrogenative coupling (Scheme 2C).

## 2 | Results and Discussion

Optimization studies commenced using *N*-phenyl-2-(phenylamino)acetamide (**1**) as the amino acid derivative and *N*,*N*-dimethylformamide (**2**) as the alkane coupling partner to furnish the target product, 3-(*N*-methylformamido)-*N*-phenyl-2(phenylamino)propanamide (**3a**) (Table 1). Under optimal conditions, employing 1-iodo-4-(trifluoromethyl)benzene (**Ar-1**) and Cs_2_CO_3_, afforded product **3a** in a commendable 52% yield (Entry 1).

However, altering the solvent system from neat DMF to a 1:1 mixture of DMF and MeCN resulted in a significant drop of yield to 19% (Entry 2). Modifying the electronics of the aryl halide to electron-rich or electronically neutral variants proved detrimental to the reaction efficiency (Entries 3 and 4), presumably due to a less efficient EDA complex formation between the aryl iodide and the glycine derivative. Substituting Cs_2_CO_3_ with either K_2_CO_3_ or Et_3_N led to a marked decrease in product yield, affording **3a** in only 14% and 13%, respectively (Entries 5 and 6). Altering the irradiation wavelength to lower-energy 440 nm also proved detrimental, resulting in a reduced yield of 22% (Entry 7). Furthermore, control experiments in the absence of light yielded no detectable product (Entry 8) (see Supporting Information for detailed data, page SI-5).

**SCHEME 3 |:**
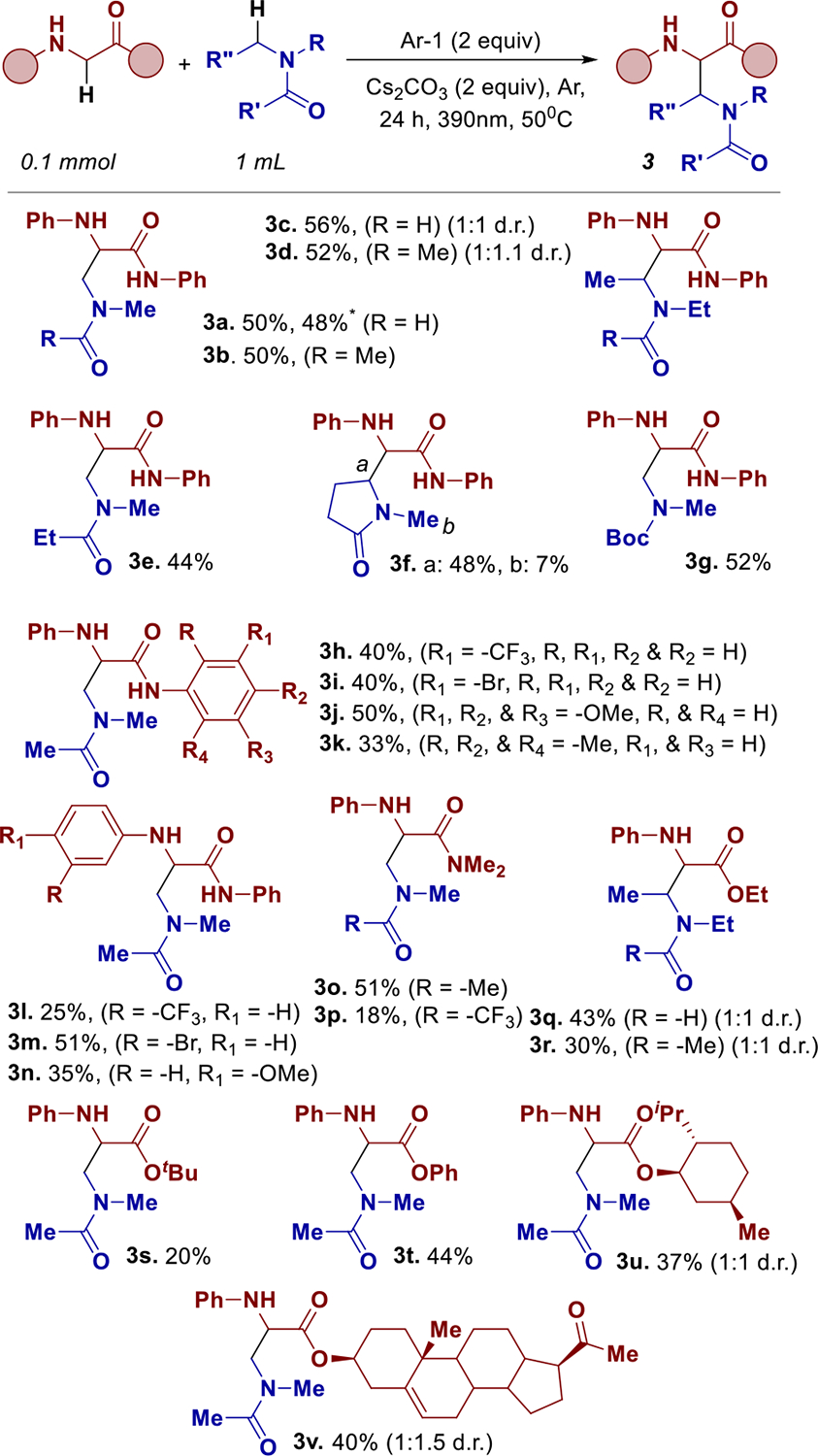
Substrate scope. * 1.0 mmol.

Next, we turned our attention to exploring the substrate scope of this transformation, beginning with variations in the alkyl motifs while retaining compound **1** as the glycine-derived scaffold (Scheme 3). Both dimethylformamide and dimethylacetamide were successfully coupled under the optimized conditions, affording the desired *α*,*β*-diamino acid derivatives **3a** and **3b** in 50% yield each. Similarly, the formamide and acetamide analogs of the *N*,*N*-dimethyl system were well tolerated, delivering products **3c** and **3d** in 56% and 52% yields, respectively. *N*,*N*-dimethylpropionamide was successfully coupled with substrate **1**, affording the corresponding *α*,*β*-diamino acid **3e** in 44% yield. Notably, the transformation also tolerated a cyclic amide, 1-methylpyrrolidin-2-one, delivering the product **3f** as a mixture of regioisomers in 55% yield, with the major product favoring the activation of the methylene moiety over the methyl group. Importantly, Boc-protected amines were tolerated and afforded product **3g** in synthetically useful 52% yield. This result highlights the method’s compatibility with carbamate functionalities, expanding its utility for further functionalizations.

The scope of the transformation was further explored using various glycine derivatives, wherein *N*,*N*-dimethylacetamide served as the alkylating motif. Initial efforts focused on substrates bearing aromatic substitutions on the amide side chain of the amino acid. Meta-substituted electron withdrawing (-CF_3_) and halogenated (-Br) derivatives were tolerated in forming products **3h** and **3i** both in 40% yields. Electron-rich aromatic substitutions also afforded the desired products. Specifically, the tri-methoxy-phenyl derivative furnished product **3j** in 50% yield, while the trimethylsubstituted analog afforded **3k** in low 33% yield. Aromatic substitutions on the amine side of the glycine scaffold were further investigated using meta-substituted electron-withdrawing groups. The trifluoromethyl derivative yielded product **3l** in 25%, while the brominated analog afforded **3m** in 51% yield. The electron-donating para-methoxy group delivered the desired product **3n** in 35% yield, demonstrating compatibility with deprotection.

A diverse set of protecting groups were evaluated on the amino acid scaffold, beginning with *N*,*N*-dimethylamide derivatives. Both *N*,*N*-dimethylacetamide (DMA) and 2,2,2-trifluoro-*N*,*N*-dimethylacetamide were well tolerated under the reaction conditions, affording products **3o** and **3p** in 51% and 18% yield, respectively. These results indicate that highly electron-deficient amides are not effective coupling partners for this transformation. The ethyl ester derivative of aryl glycine was employed to couple *N*,*N*-diethylformamide and *N*,*N*-diethylacetamide, affording products **3q** (43%) and **3r** (30%). Both *tert*-butyl and phenyl ester derivatives of aryl glycine also furnished the corresponding DMA-coupled products **3s** (20%) and **3t** (44%). Finally, glycine esters bearing bioactive motifs, menthol and cholesterol, were evaluated in the reaction with DMA, delivering cross-coupled products **3u** and **3v** in moderate yields of 37% and 40%, respectively. In comparison to previously reported ethereal couplings [27], yields reported in this substrate scope may be lower due to stronger *α*-C–H bonds [33] in amide systems. Indeed, remaining amino acid starting material is found unreacted in all these reactions.

**SCHEME 4 |:**
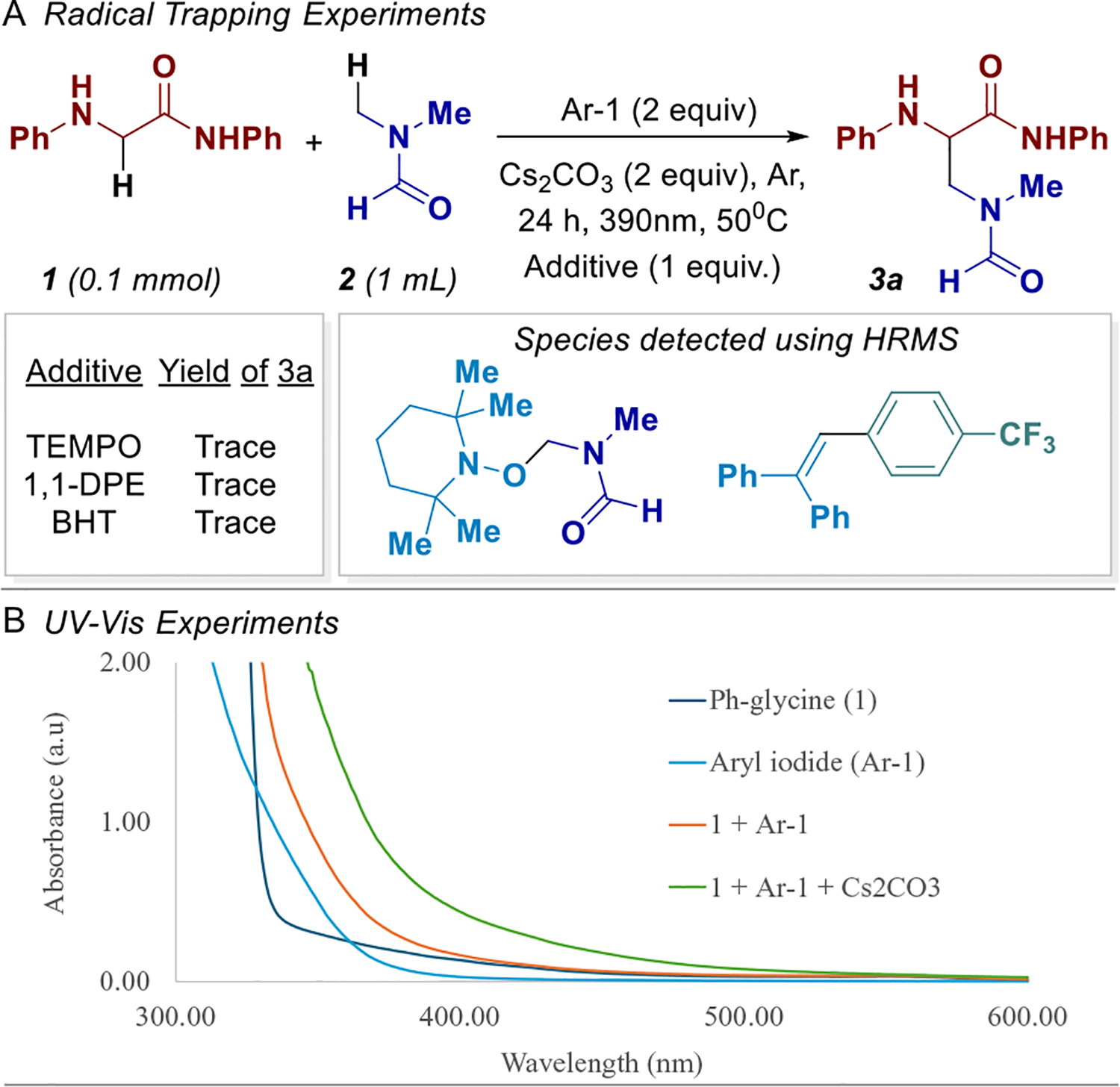
Mechanistic Studies. (A) Radical trapping studies. (B) UV-Vis studies.

To probe the involvement of radical intermediates in the transformation, we conducted a series of radical trapping experiments under standard reaction conditions. The additives, TEMPO, 1,1-diphenylethylene (1,1-DPE), and butylated hydroxytoluene (BHT) were introduced individually in separate reactions (Scheme 4). Formation of the desired product was completely suppressed in all three conditions, suggesting the involvement of a radical-based mechanism. Additionally, both TEMPO and 1,1-DPE effectively trapped multiple radical intermediates, including the DMF- and aryl-derived radicals (see Supporting Information for detailed HRMS and GC–MS data, page SI-30).

To investigate the possibility of photoactive aggregate formation, UV-Vis absorption studies were conducted under various combinations [34, 35]. Notably, the mixture of **1** and **Ar-1** exhibited a pronounced increase in absorption intensity relative to the individual components, suggesting the formation of a ground–state interaction. Upon addition of Cs_2_CO_3_, a further enhancement in absorption was observed, consistent with base-mediated stabilization or deprotonation effects that may facilitate complexation [27]. These observations collectively support the formation of an EDA complex between **1** and **Ar-1**, which likely plays a key role in initiating the photochemical transformation.

Based on experimental results and literature precedent [27, 36, 37], we propose the following mechanism for the transformation. The arylglycine derivative **1** and electron-deficient aryl iodide **Ar-1** form a photoactive EDA complex that undergoes a single electron transfer (SET), generating the amino radical cation **A** and aryl radical anion **B**. Subsequent deprotonation of **A** furnishes the persistent *α*-amino radical **E**. Concurrently, fragmentation of **B** via iodide extrusion yields the aryl radical **C**, which engages in a site-selective HAT process with amide reagent **2** to produce the corresponding *N*-ortho radical **G**.

**SCHEME 5 |:**
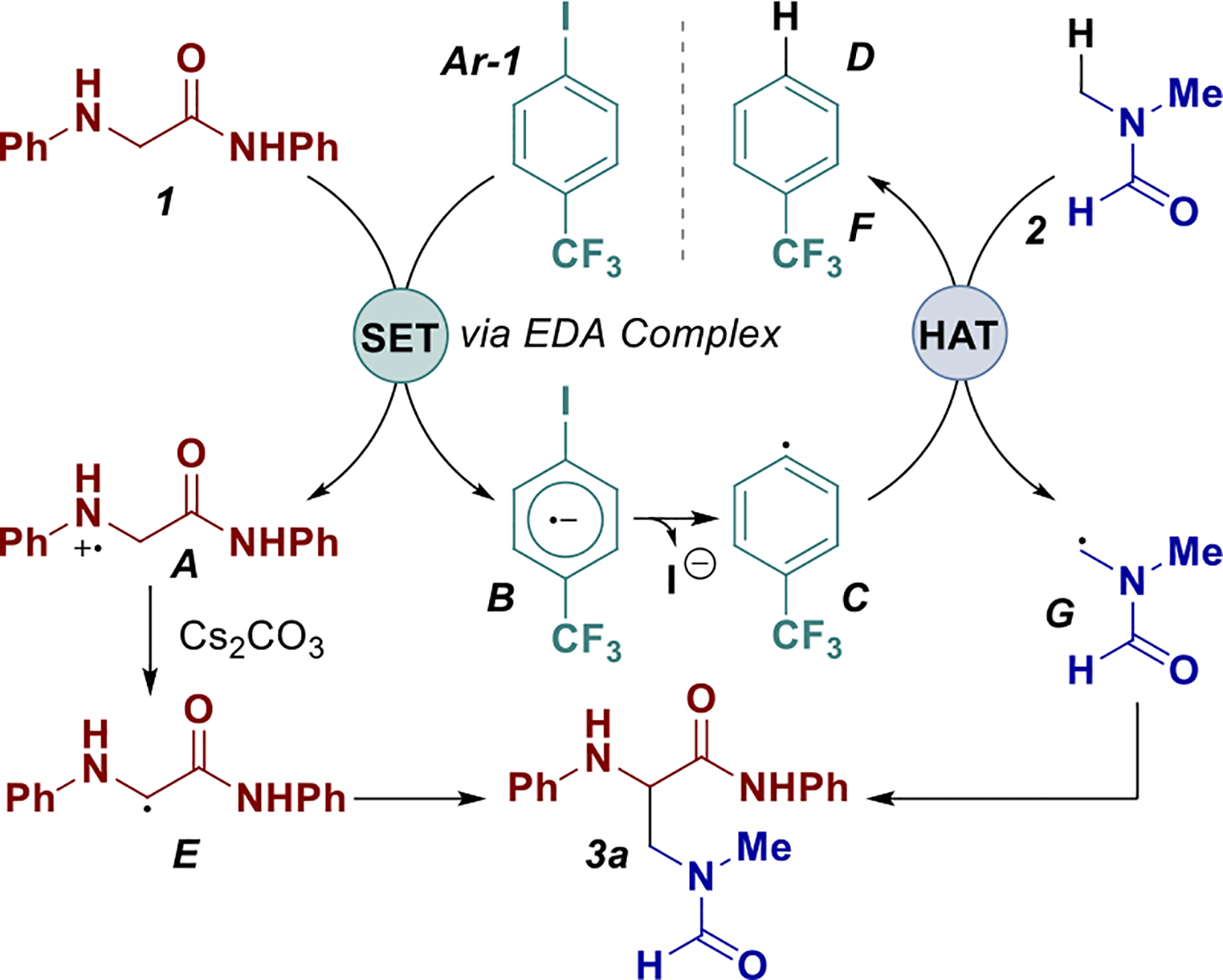
Proposed mechanism.

Finally, radical–radical coupling between **E** and **G** delivers the desired C(sp^3^)–C(sp^3^) cross-coupled product **3a** (Scheme 5).

## 3 | Conclusion

In summary, we have opened a new avenue to access *α*,*β*-diamino acids beyond traditional oxime-based strategies and their limitations. Our EDA-mediated cross-dehydrogenative coupling strategy enables the direct C(sp^3^)–H functionalization of amides and *N*-phenyl glycine derivatives. Ongoing efforts in our laboratory are focused on expanding the scope to include benzylic and heteroatom-containing alkyl motifs, while simultaneously improving overall reaction performance. These investigations aim to broaden the applicability of EDA approaches as robust platforms for C(sp^3^)–C(sp^3^) bond formation.

## Supplementary Material

Supplemental Material

Additional supporting information can be found online in the Supporting Information section. The authors have cited additional references within the Supporting Information [38–40]. **Supporting Table S1:** 1HNMR yields using 1,2-dibromoethane as internal standard.

## Figures and Tables

**TABLE 1 |:** Optimization study.

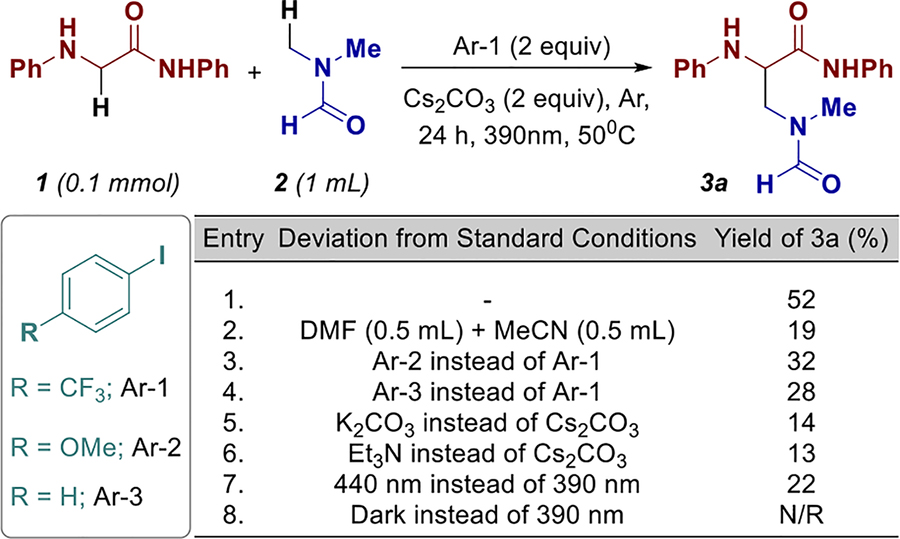

## Data Availability

The data that supports the findings of this study are available in the supplementary material of this article.
