# Supplementary material for: EDA Complex–Mediated C(sp3)–H Cross-Dehydrogenative Coupling Enables Synthesis of Noncanonical α,β-Diamino Acids
Source: European J Org Chem. Author manuscript; Available in PMC 2026 Mar 24. (PMC13008348; doi:10.1002/ejoc.70356)
Supplement: Supplemental Material [file NIHMS2155251-supplement-Supplemental_Material.pdf]

Supporting Information

EDA Complex–Mediated C(sp^3)–H Cross-Dehydrogenative Coupling Enables Synthesis of Non-Canonical α, β-Diamino Acids

Krishnakumar Sachidanandan, Cole Stenftenagel, Athul Joshy, Alexander M Cluff, Sébastien Laulhé*

Indiana University Indianapolis,

Indianapolis, Indiana 46202, United States.

Corresponding Author Email: slaulhe@iu.edu

Table of Contents

1. General Information SI-3
2. General Procedures SI-4
3. Optimization Study SI-5
4. Unsuccessful Substrates SI-6
5. Experimental Details and Characterization Data for Products SI-7
6. Radical Trapping Study SI-30
7. UV-Vis Study SI-32
8. Miscellaneous Studies SI-33
9. ^{1}H NMR, ^{19}F NMR, ^{13}C NMR Spectra SI-34
10. References SI-62

1. General Information

All reagents and solvents were purchased and used without further purification unless otherwise noted. All reactions were performed under an inert atmosphere unless otherwise stated. Room temperature refers to 26 °C, unless otherwise noted. Moisture-sensitive reactions were performed using flame-dried glassware under an atmosphere of dry argon (Ar). Air- and water sensitive reactions were setup in a Vacuum Atmosphere GENESIS glovebox held under an atmosphere of argon gas (working pressure 2−6 mbar). Flame-dried equipment was stored in a 130 °C oven before use and either allowed to cool in a cabinet desiccator or assembled hot and allowed to cool under an inert atmosphere. Chromatographic purification of products were performed manually, using silica flash column chromatography (Fisher Chemical™ Silica Gel Sorbent 230-400 Mesh, Grade 60) or automatically, using Teledyne Isco Combi*Flash RF*+ UV Flash Chromatography System with RediSep Silver flash columns. Thin-layer chromatography was performed on EMD Millipore silica gel 60 F254 glass-backed plates (layer thickness 250 μm, particle size 10−12 μm, impregnated with a fluorescent indicator). Visualization of the developed chromatogram was accomplished by fluorescence quenching under shortwave UV light and/or by staining with phosphomolybdic acid, p-anisaldehyde, or $KMnO_4$ stains.

LED Lamps. The following Kessil LED lamps were used in this work:

- 390 nm lamp: PR160L-390, 40W (purple visible light)
- 427 nm lamp: PR160L-427, 40W (blue visible light)
- 440 nm lamp: PR160L-440, 40W (blue visible light)
- 525 nm lamp: PR160L-525, 40W (green visible light)

Reaction Vials. We used ChemGlass microwave reaction vials with heavy walls made of borosilicate glass (product # CG-4920-01). The vial was placed approximately 3 cm away from the LED lamps, with the LEDs shining directly at the side of the vial as shown in following picture. Three reactions per lamp could be set up at the same time. And a fan above the reaction vials can keep the temperature around 35 °C. 10 mL microwave reaction vial secured by 20 mm aluminum seals with 0.125-inch thick, blue PTFE / white silicone septa was used for the reaction.

Instrumentation. For NMR spectrometry, NMR spectra were obtained on Bruker spectrometers operating at 400 or 500 MHz for ^{1}H NMR and 101 or 126 MHz for ^{13}C{1 H} NMR. The data were reported in the following order: chemical shifts (δ ppm), multiplicity (s = singlet, d = doublet, dd = doublet of doublets, t = triplet, q = quartet, m = multiplet), coupling constant, (Hz), relative integral made in reference to NMR solvent signals. For mass spectrometry, gas chromatograph−mass spectrometry was obtained using a Agilent GC System Intuvo 9000 Series coupled with a Agilent 5977B GC/MSD Mass Selective Detector. High resolution mass spectra were obtained using a Thermo Orbitrap Fusion Tribid Mass Spectrometer with electrospray ionization (ESI).

2. General Procedures

2.1 Synthesis of amide and ester derivatives of *N*-aryl glycine was performed according to previous reports.[17-19]

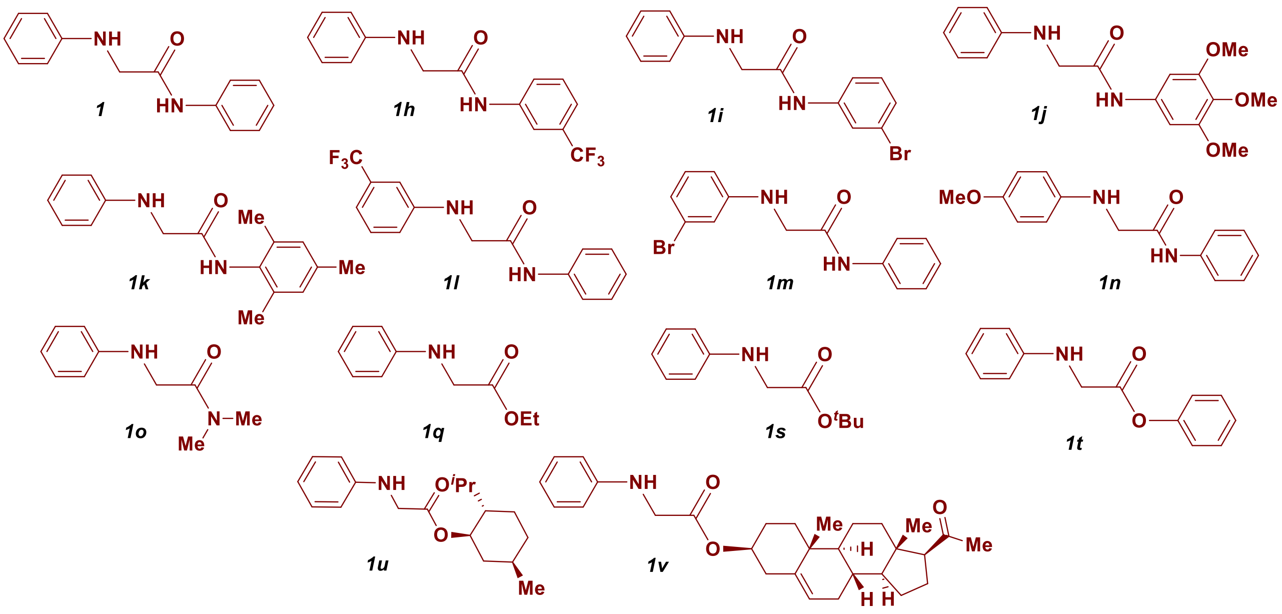

2.2 General procedure for the EDA complex-promoted cross-dehydrogenative coupling.

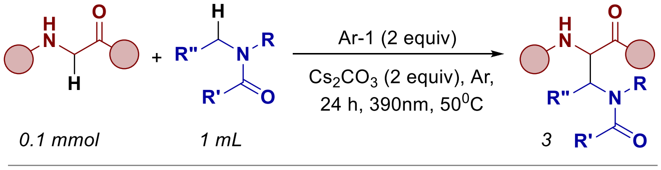

N-aryl glycine derivative (0.1 mmol, 1 equiv.), 4-iodobenzotrifluoride (**Ar-1**) (29.5 µL, 0.2 mmol, 2 equiv.), Cs_2CO_3 (65.0 mg, 0.20 mmol, 2.0 equiv.) , and amide derivatives (1.0 mL) were mixed in a 10 mL microwave vial equipped with a stir bar under argon atmosphere. The vial was sealed with a septum-cap and placed 3 cm away from two 390 nm blue LED (40W). The temperature was kept at approximately 50°C (heating caused by LED lamp). After being stirred for 24 hours, the reaction mixture was poured into 20 mL of water, and the resulting mixture was extracted with ethyl acetate (3 × 20 mL). The combined organic phase was dried over Na_2SO_4, filtered, and concentrated under vacuum. The residue was further purified by flash column chromatography on silica gel.

3. Optimization Study:

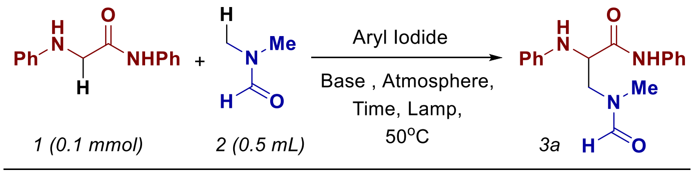

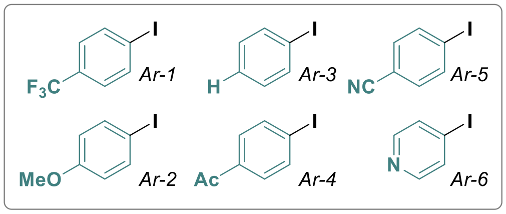

Table S1. ^{1}HNMR yields using 1,2-dibromoethane as internal standard.

Entry	Solvent	Base	Aryl Iodide	Lamp	Atmosphere	Time	Yield (3a)
1	Acetone (0.5 mL)	Cs_2CO_3 (2 equiv)	Ar-1 (2 equiv.)	390 nm	Argon	24 h	27%
2	MeOH (0.5 mL)	Cs_2CO_3 (2 equiv)	Ar-1 (2 equiv.)	390 nm	Argon	24 h	18%
3	DMSO (0.5 mL)	Cs_2CO_3 (2 equiv)	Ar-1 (2 equiv.)	390 nm	Argon	24 h	35%
4	DCM (0.5 mL)	Cs_2CO_3 (2 equiv)	Ar-1 (2 equiv.)	390 nm	Argon	24 h	27%
5	DMF (0.5 mL)	Cs_2CO_3 (2 equiv)	Ar-1 (2 equiv.)	390 nm	Argon	24 h	50%
6	MeCN (0.5 mL)	Cs_2CO_3 (2 equiv)	Ar-1 (2 equiv.)	390 nm	Argon	24 h	19%
7	$CHCl_3$ (0.5 mL)	Cs_2CO_3 (2 equiv)	Ar-1 (2 equiv.)	390 nm	Argon	24 h	Trace
8	H_2O (0.5 mL)	Cs_2CO_3 (2 equiv)	Ar-1 (2 equiv.)	390 nm	Argon	24 h	Trace
9	Ethyl Acetate (0.5 mL)	Cs_2CO_3 (2 equiv)	Ar-1 (2 equiv.)	390 nm	Argon	24 h	32%
10	DMF (0.5 mL)	DIPEA (2 equiv)	Ar-1 (2 equiv.)	390 nm	Argon	24 h	6%
11	DMF (0.5 mL)	Et_3N (2 equiv)	Ar-1 (2 equiv.)	390 nm	Argon	24 h	13%
12	DMF (0.5 mL)	TMG (2 equiv)	Ar-1 (2 equiv.)	390 nm	Argon	24 h	25%
13	DMF (0.5 mL)	DBU (2 equiv)	Ar-1 (2 equiv.)	390 nm	Argon	24 h	30%
14	DMF (0.5 mL)	K_2CO_3 (2 equiv)	Ar-1 (2 equiv.)	390 nm	Argon	24 h	14%
15	DMF (0.5 mL)	Na_2CO_3 (2 equiv)	Ar-1 (2 equiv.)	390 nm	Argon	24 h	10%
16	DMF (0.5 mL)	Na_2HPO_4 (2 equiv)	Ar-1 (2 equiv.)	390 nm	Argon	24 h	10%
17	DMF (0.5 mL)	K_2HPO_4 (2 equiv)	Ar-1 (2 equiv.)	390 nm	Argon	24 h	18%
18	DMF (0.5 mL)	w/o Base	Ar-1 (2 equiv.)	390 nm	Argon	24 h	Trace
19	DMF (0.5 mL)	Cs_2CO_3 (2 equiv)	Ar-2 (2 equiv.)	390 nm	Argon	24 h	32%
20	DMF (0.5 mL)	Cs_2CO_3 (2 equiv)	Ar-3 (2 equiv.)	390 nm	Argon	24 h	28%
21	DMF (0.5 mL)	Cs_2CO_3 (2 equiv)	Ar-4 (2 equiv.)	390 nm	Argon	24 h	35%
22	DMF (0.5 mL)	Cs_2CO_3 (2 equiv)	Ar-5 (2 equiv.)	390 nm	Argon	24 h	31%

23	DMF (0.5 mL)	Cs_2CO_3 (2 equiv)	Ar-6 (2 equiv.)	390 nm	Argon	24 h	45%
24	DMF (0.5 mL)	Cs_2CO_3 (2 equiv)	w/o Aryl Iodide	390 nm	Argon	24 h	N/R
25	DMF (0.5 mL)	Cs_2CO_3 (2 equiv)	Ar-1 (2 equiv.)	370 nm	Argon	24 h	36%
26	DMF (0.5 mL)	Cs_2CO_3 (2 equiv)	Ar-1 (2 equiv.)	427 nm	Argon	24 h	29%
27	DMF (0.5 mL)	Cs_2CO_3 (2 equiv)	Ar-1 (2 equiv.)	440 nm	Argon	24 h	22%
28	DMF (0.5 mL)	Cs_2CO_3 (2 equiv)	Ar-1 (2 equiv.)	456 nm	Argon	24 h	N/R
29	DMF (0.5 mL)	Cs_2CO_3 (2 equiv)	Ar-1 (2 equiv.)	Dark	Argon	24 h	N/R
30	DMF (0.5 mL)	Cs_2CO_3 (2 equiv)	Ar-1 (2 equiv.)	390 nm	Air	24 h	15%
31	DMF (0.5 mL)	Cs_2CO_3 (2 equiv)	Ar-1 (2 equiv.)	390 nm	Nitrogen	24 h	15%
32	DMF (0.5 mL)	Cs_2CO_3 (1 equiv)	Ar-1 (2 equiv.)	390 nm	Argon	24 h	35%
33	DMF (0.5 mL)	Cs_2CO_3 (1.5 equiv)	Ar-1 (2 equiv.)	390 nm	Argon	24 h	40%
34	DMF (0.5 mL)	Cs_2CO_3 (3 equiv)	Ar-1 (2 equiv.)	390 nm	Argon	24 h	50%
35	DMF (0.5 mL)	Cs_2CO_3 (2 equiv)	Ar-1 (1 equiv.)	390 nm	Argon	24 h	26%
36	DMF (0.5 mL)	Cs_2CO_3 (2 equiv)	Ar-1 (3 equiv.)	390 nm	Argon	24 h	50%
37	DMF (0.5 mL)	Cs_2CO_3 (2 equiv)	Ar-1 (2 equiv.)	390 nm	Argon	16 h	43%
38	DMF (0.5 mL)	Cs_2CO_3 (2 equiv)	Ar-1 (2 equiv.)	390 nm	Argon	48 h	50%

4. Unsuccessful Substrates:

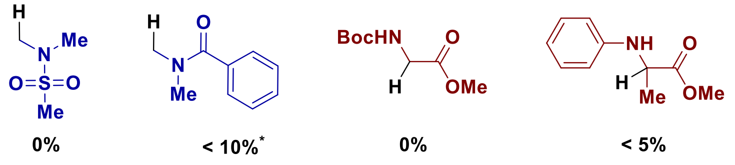

* 5 equiv. amide derivative in 1 mL MeCN

5. Experimental Details and Characterization Data for Products:

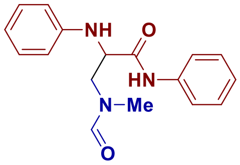

3-(*N*-methylformamido)-*N*-phenyl-2-(phenylamino)propanamide (3a): Reaction using amide derivative *N*,*N*-dimethylformamide (1.0 mL), and *N*-aryl glycine derivative **1** (22.6 mg, 0.1 mmol, 1 equiv.), under standard condition. After being stirred for 24 hours, the reaction mixture was poured into 20 mL of water, and the resulting mixture was extracted with ethyl acetate (3 × 20 mL). The combined organic phase was dried over Na_2SO_4, filtered, and concentrated under vacuum. The residue was further purified by flash column, eluting with ethyl acetate:hexane (1:5) as yellow oil 14.8 mg, 50% yield.

^{1}H NMR (400 MHz, $CDCl_3$) δ 8.99 (s, 1H), 8.13 (s, 1H), 7.57 – 7.51 (m, 2H), 7.35 – 7.29 (m, 2H), 7.24 – 7.18 (m, 2H), 7.15 – 7.09 (m, 1H), 6.87 – 6.80 (m, 1H), 6.68 – 6.62 (m, 2H), 4.22 (dd, J = 14.2, 9.2 Hz, 1H), 3.95 (dd, J = 9.2, 2.8 Hz, 1H), 3.61 (dd, J = 14.2, 2.8 Hz, 1H), 3.03 (s, 3H).

^{13}C NMR (101 MHz, $CDCl_3$) δ 169.5, 165.2, 146.8, 137.1, 129.5, 129.1, 124.8, 119.9, 119.7, 113.9, 61.6, 48.1, 35.9.

HRMS (ESI-TOF) m/z: [M + H]+ calcd. for $C_{17}H_{20}N_3O_2$ 298.1556, found 298.1554.

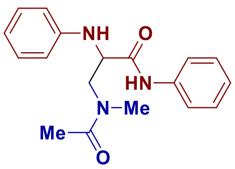

3-(*N*-methylacetamido)-*N*-phenyl-2-(phenylamino)propanamide (3b): Reaction using amide derivative *N*,*N*-dimethylacetamide (1.0 mL), and *N*-aryl glycine derivative **1** (22.6 mg, 0.1 mmol, 1 equiv.), under standard condition. After being stirred for 24 hours, the reaction mixture was poured into 20 mL of water, and the resulting mixture was extracted with ethyl acetate (3 × 20 mL). The combined organic phase was dried over Na_2SO_4, filtered, and concentrated under vacuum. The residue was further purified by flash column, eluting with ethyl acetate:hexane (1:4) as yellow solid 15.5 mg, 50% yield.

^{1}H NMR (400 MHz, $CDCl_3$) δ 8.99 (s, 1H), 7.54 (d, J = 7.9 Hz, 2H), 7.31 (t, J = 7.8 Hz, 2H), 7.20 (t, J = 7.8 Hz, 2H), 7.11 (t, J = 7.4 Hz, 1H), 6.82 (t, J = 7.3 Hz, 1H), 6.65 (d, J = 8.0 Hz, 2H), 5.90 (s, 1H), 4.31 (dd, J = 14.1, 9.3 Hz, 1H), 3.92 (m, 1H), 3.55 (m, 1H), 3.07 (s, 3H), 2.11 (s, 3H).

^{13}C NMR (101 MHz, $CDCl_3$) δ 173.8, 169.8, 147.2, 137.2, 129.5, 129.0, 124.7, 119.8, 119.4, 113.9, 62.3, 51.0, 37.4, 21.8.

HRMS (ESI-TOF) m/z: $[M + Na]^+$ calcd. for $C_{18}H_{21}N_3O_2Na$ 334.1531, found 334.1535.

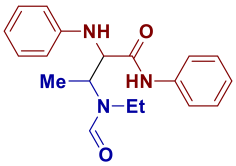

3-(*N*-ethylformamido)-*N*-phenyl-2-(phenylamino)butanamide (3c): Reaction using amide derivative *N*,*N*-diethylformamide (1.0 mL), and *N*-aryl glycine derivative **1** (22.6 mg, 0.1 mmol, 1 equiv.), under standard condition. After being stirred for 24 hours, the reaction mixture was poured into 20 mL of water, and the resulting mixture was extracted with ethyl acetate (3 × 20 mL). The combined organic phase was dried over Na_2SO_4, filtered, and concentrated under vacuum. The residue was further purified by flash column, eluting with ethyl acetate:hexane (1:5) as light yellow solid 18.2 mg, 56% yield (d.r, 1:1).

^{1}H NMR (400 MHz, $CDCl_3$) δ 8.89 (s, 1H), 7.53 – 7.48 (m, 2H), 7.33 – 7.20 (m, 6H), 7.13 – 7.07 (m, 1H), 6.90 – 6.83 (m, 1H), 6.81 – 6.75 (m, 2H), 4.85 (s, 1H), 4.22 – 4.05 (m, 1H), 3.64 – 3.44 (m, 2H), 3.39 – 3.25 (m, 1H), 1.23 – 1.18 (m, 3H), 1.18 – 1.12 (m, 3H).

^{13}C NMR (101 MHz, $CDCl_3$) δ 173.1, 170.5, 147.4, 137.3, 129.5, 128.9, 124.5, 119. 9, 118.8, 113.6, 65.9, 55.9, 22.6, 16.4, 14.7.

HRMS (ESI-TOF) m/z: $[M + H]^+$ calcd. for $C_{19}H_{24}N_3O_2$ 326.1869, found 326.1871.

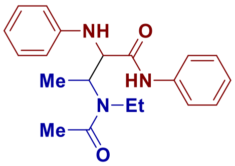

3-(*N*-ethylacetamido)-*N*-phenyl-2-(phenylamino)butanamide (3d): Reaction using amide derivative *N*,*N*-diethylacetamide (1.0 mL), and *N*-aryl glycine derivative **1** (22.6 mg, 0.1 mmol, 1 equiv.), under standard condition. After being stirred for 24 hours, the reaction mixture was poured into 20 mL of water, and the resulting mixture was extracted with ethyl acetate (3 × 20 mL). The combined organic phase was dried over Na_2SO_4, filtered, and concentrated under vacuum. The residue was further purified by flash column, eluting with ethyl acetate:hexane (1:10) as light yellow solid 17.6 mg, 52% yield (d.r, 1: 1.1).

^{1}H NMR (400 MHz, $CDCl_3$) δ 8.85 (s, 1H), 7.51 (d, J = 8.0 Hz, 2H), 7.30 (t, J = 7.6 Hz, 2H), 7.18 (t, J = 7.9 Hz, 2H), 7.10 (t, J = 7.1 Hz, 1H), 6.78 (q, J = 7.2 Hz, 1H), 6.71 – 6.63 (m, 2H), 6.27 (s, 1H), 4.51 (p, J = 7.1 Hz, 1H), 4.09 (m, 1H), 3.89 (m, 1H), 3.30 (m, 2H), 2.17 (s, 1H), 2.11 (s, 3H), 1.58 (d, J = 7.0 Hz, 1H), 1.47 (d, J = 7.1 Hz, 1H), 1.26 (s, 1H), 1.18 (m, 3H).

^{13}C NMR (101 MHz, $CDCl_3$) δ 173.1 (172.9), 170.5 (170.1), 147.6 (147.4), 137.38 (137.35), 129.5 (129.4), 129.02 (128.98), 124.53, 119.9 (119.8), 119.10 (118.8), 114.2 (113.6), 65.9 (65.4), 22.8 (22.6), 16.4, 14.8 (14.7), 14.7.

HRMS (ESI-TOF) m/z: $[M + H]^+$ calcd. for $C_{20}H_{26}N_3O_2$ 340.2025, found 340.2028.

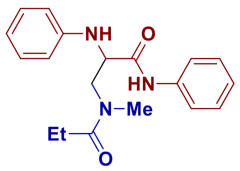

3-(*N*-methylpropionamido)-*N*-phenyl-2-(phenylamino)propanamide (3e): Reaction using amide derivative N,N-dimethylpropionamide (1.0 mL), and *N*-aryl glycine derivative **1** (22.6 mg, 0.1 mmol, 1 equiv.), under standard condition. After being stirred for 24 hours, the reaction mixture was poured into 20 mL of water, and the resulting mixture was extracted with ethyl acetate (3 × 20 mL). The combined organic phase was dried over Na_2SO_4, filtered, and concentrated under vacuum. The residue was further purified by flash column, eluting with ethyl acetate:hexane (1:10) as yellow solid 14.3 mg, 44% yield.

^{1}H NMR (400 MHz, $CDCl_3$) δ 8.98 (s, 1H), 7.54 (d, J = 7.8 Hz, 2H), 7.31 (t, J = 7.9 Hz, 2H), 7.20 (t, J = 7.9 Hz, 2H), 7.11 (t, J = 7.4 Hz, 1H), 6.81 (t, J = 7.3 Hz, 1H), 6.64 (d, J = 7.8 Hz, 2H), 5.92 (s, 1H), 4.32 (dd, J = 14.1, 9.2 Hz, 1H), 3.91 (m, 1H), 3.56 (m, 1H), 3.05 (s, 3H), 2.34 (m, 2H), 1.16 (t, J = 7.4 Hz, 3H).

^{13}C NMR (101 MHz, $CDCl_3$) δ 176.9, 169.9, 147.3, 137.3, 129.5, 129.0, 124.7, 119.8, 119.4, 113.9, 77.1, 62.4, 51.2, 36.5, 26.8, 9.2.

HRMS (ESI-TOF) m/z: $[M + Na]^+$ calcd. for $C_{19}H_{23}N_3O_2Na$ 348.1688, found 348.1689.

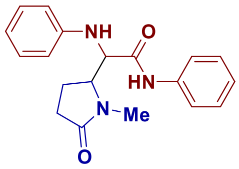

2-(1-methyl-5-oxopyrrolidin-2-yl)-*N*-phenyl-2-(phenylamino)acetamide (3f): Reaction using amide derivative 1-methylpyrrolidin-2-one (1.0 mL), and *N*-aryl glycine derivative **1** (22.6 mg, 0.1 mmol, 1 equiv.), under standard condition. After being stirred for 24 hours, the reaction mixture was poured into 20 mL of water, and the resulting mixture was extracted with ethyl acetate (3 × 20 mL). The combined organic phase was dried over Na_2SO_4, filtered, and concentrated under vacuum. The residue was further purified by flash column, eluting with ethyl acetate:hexane (1:6) as white solid 15.4 mg, 48% yield (d.r, 1:1).

^{1}H NMR (400 MHz, $CDCl_3$) δ 8.89 (s, 1H), 7.52 (d, J = 8.0 Hz, 2H), 7.33 (t, J = 7.9 Hz, 2H), 7.29 – 7.24 (m, 2H), 7.14 (t, J = 7.4 Hz, 1H), 6.89 (t, J = 7.4 Hz, 1H), 6.78 (d, J = 7.9 Hz, 2H), 4.66 – 4.59 (m, 1H), 4.44 (d, J = 6.9 Hz, 1H), 4.01 – 3.95 (m, 1H), 2.85 (s, 3H), 2.53 – 2.37 (m, 2H), 2.36 – 2.25 (m, 1H), 1.85 – 1.75 (m, 1H).

^{13}C NMR (101 MHz, $CDCl_3$) δ 176.5, 169.7, 146.9, 136.9, 129.9, 129.1, 124.9, 120.2, 119.9, 114.0, 77.0, 62.3, 61.2, 29.9, 29.3, 22.4.

^{1}H NMR (400 MHz, $CDCl_3$) δ 8.82 (s, 1H), 7.51 (d, J = 7.7 Hz, 2H), 7.33 (t, J = 7.9 Hz, 2H), 7.28 – 7.22 (m, 3H), 7.14 (t, J = 7.4 Hz, 1H), 6.90 (t, J = 7.4 Hz, 1H), 6.77 (d, J = 7.9 Hz, 2H), 4.39 – 4.34 (m, 1H), 4.34 – 4.30 (m, 1H), 4.14 – 4.10 (m, 1H), 2.72 (s, 3H), 2.56 – 2.38 (m, 2H), 2.22 – 2.10 (m, 1H), 2.06 – 1.93 (m, 1H).

^{13}C NMR (101 MHz, $CDCl_3$) δ 175.9, 168.6, 146.4, 136.9, 129.7, 129.1, 124.9, 120.7, 120.0, 114.5, 77.0, 61.1, 60.3, 30.1, 27.7, 18.9.

HRMS (ESI-TOF) m/z: $[M + H]^+$ calcd. for $C_{19}H_{22}N_3O_2$ 324.1712, found 324.1716.

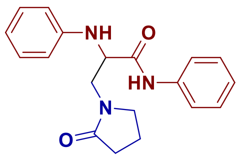

3-(2-oxopyrrolidin-1-yl)-*N*-phenyl-2-(phenylamino)propanamide (3f'): 2.0 mg, 7%.

^{1}H NMR (400 MHz, $CDCl_3$) δ 8.96 (s, 1H), 7.55 – 7.51 (m, 2H), 7.34 – 7.30 (m, 2H), 7.23 – 7.18 (m, 2H), 7.12 (t, *J* = 7.4 Hz, 1H), 6.83 (t, *J* = 7.3 Hz, 1H), 6.67 (d, *J* = 8.0 Hz, 2H), 5.68 – 5.60 (m, 1H), 4.07 – 3.98 (m, 1H), 3.96 – 3.90 (m, 1H), 3.72 – 3.65 (m, 1H), 3.55 – 3.40 (m, 2H), 2.46 – 2.34 (m, 2H), 2.06 – 1.92 (m, 2H).

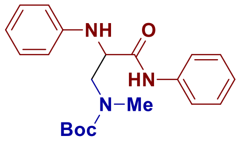

***tert*-butyl methyl(3-oxo-2,3-bis(phenylamino)propyl)carbamate (3g):** Reaction using amide derivative *tert*-butyl dimethylcarbamate (1.0 mL), and *N*-aryl glycine derivative **1** (22.6 mg, 0.1 mmol, 1 equiv.), under standard condition. After being stirred for 24 hours, the reaction mixture was poured into 20 mL of water, and the resulting mixture was extracted with ethyl acetate (3 × 20 mL). The combined organic phase was dried over Na_2SO_4, filtered, and concentrated under vacuum. The residue was further purified by flash column, eluting with ethyl acetate:hexane (1:10) as yellow solid 19.1 mg, 52% yield.

^{1}H NMR (400 MHz, $CDCl_3$) δ 8.96 (s, 1H), 7.53 (d, J = 7.7 Hz, 2H), 7.31 (t, J = 7.9 Hz, 2H), 7.21 (t, J = 7.7 Hz, 2H), 7.11 (t, J = 7.4 Hz, 1H), 6.83 (m, 1H), 6.64 (d, J = 7.8 Hz, 2H), 5.88 (s, 1H), 4.11 (m, 1H), 3.90 (m, 1H), 3.46 (d, J = 14.1 Hz, 1H), 2.91 (s, 3H), 1.50 (s, 9H).

^{13}C NMR (101 MHz, $CDCl_3$) δ 170.0, 158.3, 147.4, 137.3, 129.5, 129.0, 124.6, 119.8, 119.4, 113.8, 80.9, 62.4, 51.5, 35.4, 28.3.

HRMS (ESI-TOF) m/z: $[M + H]^+$ calcd. for $C_{21}H_{28}N_3O_3$ 370.2131, found 370.2130.

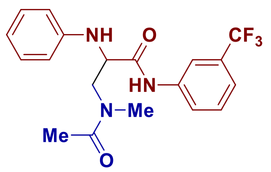

3-(*N*-methylacetamido)-2-(phenylamino)-*N*-(3-(trifluoromethyl)phenyl)propanamide (3h): Reaction using amide derivative *N*,*N*-dimethylacetamide (1.0 mL), and *N*-aryl glycine derivative **1h** (29.4 mg, 0.1 mmol, 1 equiv.), under standard condition. After being stirred for 24 hours, the reaction mixture was poured into 20 mL of water, and the resulting mixture was extracted with ethyl acetate (3 × 20 mL). The combined organic phase was dried over Na_2SO_4, filtered, and concentrated under vacuum. The residue was further purified by flash column, eluting with ethyl acetate:hexane (1:6) as yellow oil 15.1 mg, 40% yield.

^{1}H NMR (400 MHz, $CDCl_3$) δ 9.17 (s, 1H), 7.92 (s, 1H), 7.69 (d, J = 7.9 Hz, 1H), 7.43 (t, J = 7.9 Hz, 1H), 7.37 (m, 1H), 7.21 (t, J = 7.9 Hz, 2H), 6.84 (t, J = 7.3 Hz, 1H), 6.65 (d, J = 7.9 Hz, 2H), 5.91 (s, 1H), 4.33 (dd, J = 14.1, 9.4 Hz, 1H), 3.94 (d, J = 9.3 Hz, 1H), 3.52 (m, 1H), 3.07 (s, 3H), 2.11 (s, 3H).

^{13}C NMR (101 MHz, $CDCl_3$) δ 173.8, 170.3, 147.0, 137.8, 131.4 (q, J = 32.5 Hz), 129.6, 123.8 (q, J = 272.2 Hz) 122.8, 121.2 (q, J = 3.9 Hz), 119.7, 116.5 (q, J = 3.9 Hz), 113.9, 62.2, 51.0, 37.4, 29.7, 21.8.

^{19}F NMR (376 MHz, $CDCl_3$) δ -62.78.

HRMS (ESI-TOF) m/z: $[M + H]^+$ calcd. for $C_{19}H_{21}F_3N_3O_2$ 380.1586, found 380.1581.

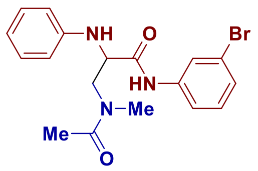

***N*-(3-bromophenyl)-3-(*N*-methylacetamido)-2-(phenylamino)propanamide (3i):** Reaction using amide derivative *N*,*N*-dimethylacetamide (1.0 mL), and *N*-aryl glycine derivative **1i** (30.5 mg, 0.1 mmol, 1 equiv.), under standard condition. After being stirred for 24 hours, the reaction mixture was poured into 20 mL of water, and the resulting mixture was extracted with ethyl acetate (3 × 20 mL). The combined organic phase was dried over Na_2SO_4, filtered, and concentrated under vacuum. The residue was further purified by flash column, eluting with ethyl acetate:hexane (1:6) as light brown oil 15.5 mg, 40% yield.

^{1}H NMR (400 MHz, $CDCl_3$) δ 8.83 (s, 1H), 7.53 (d, J = 7.9 Hz, 2H), 7.33 (t, J = 7.9 Hz, 2H), 7.13 (t, J = 7.4 Hz, 1H), 7.04 (t, J = 8.0 Hz, 1H), 6.93 (d, J = 8.0 Hz, 1H), 6.81 (s, 1H), 6.54 (m, 1H), 6.12 – 6.09 (m, 1H), 4.25 (dd, J = 14.2, 9.0 Hz, 1H), 3.90 (m, 1H), 3.58 (m, 1H), 3.08 (s, 3H), 2.12 (s, 3H).

^{13}C NMR (101 MHz, $CDCl_3$) δ 174.0, 169.2, 148.4, 137.1, 130.8, 129.1, 124.8, 123.4, 122.3, 119.9, 116.8, 112.2, 62.1, 51.2, 37.6, 21.8.

HRMS (ESI-TOF) m/z: $[M + Na]^+$ calcd. for $C_{18}H_{20}BrN_3O_2Na$ 412.0637, found 412.0633.

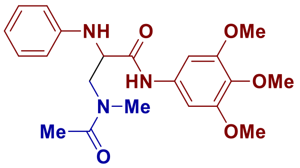

3-(*N*-methylacetamido)-2-(phenylamino)-*N*-(3,4,5-trimethoxyphenyl)propanamide (3j): Reaction using amide derivative *N*,*N*-dimethylacetamide (1.0 mL), and *N*-aryl glycine derivative **1j** (31.6 mg, 0.1 mmol, 1 equiv.), under standard condition. After being stirred for 24 hours, the reaction mixture was poured into 20 mL of water, and the resulting mixture was extracted with ethyl acetate (3 × 20 mL). The combined organic phase was dried over Na_2SO_4, filtered, and concentrated under vacuum. The residue was further purified by flash column, eluting with ethyl acetate:hexane (1:10) as yellow solid 20.0 mg, 50% yield.

^{1}H NMR (400 MHz, $CDCl_3$) δ 8.94 (s, 1H), 7.21 (t, J = 7.9 Hz, 2H), 6.87 (s, 2H), 6.83 (t, J = 7.3 Hz, 1H), 6.65 (d, J = 7.8 Hz, 2H), 5.87 (s, 1H), 4.33 (dd, J = 14.1, 9.6 Hz, 1H), 3.89 (m, 1H), 3.83 (s, 6H), 3.79 (s, 3H), 3.48 (m, 1H), 3.06 (s, 3H), 2.10 (s, 3H).

^{13}C NMR (101 MHz, $CDCl_3$) δ 173.8, 169.8, 153.4, 147.2, 134.9, 133.4, 129.5, 119.6, 114.0, 97.3, 62.5, 61.0, 56.2, 51.0, 37.4, 21.8.

HRMS (ESI-TOF) m/z: $[M + H]^+$ calcd. for $C_{21}H_{28}N_3O_5$ 402.2029, found 402.2032.

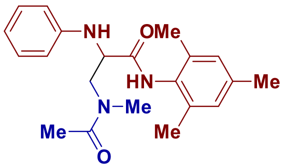

***N*-mesityl-3-(*N*-methylacetamido)-2-(phenylamino)propanamide (3k):** Reaction using amide derivative *N*,*N*-dimethylacetamide (1.0 mL), and *N*-aryl glycine derivative **1k** (26.8 mg, 0.1 mmol, 1 equiv.), under standard condition. After being stirred for 24 hours, the reaction mixture was poured into 20 mL of water, and the resulting mixture was extracted with ethyl acetate (3 × 20 mL). The combined organic phase was dried over Na_2SO_4, filtered, and concentrated under vacuum. The residue was further purified by flash column, eluting with ethyl acetate:hexane (1:10) as white solid 11.6 mg, 33% yield.

^{1}H NMR (400 MHz, $CDCl_3$) δ 8.46 (s, 1H), 7.22 (t, J = 7.9 Hz, 2H), 6.85 (s, 2H), 6.81 (t, J = 7.3 Hz, 1H), 6.70 (d, J = 7.8 Hz, 2H), 5.96 (s, 1H), 4.50 (dd, J = 14.0, 10.3 Hz, 1H), 4.02 (m, 1H), 3.41 (m, 1H), 3.09 (s, 3H), 2.24 (s, 3H), 2.12 (s, 3H), 2.10 (s, 6H).

^{13}C NMR (101 MHz, $CDCl_3$) δ 173.7, 170.0, 147.1, 137.0, 134.8, 130.5, 129.4, 129.0, 119.1, 113.7, 61.5, 51.0, 37.3, 21.8, 20.9, 18.4.

HRMS (ESI-TOF) m/z: $[M + H]^+$ calcd. for $C_{21}H_{28}N_3O_2$ 354.2182, found 354.2180.

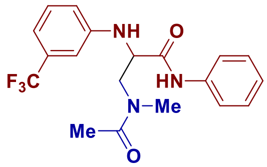

3-(*N*-methylacetamido)-*N*-phenyl-2-((3-(trifluoromethyl)phenyl)amino)propanamide (3l): Reaction using amide derivative *N*,*N*-dimethylacetamide (1.0 mL), and *N*-aryl glycine derivative **1l** (29.4 mg, 0.1 mmol, 1 equiv.), under standard condition. After being stirred for 24 hours, the reaction mixture was poured into 20 mL of water, and the resulting mixture was extracted with ethyl acetate (3 × 20 mL). The combined organic phase was dried over Na_2SO_4, filtered, and concentrated under vacuum. The residue was further purified by flash column, eluting with ethyl acetate:hexane (1:6) as yellow oil 9.4 mg, 25% yield.

^{1}H NMR (400 MHz, $CDCl_3$) δ 8.84 (s, 1H), 7.52 (d, J = 7.9 Hz, 2H), 7.36 – 7.28 (m, 3H), 7.13 (t, J = 7.4 Hz, 1H), 7.05 (d, J = 7.7 Hz, 1H), 6.88 (s, 1H), 6.76 (d, J = 8.1 Hz, 1H), 6.26 (s, 1H), 4.24 (dd, J = 14.2, 8.8 Hz, 1H), 3.99 – 3.93 (m, 1H), 3.64 (m, 1H), 3.08 (s, 3H), 2.12 (s, 3H).

^{13}C NMR (101 MHz, $CDCl_3$) δ 174.1, 169.2, 147.4, 137.0, 131.9 (q, J = 32.0 Hz), 130.0, 129.1, 124.9, 124.0 (q, J = 272.6 Hz), 120.0, 119.8, 116.4, 115.8 (q, J = 3.9 Hz), 110.5 (q, J = 3.9 Hz), 62.0, 51.3, 37.7, 29.7, 21.8.

HRMS (ESI-TOF) m/z: $[M + H]^+$ calcd. for $C_{19}H_{21}F_3N_3O_2$ 380.1586, found 380.1580.

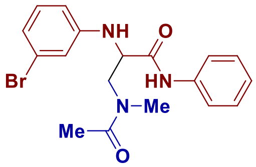

2-((3-bromophenyl)amino)-3-(*N*-methylacetamido)-*N*-phenylpropanamide (3m): Reaction using amide derivative *N*,*N*-dimethylacetamide (1.0 mL), and *N*-aryl glycine derivative **1m** (30.5 mg, 0.1 mmol, 1 equiv.), under standard condition. After being stirred for 24 hours, the reaction mixture was poured into 20 mL of water, and the resulting mixture was extracted with ethyl acetate (3 × 20 mL). The combined organic phase was dried over Na_2SO_4, filtered, and concentrated under vacuum. The residue was further purified by flash column, eluting with ethyl acetate:hexane (1:8) as yellow solid 19.8 mg, 51% yield.

^{1}H NMR (400 MHz, $CDCl_3$) δ 8.86 (s, 1H), 7.53 (d, J = 7.8 Hz, 2H), 7.32 (t, J = 7.9 Hz, 2H), 7.12 (t, J = 7.4 Hz, 1H), 7.03 (t, J = 8.0 Hz, 1H), 6.92 (d, J = 7.9 Hz, 1H), 6.81 (m, 1H), 6.54 (m, 1H), 6.08 (m, 1H), 4.24 (dd, J = 14.2, 9.0 Hz, 1H), 3.90 (m, 1H), 3.58 (dd, J = 14.2, 2.7 Hz, 1H), 3.07 (s, 3H), 2.11 (s, 3H).

^{13}C NMR (101 MHz, $CDCl_3$) δ 173.9, 169.3, 148.4, 137.1, 130.8, 129.1, 124.8, 123.4, 122.3, 119.9, 116.8, 112.2, 61.9, 51.2, 37.6, 21.8.

HRMS (ESI-TOF) m/z: $[M + Na]^+$ calcd. for $C_{18}H_{20}BrN_3O_2Na$ 412.0637, found 412.0635.

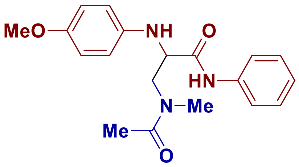

2-((4-methoxyphenyl)amino)-3-(*N*-methylacetamido)-*N*-phenylpropanamide (3n): Reaction using amide derivative *N*,*N*-dimethylacetamide (1.0 mL), and *N*-aryl glycine derivative **1n** (34.1 mg, 0.1 mmol, 1 equiv.), under standard condition. After being stirred for 24 hours, the reaction mixture was poured into 20 mL of water, and the resulting mixture was extracted with ethyl acetate (3 × 20 mL). The combined organic phase was dried over Na_2SO_4, filtered, and concentrated under vacuum. The residue was further purified by flash column, eluting with ethyl acetate:hexane (1:5) as yellow solid 11.9 mg, 35% yield.

^{1}H NMR (400 MHz, $CDCl_3$) δ 8.86 (s, 1H), 7.53 (d, *J* = 7.8 Hz, 2H), 7.32 (t, J = 7.9 Hz, 2H), 7.12 (t, J = 7.4 Hz, 1H), 7.03 (t, J = 8.0 Hz, 1H), 6.92 (d, *J* = 7.9 Hz, 1H), 6.81 (m, 1H), 6.54 (m, 1H), 6.08 (m, 1H), 4.24 (dd, *J* = 14.2, 9.0 Hz, 1H), 3.90 (m, 1H), 3.58 (dd, *J* = 14.2, 2.7 Hz, 1H), 3.07 (s, 3H), 2.11 (s, 3H).

^{13}C NMR (101 MHz, $CDCl_3$) δ 173.9, 169.3, 148.4, 137.1, 130.8, 129.1, 124.8, 123.4, 122.3, 119.9, 116.8, 112.2, 61.9, 51.2, 37.6, 21.8.

HRMS (ESI-TOF) m/z: $[M + H]^+$ calcd. for $C_{19}H_{24}BrN_3O_3$ 342.1818, found 342.1815.

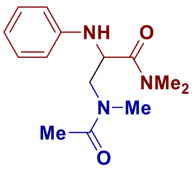

***N*,*N*-dimethyl-3-(*N*-methylacetamido)-2-(phenylamino)propanamide (3o):** Reaction using amide derivative *N*,*N*-dimethylacetamide (1.0 mL), and *N*-aryl glycine derivative **1o** (17.8 mg, 0.1 mmol, 1 equiv.), under standard condition. After being stirred for 24 hours, the reaction mixture was poured into 20 mL of water, and the resulting mixture was extracted with ethyl acetate (3 × 20 mL). The combined organic phase was dried over Na_2SO_4, filtered, and concentrated under vacuum. The residue was further purified by flash column, eluting with ethyl acetate:hexane (1:6) as light yellow oil 13.4 mg, 51% yield.

^{1}H NMR (400 MHz, $CDCl_3$) δ 7.14 (t, J = 7.8 Hz, 2H), 6.69 (t, J = 7.3 Hz, 1H), 6.64 (d, J = 8.0 Hz, 2H), 4.92 – 4.84 (m, 1H), 4.73 (s, 1H), 3.85 (dd, J = 13.4, 3.9 Hz, 1H), 3.23 (s, 3H), 3.11 (dd, J = 13.6, 9.3 Hz, 1H), 3.03 (s, 3H), 2.96 (s, 3H), 2.06 (s, 3H).

^{13}C NMR (101 MHz, $CDCl_3$) δ 171.8, 171.3, 147.5, 129.4, 117.9, 113.4, 77.0, 52.8, 52.8, 39.2, 37.1, 35.8, 21.9.

HRMS (ESI-TOF) m/z: $[M + H]^+$ calcd. for $C_{14}H_{22}N_3O_2$ 364.1712, found 364.1710.

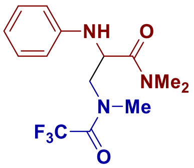

***N*,*N*-dimethyl-2-(phenylamino)-3-(2,2,2-trifluoro-*N*-methylacetamido)propanamide (3p):** Reaction using amide derivative 2,2,2-trifluoro-*N*,*N*-dimethylacetamide (1.0 mL), and *N*-aryl glycine derivative **1o** (17.8 mg, 0.1 mmol, 1 equiv.), under standard condition. After being stirred for 24 hours, the reaction mixture was poured into 20 mL of water, and the resulting mixture was extracted with ethyl acetate (3 × 20 mL). The combined organic phase was dried over Na_2SO_4, filtered, and concentrated under vacuum. The residue was further purified by flash column, eluting with ethyl acetate:hexane (1:4) as colorless oil 5.7 mg, 18% yield.

^{1}H NMR (400 MHz, $CDCl_3$) δ 7.20 – 7.15 (m, 2H), 6.78 – 6.73 (m, 1H), 6.69 – 6.64 (m, 2H), 4.93 – 4.83 (m, 1H), 4.74 – 4.60 (m, 1H), 3.81 (dd, J = 13.3, 4.6 Hz, 1H), 3.41 – 3.33 (m, 1H), 3.22 (d, J = 1.6 Hz, 3H), 3.19 (s, 3H), 2.98 (s, 3H).

^{13}C NMR (101 MHz, $CDCl_3$) δ 170.6, 146.9, 129.5, 129.3, 118.7, 113.7, 112.9, 54.1, 52.1, 37.1, 35.9, 29.7.

^{19}F NMR (376 MHz, $CDCl_3$) δ -70.28.

HRMS (ESI-TOF) m/z: $[M + H]^+$ calcd. for $C_{14}H_{19}F_3N_3O_2$ 318.1429, found 318.1425.

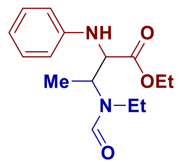

ethyl 3-(*N*-ethylformamido)-2-(phenylamino)butanoate (3q): Reaction using amide derivative *N*,*N*-diethylformamide (1.0 mL), and *N*-aryl glycine derivative **1q** (17.9 mg, 0.1 mmol, 1 equiv.), under standard condition. After being stirred for 24 hours, the reaction mixture was poured into 20 mL of water, and the resulting mixture was extracted with ethyl acetate (3 × 20 mL). The combined organic phase was dried over Na_2SO_4, filtered, and concentrated under vacuum. The residue was further purified by flash column, eluting with ethyl acetate:hexane (1:8) as yellow oil 11.9 mg, 43% yield (d.r, 1:1).

^{1}H NMR (400 MHz, $CDCl_3$) δ 8.19 – 8.04 (m, 1H), 7.22 – 7.12 (m, 2H), 6.82 – 6.69 (m, 1H), 6.68 – 6.60 (m, 2H), 4.63 – 4.48 (m, 1H), 4.18 (m, 3H), 3.94 – 3.77 (m, 1H), 3.40 – 3.18 (m, 2H), 1.46 (m, 1H), 1.41 (m, 1H), 1.34 (m, 1H), 1.29 – 1.11 (m, 6H).

^{13}C NMR (101 MHz, $CDCl_3$) δ 172.5 (172.1), 164.3 (163.5), 162.8 (162.7), 146.9 (146.7), 146.4 (146.3), 129.50 (129.44), 129.35 (129.31), 119.4 (119.3), 118.8 (118.3), 114.1 (114.0), 113.9 (113.4), 61.84 (61.82), 61.5 (61.4), 61.0 (60.9), 59.67, 56.5 (56.0), 52.5, 50.5, 41.50, 40.41, 37.1 (36.8), 17.4, 16.9, 16.7 (16.6), 15.5, 14.46, 14.20 (14.16), 14.1 (14.0).

HRMS (ESI-TOF) m/z: $[M + H]^+$ calcd. for $C_{15}H_{23}N_2O_3$ 279.1709, found 279.1712.

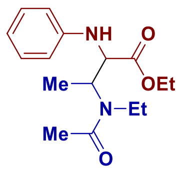

ethyl 3-(*N*-ethylacetamido)-2-(phenylamino)butanoate (3r): Reaction using amide derivative *N*,*N*-diethylacetamide (1.0 mL), and *N*-aryl glycine derivative **1q** (17.9 mg, 0.1 mmol, 1 equiv.), under standard condition. After being stirred for 24 hours, the reaction mixture was poured into 20 mL of water, and the resulting mixture was extracted with ethyl acetate (3 × 20 mL). The combined organic phase was dried over Na_2SO_4, filtered, and concentrated under vacuum. The residue was further purified by flash column, eluting with ethyl acetate:hexane (1:8) as brown oil 8.7 mg, 30% yield (d.r, 1:1).

^{1}H NMR (400 MHz, $CDCl_3$) δ 7.23 – 7.07 (m, 2H), 6.84 – 6.58 (m, 3H), 4.95 – 4.79 (m, 1H), 4.50 (s, 1H), 4.23 – 4.08 (m, 3H), 3.43 – 3.31 (m, 1H), 3.28 – 3.16 (m, 1H), 2.17 – 2.02 (m, 3H), 1.45 – 1.13 (m, 12H).

^{13}C NMR (101 MHz, $CDCl_3$) δ 172.6 (172.3), 146.8, 129.3 (129.3), 119.3, 118.5 (118.0), 114.0 (113.9), 113.7 (113.2), 61.3 (61.2), 59.6 (51.3), 42.8, 39.9 (39.8), 22.3 (22.0), 21.4, 15.9 (15.8), 14.8 (14.2), 14.1 (13.1).

HRMS (ESI-TOF) m/z: $[M + Na]^+$ calcd. for $C_{16}H_{24}N_2O_3Na$ 315.1685, found 315.1684.

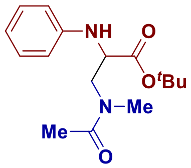

***tert*-butyl-3-(*N*-methylacetamido)-2-(phenylamino)propanoate (3s):** Reaction using amide derivative *N*,*N*-dimethylacetamide (1.0 mL), and *N*-aryl glycine derivative **1s** (20.7 mg, 0.1 mmol, 1 equiv.), under standard condition. After being stirred for 24 hours, the reaction mixture was poured into 20 mL of water, and the resulting mixture was extracted with ethyl acetate (3 × 20 mL). The combined organic phase was dried over Na_2SO_4, filtered, and concentrated under vacuum. The residue was further purified by flash column, eluting with ethyl acetate:hexane (1:8) as brown solid 5.8 mg, 20% yield.

^{1}H NMR (400 MHz, $CDCl_3$) δ 7.18 – 7.12 (m, 2H), 6.74 – 6.67 (m, 1H), 6.63 – 6.58 (m, 2H), 4.59 (s, 1H), 4.27 – 4.19 (m, 1H), 3.96 (dd, J = 13.7, 8.6 Hz, 1H), 3.50 (dd, J = 13.8, 5.2 Hz, 1H), 3.03 (s, 3H), 2.06 (s, 3H), 1.43 (s, 9H).

^{13}C NMR (101 MHz, $CDCl_3$) δ 171.9, 171.6, 146.9, 129.2, 117.9, 113.1, 82.1, 56.6, 50.0, 37.4, 27.9, 21.8.

HRMS (ESI-TOF) m/z: $[M + H]^+$ calcd. for $C_{16}H_{25}N_2O_3$ 293.1865, found 293.1860.

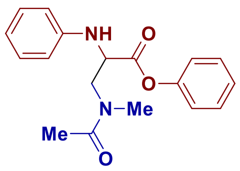

phenyl 3-(*N*-methylacetamido)-2-(phenylamino)propanoate (3t): Reaction using amide derivative *N*,*N*-dimethylacetamide (1.0 mL), and *N*-aryl glycine derivative **1t** (22.7 mg, 0.1 mmol, 1 equiv.), under standard condition. After being stirred for 24 hours, the reaction mixture was poured into 20 mL of water, and the resulting mixture was extracted with ethyl acetate (3 × 20 mL). The combined organic phase was dried over Na_2SO_4, filtered, and concentrated under vacuum. The residue was further purified by flash column, eluting with ethyl acetate:hexane (1:8) as yellow oil 13.7 mg, 44% yield.

^{1}H NMR (400 MHz, $CDCl_3$) δ 9.02 (s, 1H), 7.54 (d, J = 7.9 Hz, 2H), 7.31 (t, J = 7.9 Hz, 2H), 7.20 (t, J = 7.9 Hz, 2H), 7.11 (t, J = 7.5 Hz, 1H), 6.83 (t, J = 7.4 Hz, 1H), 6.66 (d, J = 8.1 Hz, 2H), 4.31 (dd, J = 14.2, 9.2 Hz, 1H), 3.93 (dd, J = 9.2, 2.9 Hz, 1H), 3.56 (dd, J = 14.2, 2.9 Hz, 1H), 3.07 (s, 3H), 2.11 (s, 3H).

^{13}C NMR (101 MHz, $CDCl_3$) δ 173.8, 169.7, 146.9, 137.2, 129.5, 129.0, 124.7, 119.8, 119.6, 114.0, 62.4, 51.0, 37.5, 21.8.

HRMS (ESI-TOF) m/z: $[M + H]^+$ calcd. for $C_{18}H_{21}N_2O_3$ 313.1552, found 313.1550.

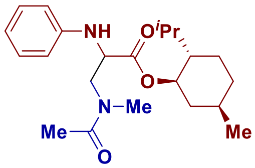

(1*R*,2*S*,5*R*)-2-isopropyl-5-methylcyclohexyl-3-(*N*-methylacetamido)-2-(phenylamino) propanoate (3u): Reaction using amide derivative *N*,*N*-dimethylacetamide (1.0 mL), and *N*-aryl glycine derivative **1u** (28.9 mg, 0.1 mmol, 1 equiv.), under standard condition. After being stirred for 24 hours, the reaction mixture was poured into 20 mL of water, and the resulting mixture was extracted with ethyl acetate (3 × 20 mL). The combined organic phase was dried over Na_2SO_4, filtered, and concentrated under vacuum. The residue was further purified by flash column, eluting with ethyl acetate:hexane (1:3) as yellow solid 13.8 mg, 37% yield (d.r, 1:1).

^{1}H NMR (400 MHz, $CDCl_3$) δ 7.22 – 7.08 (m, 2H), 6.81 – 6.67 (m, 1H), 6.66 – 6.54 (m, 2H), 4.79 – 4.57 (m, 2H), 4.39 – 4.17 (m, 1H), 4.05 (ddd, *J* = 17.7, 13.7, 9.3 Hz, 1H), 3.45 (ddd, *J* = 13.8, 9.2, 4.8 Hz, 1H), 3.10 – 2.88 (m, 3H), 2.18 – 2.01 (m, 3H), 1.99 – 1.71 (m, 2H), 1.70 – 1.59 (m, 2H), 1.55 – 1.24 (m, 3H), 1.08 – 0.91 (m, 2H), 0.88 (t, *J* = 3.3 Hz, 5H), 0.84 – 0.78 (m, 1H), 0.76 – 0.61 (m, 3H), 0.56 (d, *J* = 7.0 Hz, 1H).

^{13}C NMR (101 MHz, $CDCl_3$) δ 172.2 (172.2), 172.1 (172.1), 146.9 (146.8), 129.2 (129.2), 118.1 (118.1), 113.2 (113.0), 75.7 (75.5), 56.7 (56.4), 50.1 (49.9), 46.9 (46.7), 40.7 (40.6), 37.5 (37.2), 34.1, 31.4 (31.4), 26.1 (25.6), 23.2 (22.9), 21.9, 21.8 (21.8), 20.8 (20.8), 16.0 (15.7).

HRMS (ESI-TOF) m/z: $[M + Na]^+$ calcd. for $C_{22}H_{34}N_2O_3Na$ 397.2467, found 397.2462.

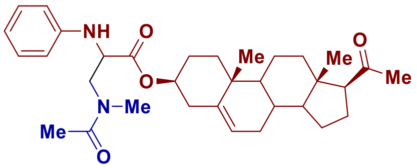

(3*S*,10*R*,17*S*)-17-acetyl-10-methyl-2,3,4,7,8,9,10,11,12,13,14,15,16,17-tetradecahydro-1H-cyclopenta[a]phenanthren-3-yl 3-(*N*-methylacetamido)-2-(phenylamino)propanoate (3v): Reaction using amide derivative *N*,*N*-dimethylacetamide (1.0 mL), and *N*-aryl glycine derivative **1v** (44.9 mg, 0.1 mmol, 1 equiv.), under standard condition. After being stirred for 24 hours, the reaction mixture was poured into 20 mL of water, and the resulting mixture was extracted with ethyl acetate (3 × 20 mL). The combined organic phase was dried over Na_2SO_4, filtered, and concentrated under vacuum. The residue was further purified by flash column, eluting with ethyl acetate:hexane (1:5) as white solid 21.3 mg, 40% yield (d.r, 1:1.5).

^{1}H NMR (400 MHz, $CDCl_3$) δ 7.19 – 7.08 (m, 2H), 6.79 – 6.64 (m, 1H), 6.64 – 6.54 (m, 2H), 5.40 – 5.28 (m, 1H), 4.72 – 4.50 (m, 2H), 4.34 – 4.19 (m, 1H), 4.03 – 3.88 (m, 1H), 3.54 – 3.43 (m, 1H), 3.05 – 2.87 (m, 9H), 2.55 – 2.45 (m, 1H), 2.34 – 2.25 (m, 1H), 2.09 (s, 3H), 2.06 – 2.03 (m, 5H), 1.88 – 1.70 (m, 3H), 1.62 – 1.52 (m, 3H), 1.47 – 1.40 (m, 2H), 1.24 – 1.09 (m, 3H), 1.00 – 0.95 (m, 3H), 0.60 (s, 2H).

^{13}C NMR (101 MHz, $CDCl_3$) δ 212.6 (209.4), 172.0, 171.8 (171.8), 170.6, 146.8 (146.8), 139.3, 129.2, 122.9 (122.6), 118.1 (118.1), 113.1 (113.1), 75.1 (75.1), 63.6 (61.2), 56.8 (56.2), 50.5, 50.1 (50.0), 49.8 (49.4), 45.4 (43.9), 38.7, 38.0 (37.9), 37.8 (37.8), 37.6, 37.5 (37.5), 36.9 (36.8), 36.6, 36.5 (36.5), 35.2 (35.0), 32.8, 31.9 (31.9), 31.8 (31.7), 31.5, 27.7 (27.5), 26.0, 24.5 (24.3), 22.8, 21.8 (21.6), 21.0, 20.9 (20.6), 19.3 (19.2), 13.2.

HRMS (ESI-TOF) m/z: $[M + H]^+$ calcd. for $C_{33}H_{47}N_2O_4$ 535.3536, found 535.3539.

6. Radical Trapping Studies:

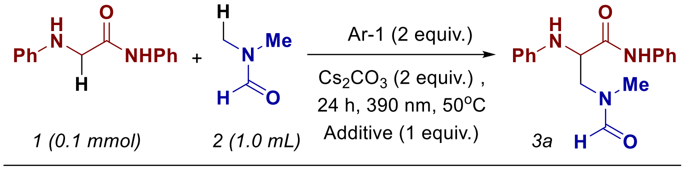

Additive	Yield of 3a
TEMPO	Trace
1,1-DPE	Trace
BHT	Trace

N-phenyl-2-(phenylamino) acetamide (**1**) (22.6 mg, 0.1 mmol, 1 equiv.), 4-iododbenzotrifluoride (**Ar-1**) (29.5 µL, 0.2 mmol, 2 equiv.), Cs_2CO_3 (65.0 mg, 0.2 mmol, 2.0 equiv.), additive (0.1 mmol, 1 equiv.) and *N*,*N*-dimethylformamide (1.0 mL) were mixed in a 10 mL microwave vial equipped with a stir bar under argon atmosphere. The vial was sealed with a septum-cap and placed 3 cm away from two 390 nm blue LED (40W). The temperature was kept at approximately 50°C (heating caused by LED lamp). After being stirred for 24 hours, the reaction mixture was poured into 20 mL of water, and the resulting mixture was extracted with ethyl acetate (3 × 20 mL). The combined organic phase was dried over Na_2SO_4, filtered, and concentrated under vacuum. Aliquots were collected for GC-MS and HR-MS analysis, and the crude yields were obtained using ^{1}HNMR analysis using dibromomethane as internal standard.

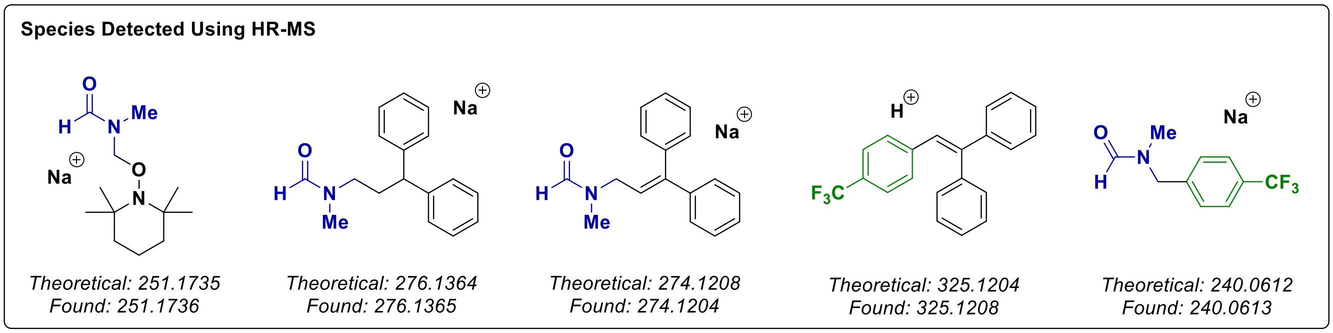

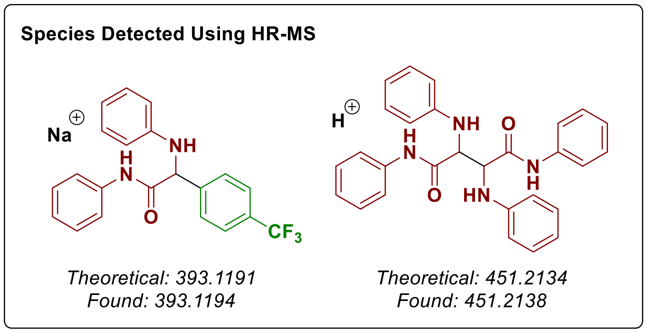

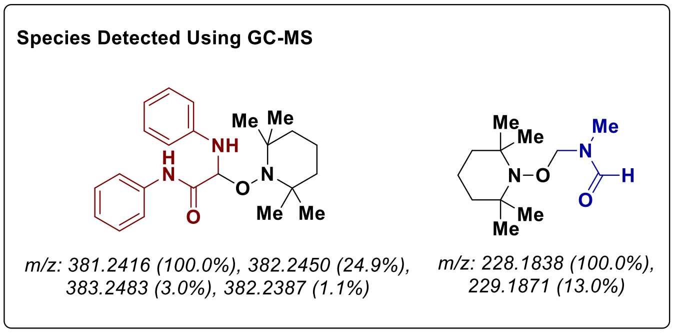

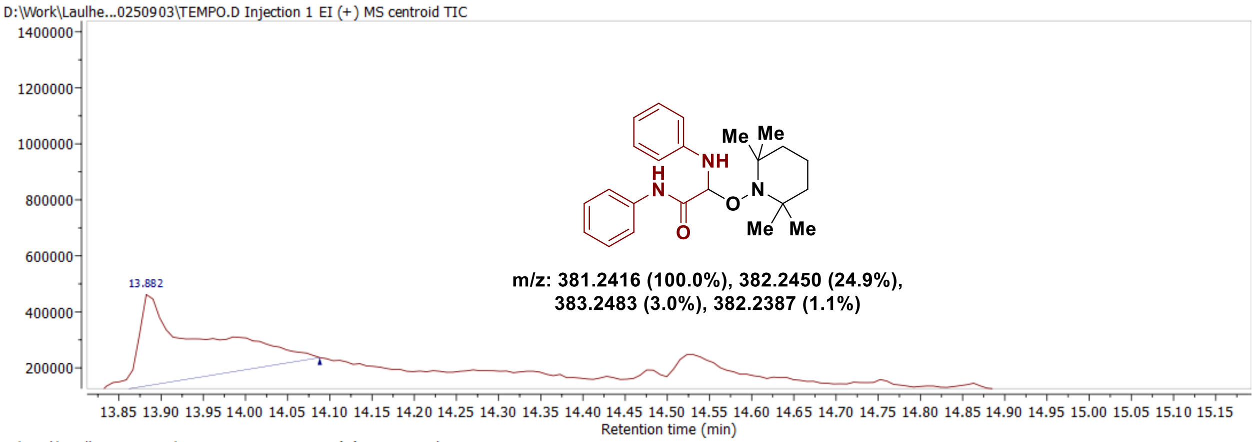
D:\Work\Laulhe...0250903\TEMPO.D Injection 1 EI (+) MS centroid TIC
13.882
Me Me
NH
H
N
O
O
N
Me Me
m/z: 381.2416 (100.0%), 382.2450 (24.9%), 383.2483 (3.0%), 382.2387 (1.1%)
Retention time (min)

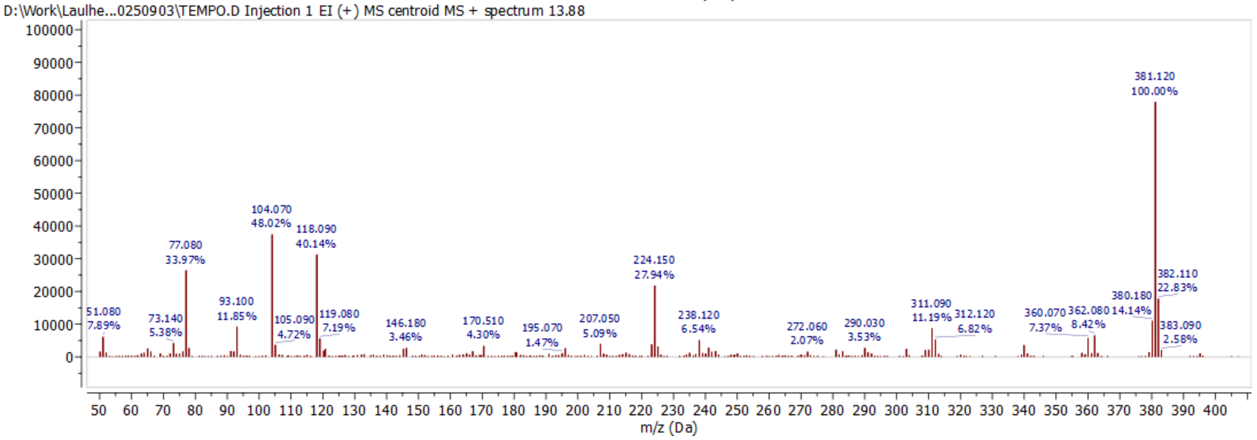
D:\Work\Laulhe...0250903\TEMPO.D Injection 1 EI (+) MS centroid MS + spectrum 13.88
381.120 100.00%
104.070 48.02%
118.090 40.14%
77.080 33.97%
224.150 27.94%
382.110 22.83%
380.180 14.14%
93.100 11.85%
311.090 11.19%
362.080 8.42%
51.080 7.89%
360.070 7.37%
119.080 7.19%
312.120 6.82%
238.120 6.54%
73.140 5.38%
207.050 5.09%
105.090 4.72%
170.510 4.30%
290.030 3.53%
146.180 3.46%
383.090 2.58%
272.060 2.07%
195.070 1.47%
m/z (Da)

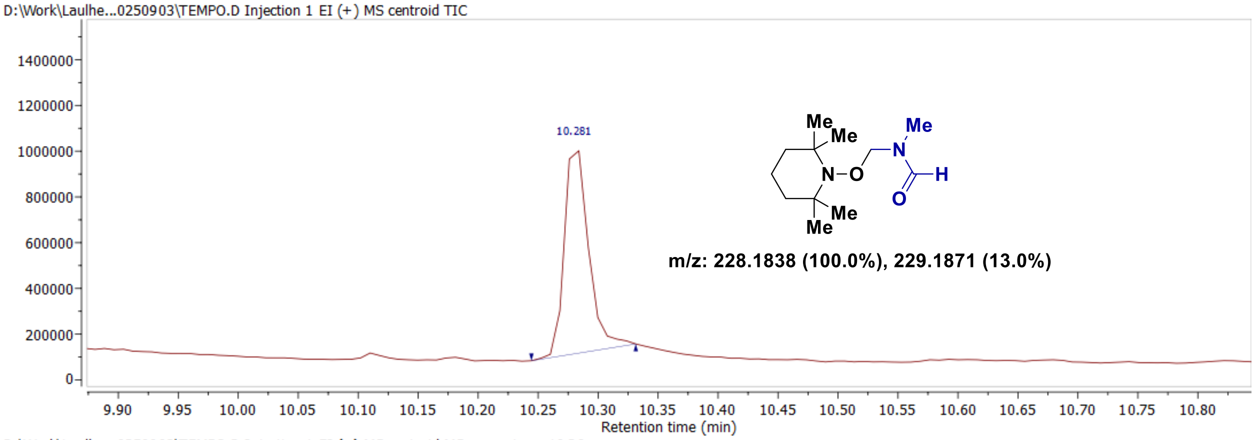
D:\Work\Laulhe...0250903\TEMPO.D Injection 1 EI (+) MS centroid TIC
10.281
Me Me
N–O
Me Me
Me
N
H
O
m/z: 228.1838 (100.0%), 229.1871 (13.0%)
Retention time (min)

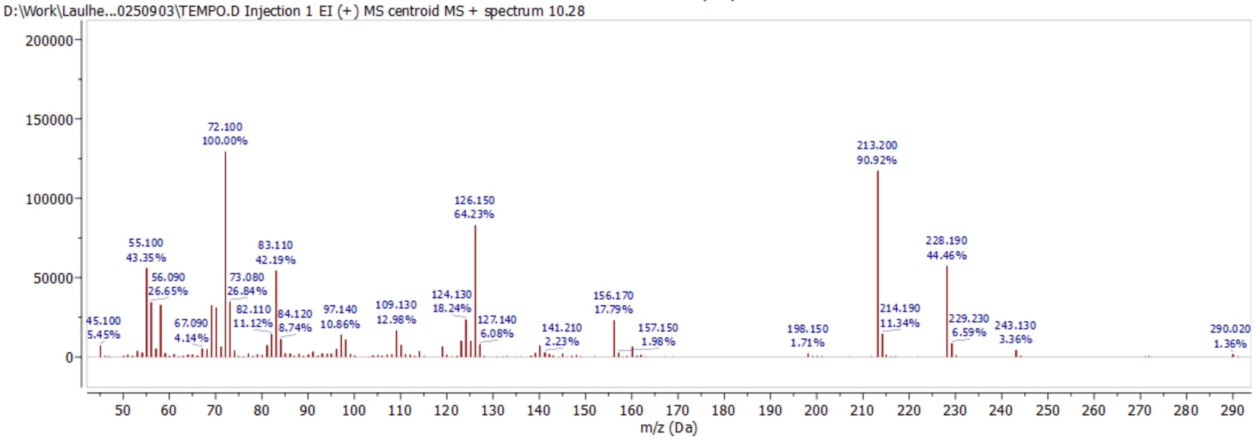
D:\Work\Laulhe...0250903\TEMPO.D Injection 1 EI (+) MS centroid MS + spectrum 10.28
72.100 100.00%
213.200 90.92%
126.150 64.23%
228.190 44.46%
55.100 43.35%
83.110 42.19%
73.080 26.84%
56.090 26.65%
124.130 18.24%
156.170 17.79%
109.130 12.98%
214.190 11.34%
82.110 11.12%
97.140 10.86%
84.120 8.74%
229.230 6.59%
127.140 6.08%
45.100 5.45%
67.090 4.14%
243.130 3.36%
141.210 2.23%
157.150 1.98%
198.150 1.71%
290.020 1.36%
m/z (Da)

7. UV-Vis Studies:

Preparation of Stock Solutions:

1. **Cs_2CO_3**: 0.3 mmol/mL in DMF.
2. *N*-phenyl-2-(phenylamino) acetamide (**Ph-glycine**): 0.2 mmol/mL in DMF.
3. 4-iodobenzotrifluoride (**Ar-1**): 0.4 mmol/mL in DMF.
4. **Ph-glycine** + **Cs_2CO_3**: 0.2 mmol/mL of **Ph-glycine** and 0.3 mmol/mL of **Cs_2CO_3** in DMF.
5. **Ar-1** + **Cs_2CO_3**: 0.4 mmol/mL of **Ar-1** and 0.3 mmol/mL of **Cs_2CO_3** in DMF.

UV-Vis Spectra Experiments:

- Final concentrations: 0.1 mmol/mL of **Ph-glycine**, 0.2 mmol/mL of **Ar-1**, and 0.15 mmol/mL of **Cs_2CO_3**.

Experiments:

- Experiment A: 2 mL DMF.
- Experiment B: 1 mL of stock 1, diluted to 2 mL in DMF.
- Experiment C: 1 mL of stock 1, diluted to 2 mL in DMF.
- Experiment D: 1 mL of stock 2, diluted to 2 mL in DMF.
- Experiment E: 1 mL of stock 3, diluted to 2 mL in DMF.
- Experiment F: 1 mL of stock 4, diluted to 2 mL in DMF.
- Experiment G: 1 mL of stock 1 and 1 mL of stock 2.
- Experiment H: 2 mL of stock 1 and 2 mL of stock 2, combined with Cs_2CO_3 (0.15 mmol/mL), and 2 mL used in the uv-vis cuvette.

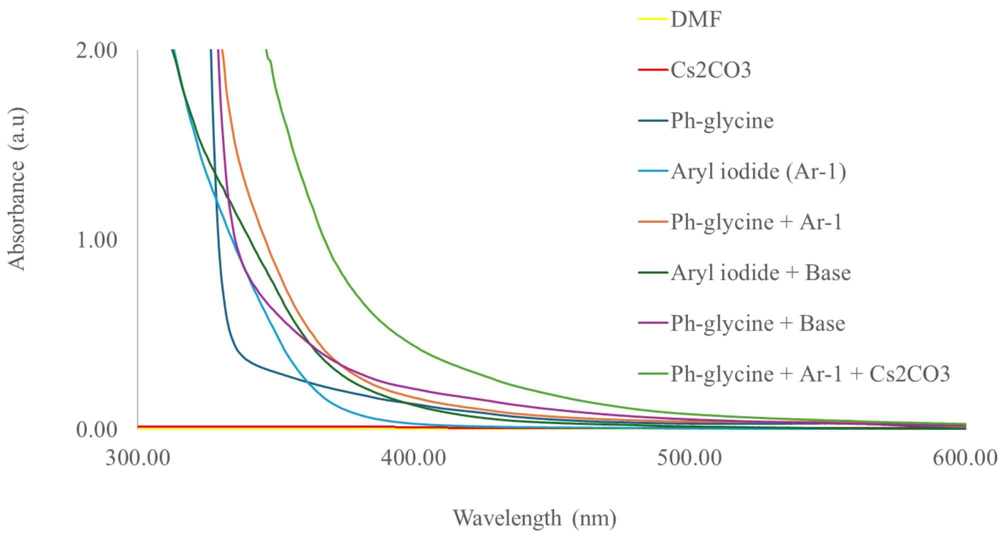

The observation of a charge-transfer band upon mixing N-phenyl-2-(phenylamino) acetamide with 4-iodobenzotrifluoride and Cs_2CO_3 indicates the potential formation of a photoactive aggregate.

8. Miscellaneous Studies:

8.1

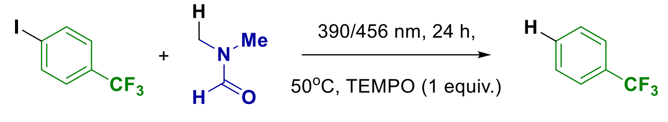

Ar-1 (0.2 mmol) *2 (1 mL)* *Ar-1a, Trace*

8.2

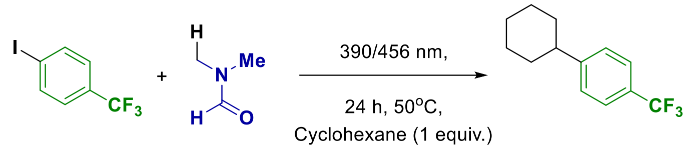

Ar-1 (0.2 mmol) *2 (1 mL)* *Ar-1b, 0%*

9. ^{1}H NMR, ^{19}F NMR, ^{13}C NMR Spectra:

^{1}H NMR (400 MHz, $CDCl_3$)

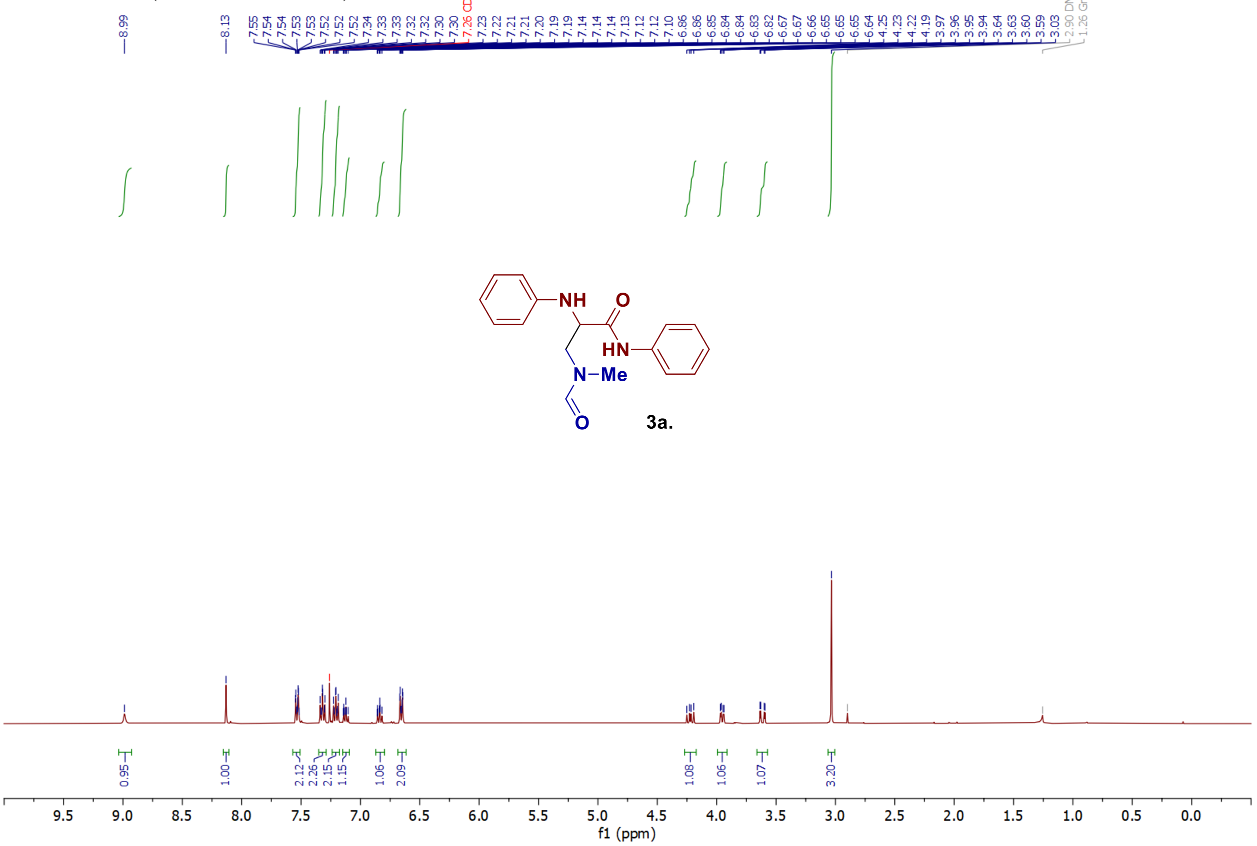

^{13}C NMR (101 MHz, $CDCl_3$)

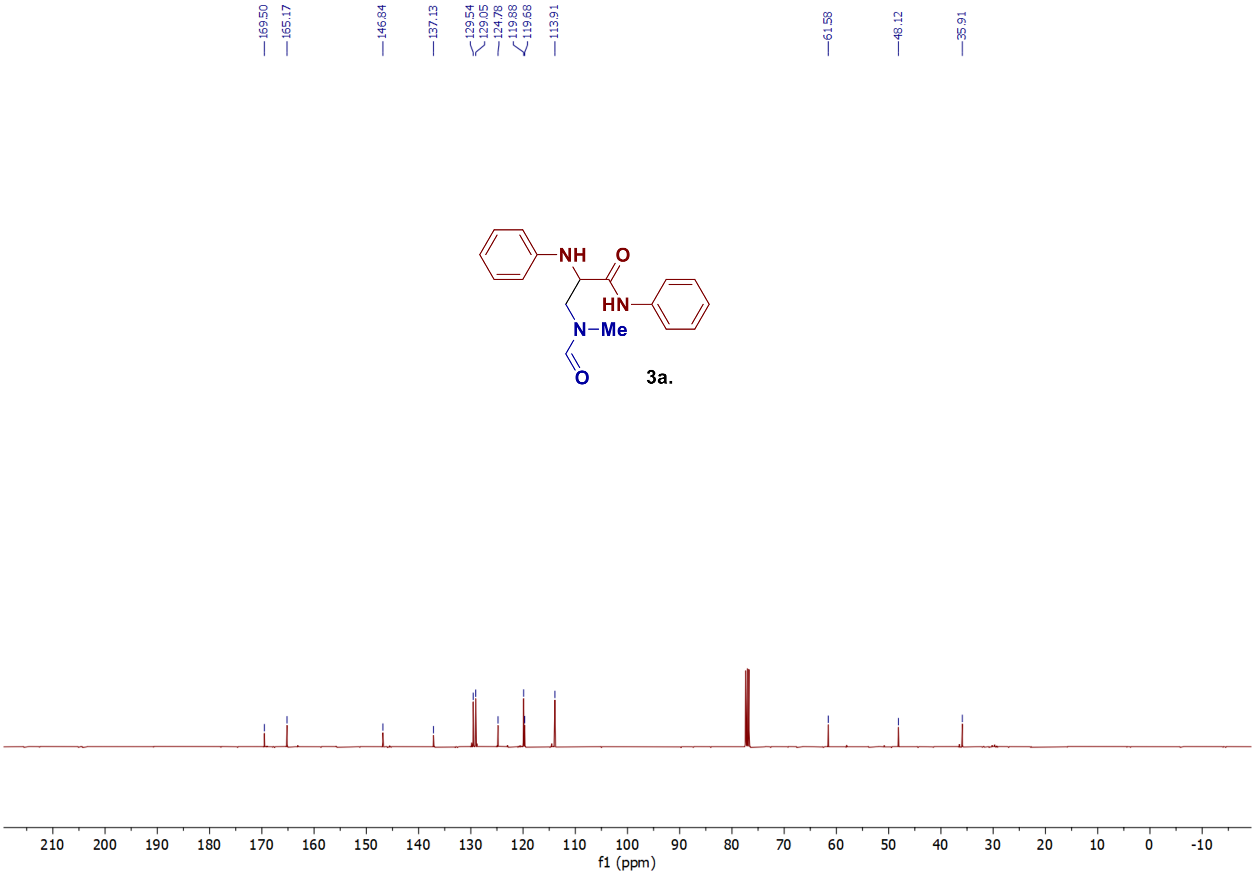

^{1}H NMR (400 MHz, $CDCl_3$)

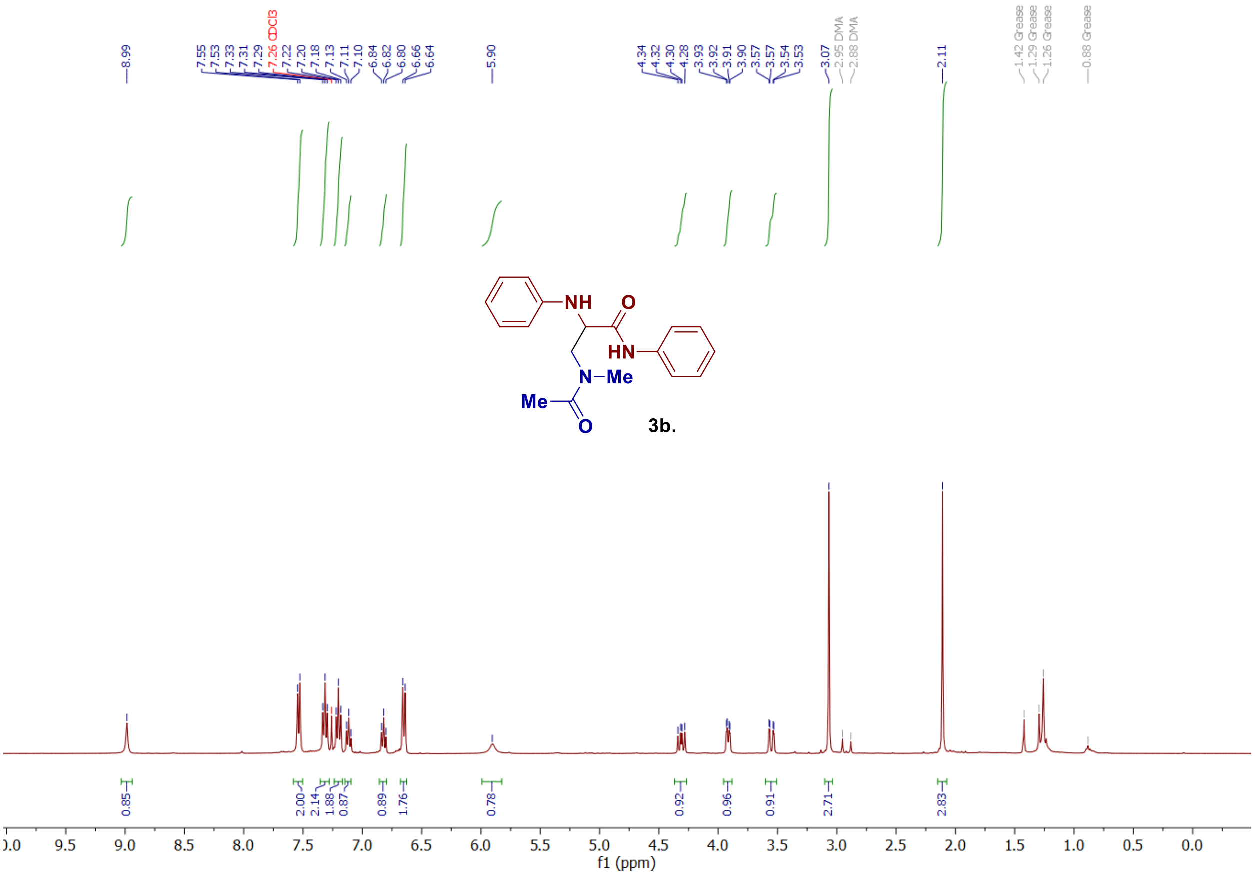

^{13}C NMR (101 MHz, $CDCl_3$)

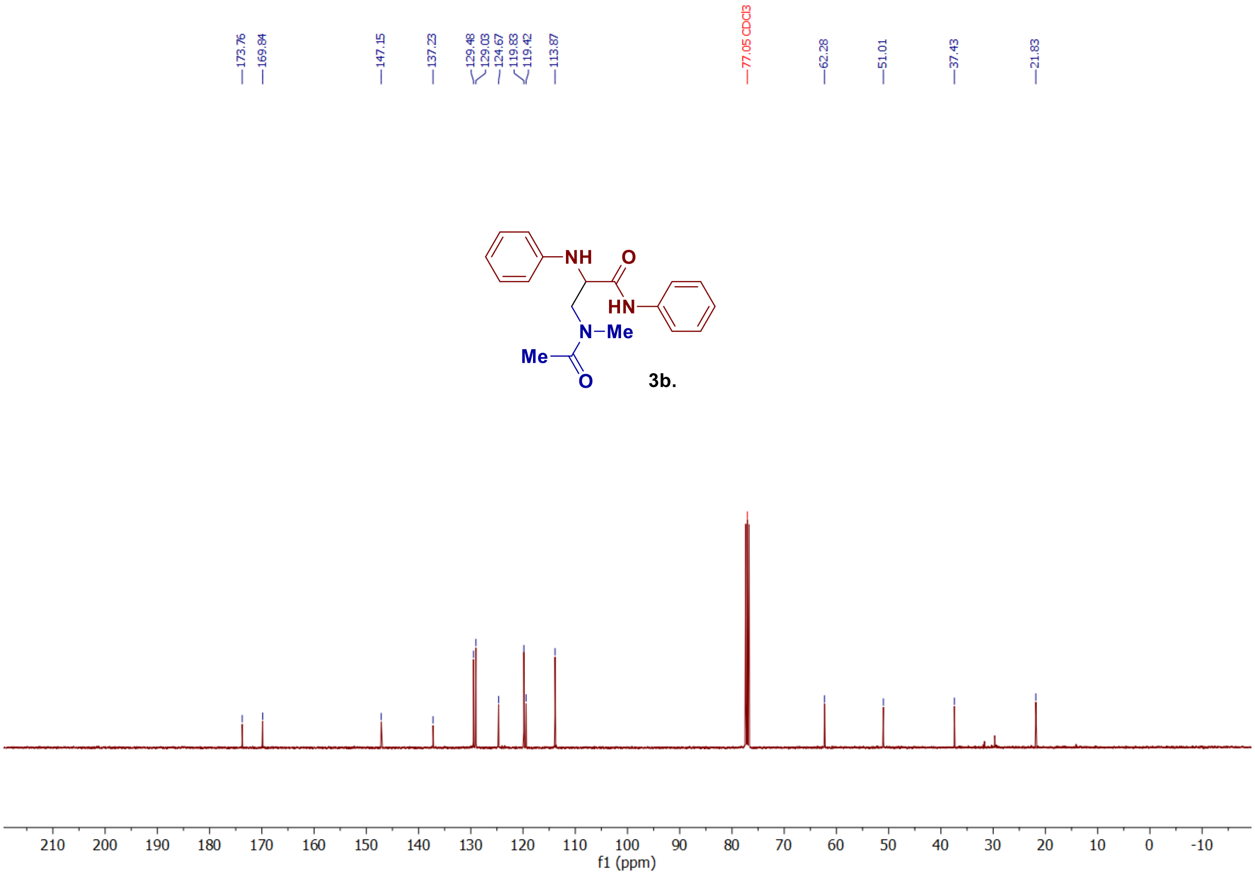

^{1}H NMR (400 MHz, $CDCl_3$)

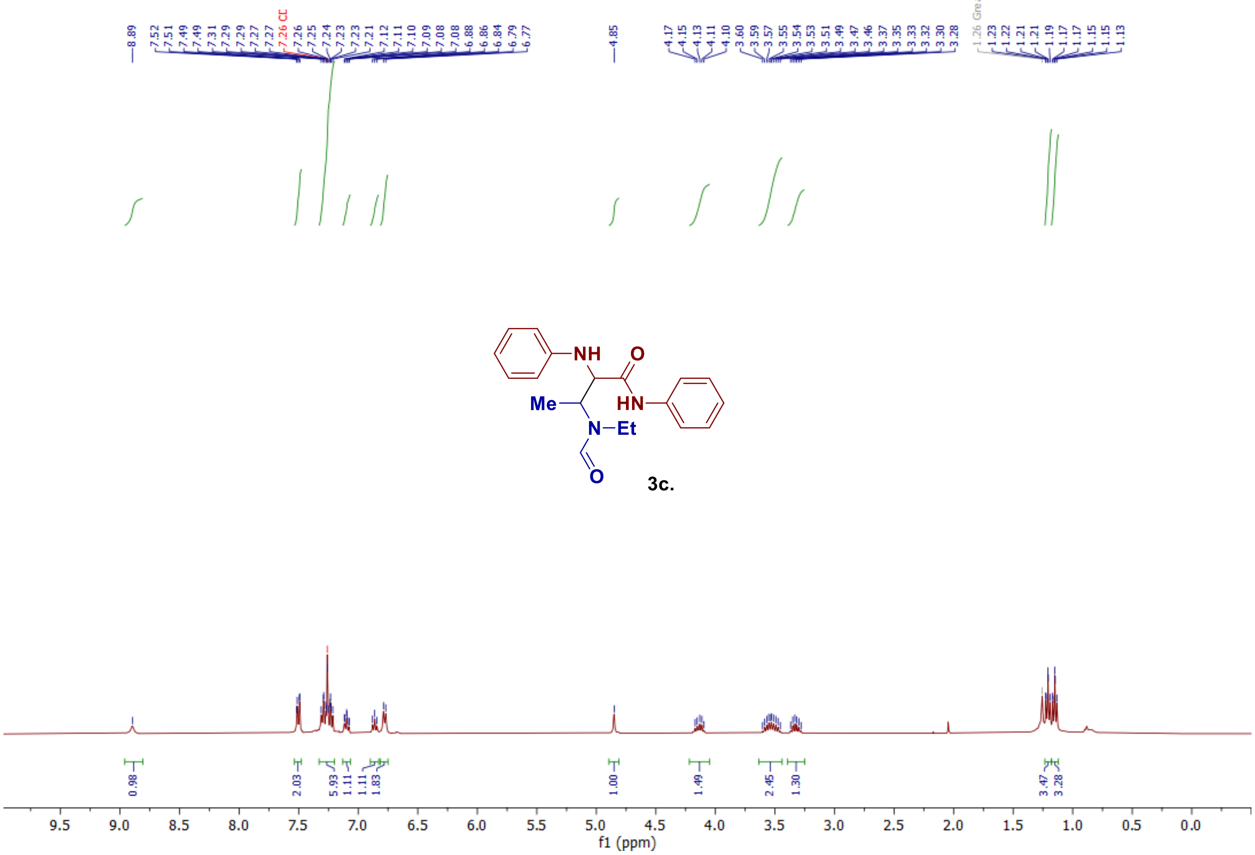

^{13}C NMR (101 MHz, $CDCl_3$)

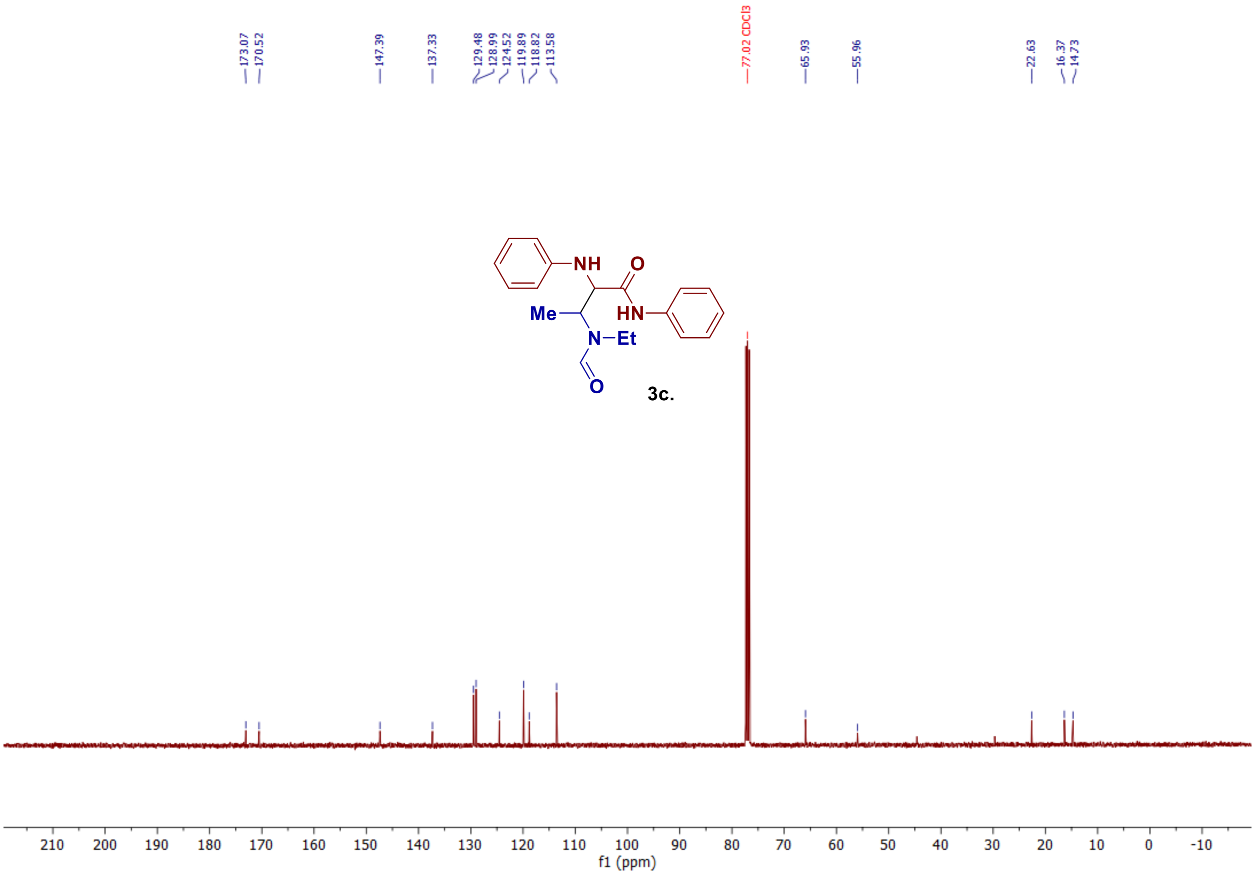

^{1}H NMR (400 MHz, $CDCl_3$)

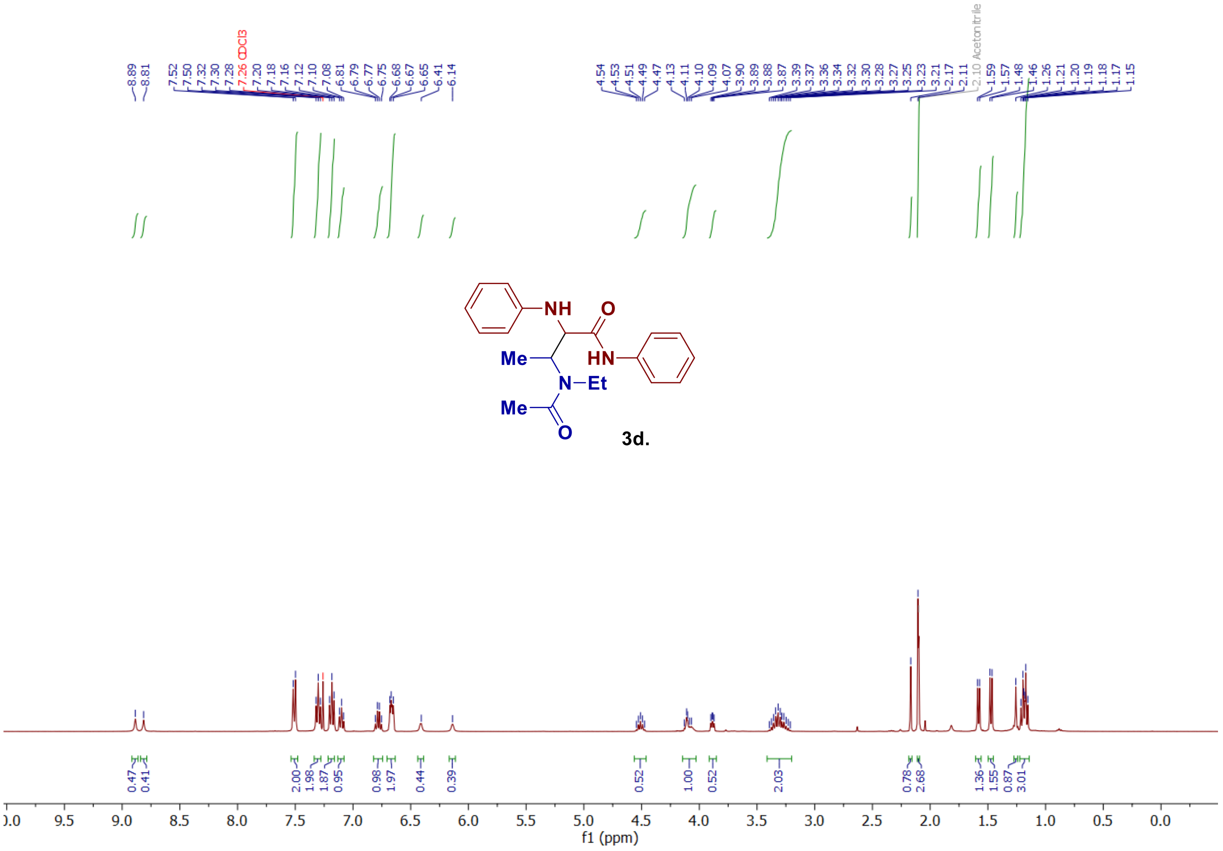

^{13}C NMR (101 MHz, $CDCl_3$)

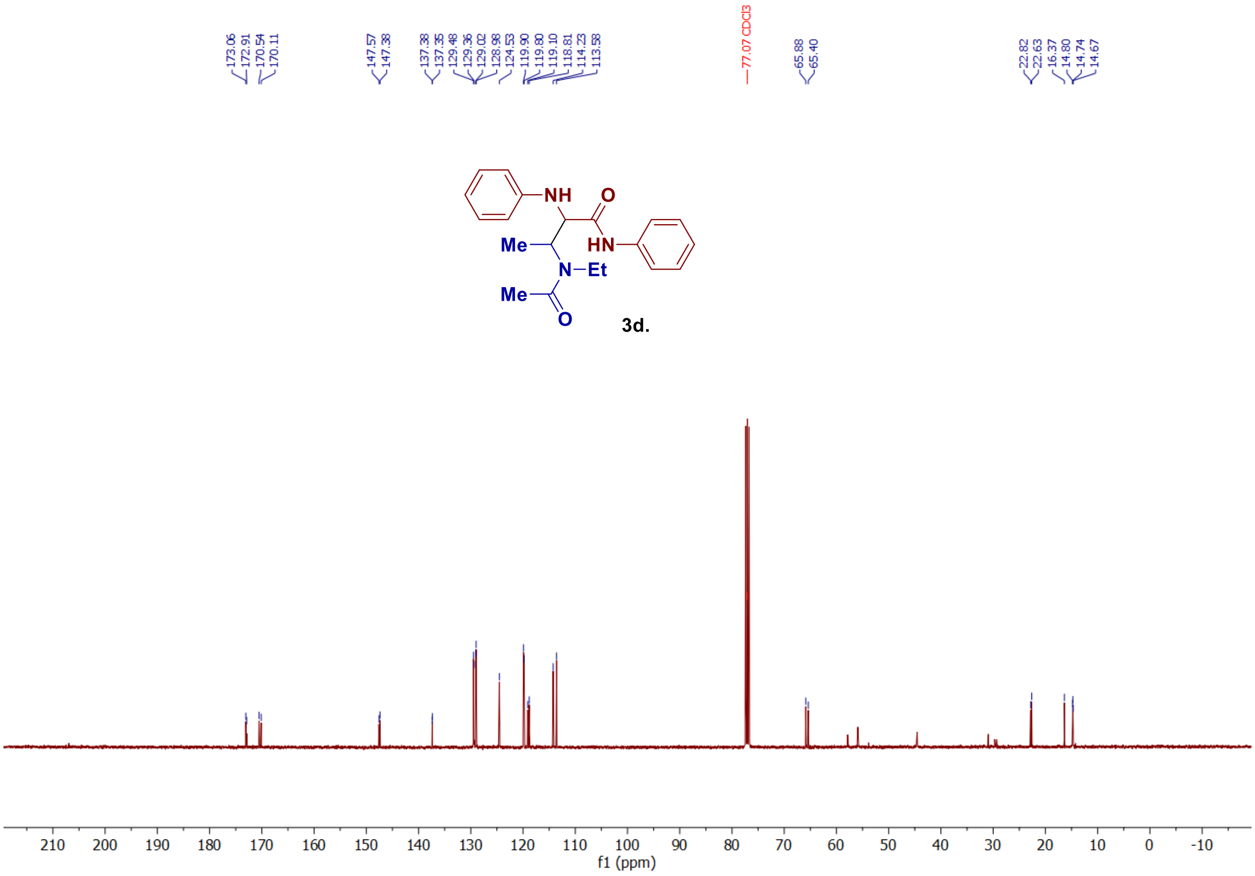

^{1}H NMR (400 MHz, $CDCl_3$)

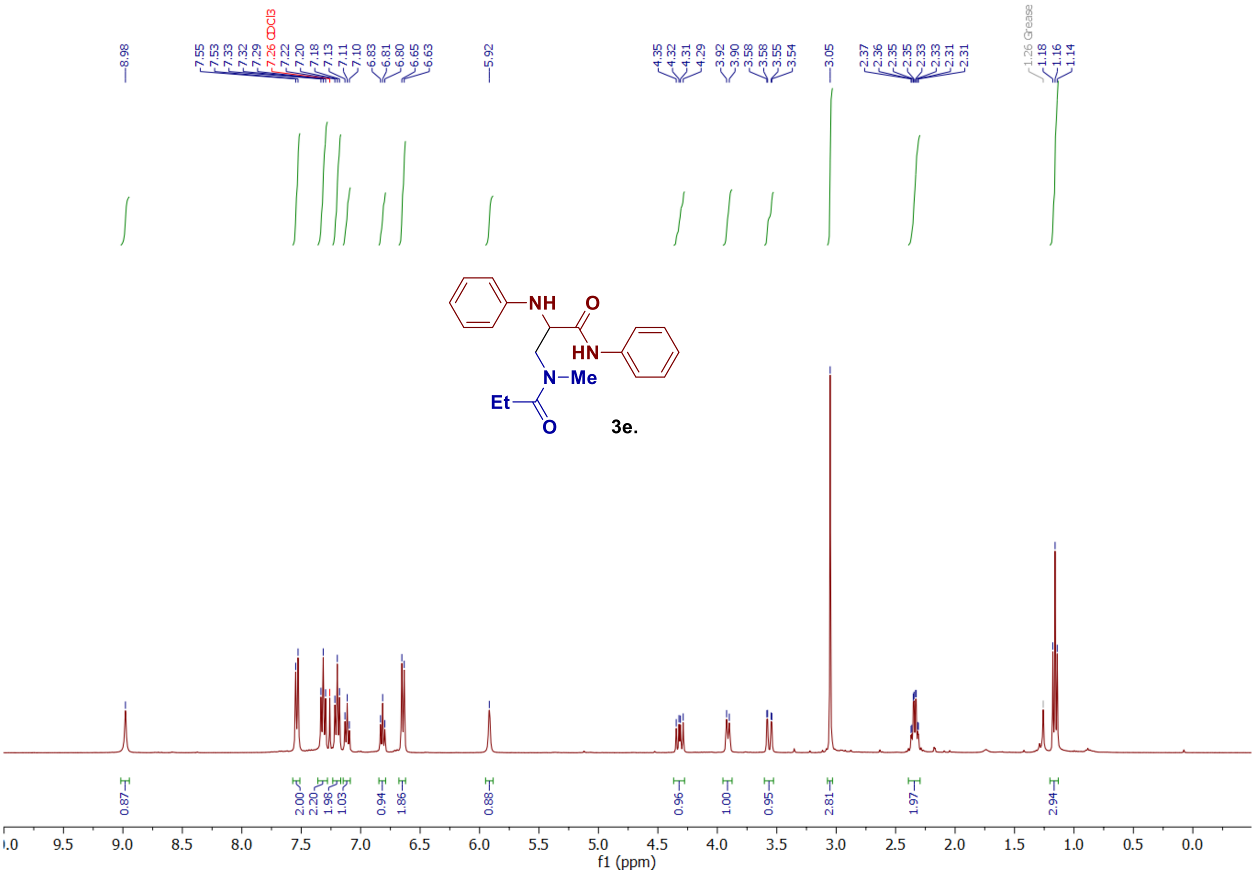

^{13}C NMR (101 MHz, $CDCl_3$)

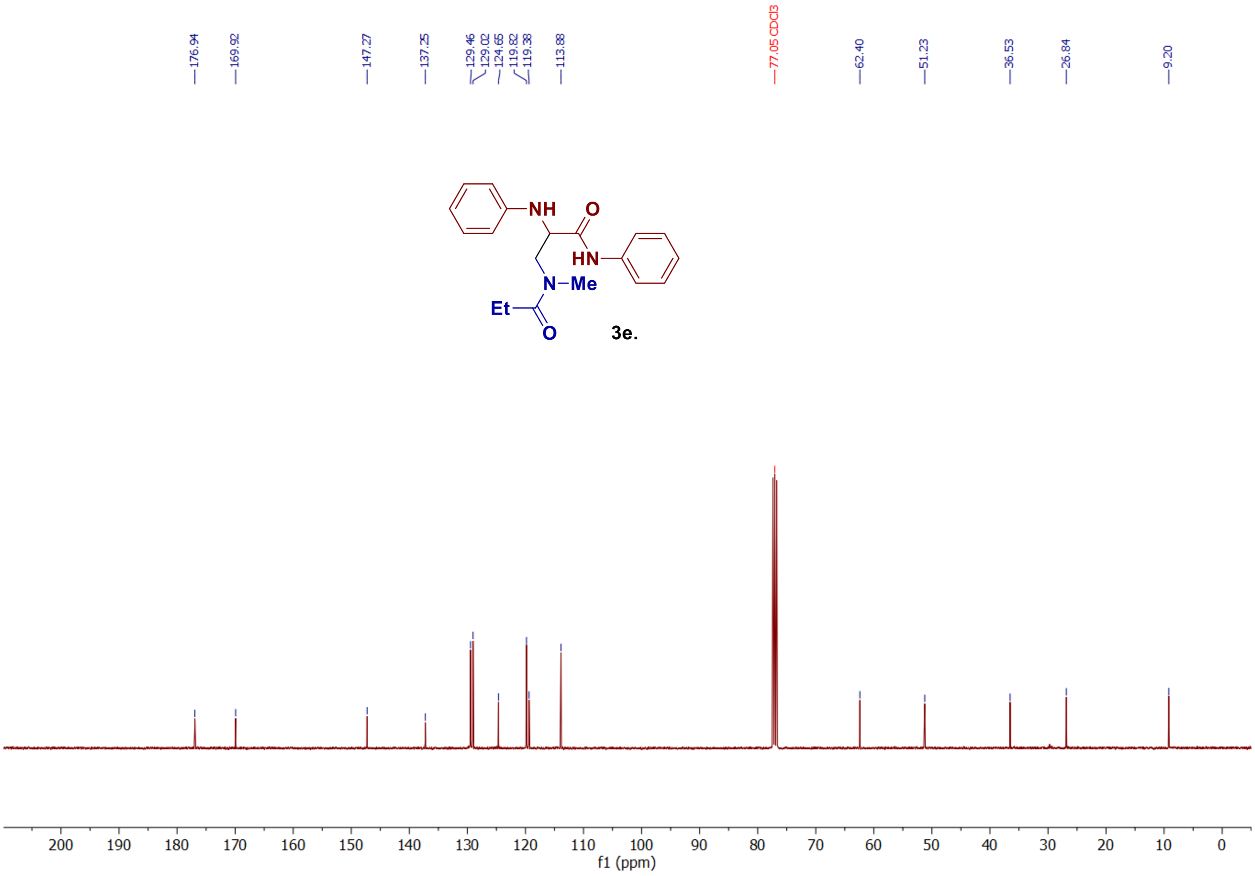

^{1}H NMR (400 MHz, $CDCl_3$)

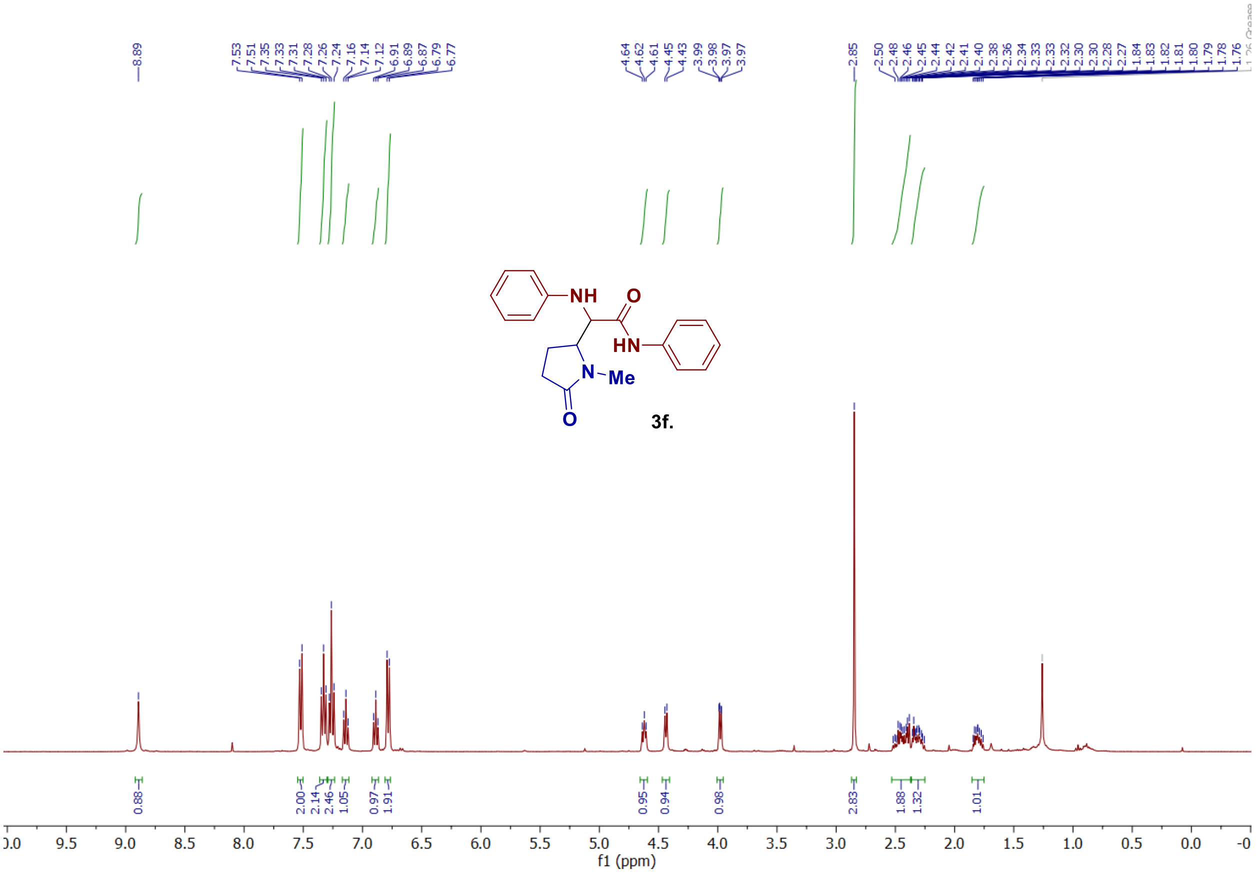

^{13}C NMR (101 MHz, $CDCl_3$)

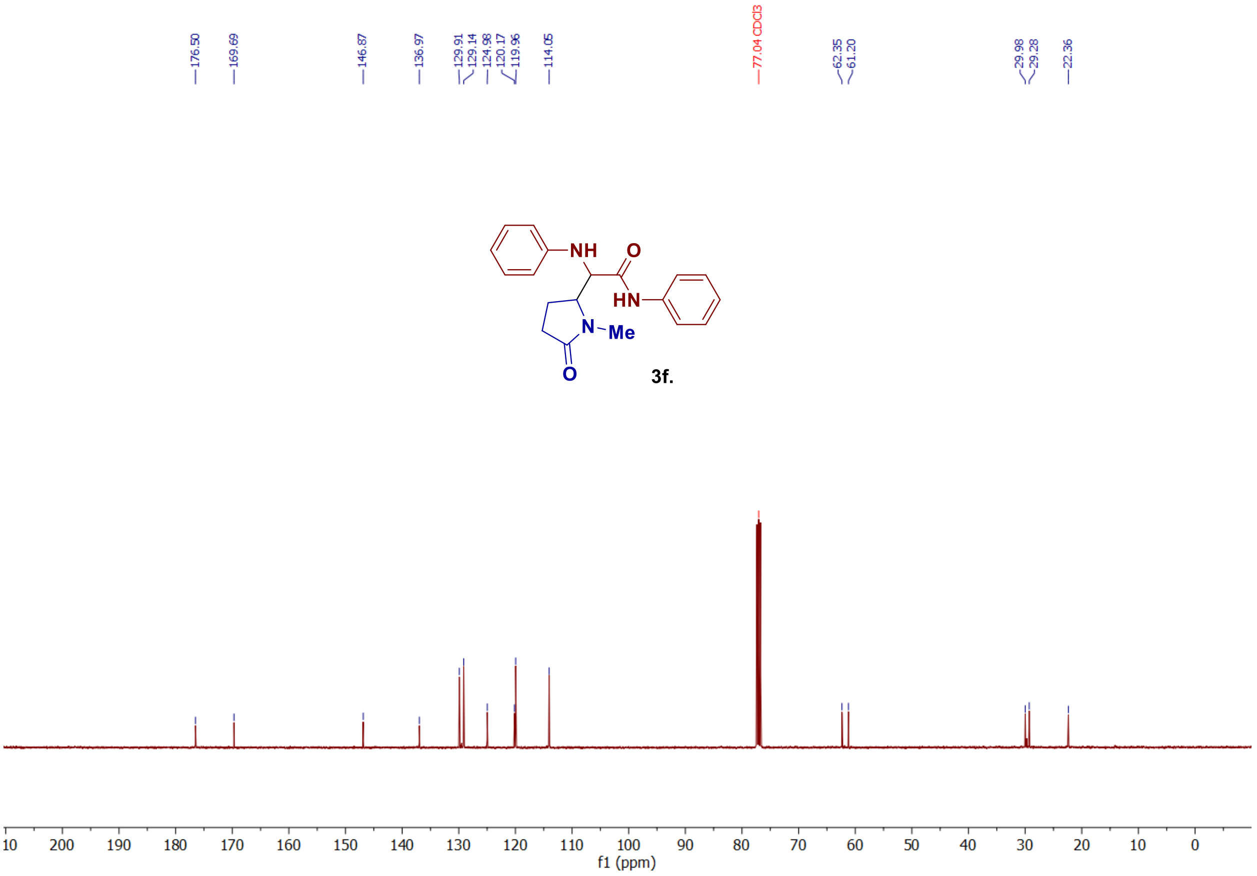

[1]**H NMR** (400 MHz, $CDCl_3$)

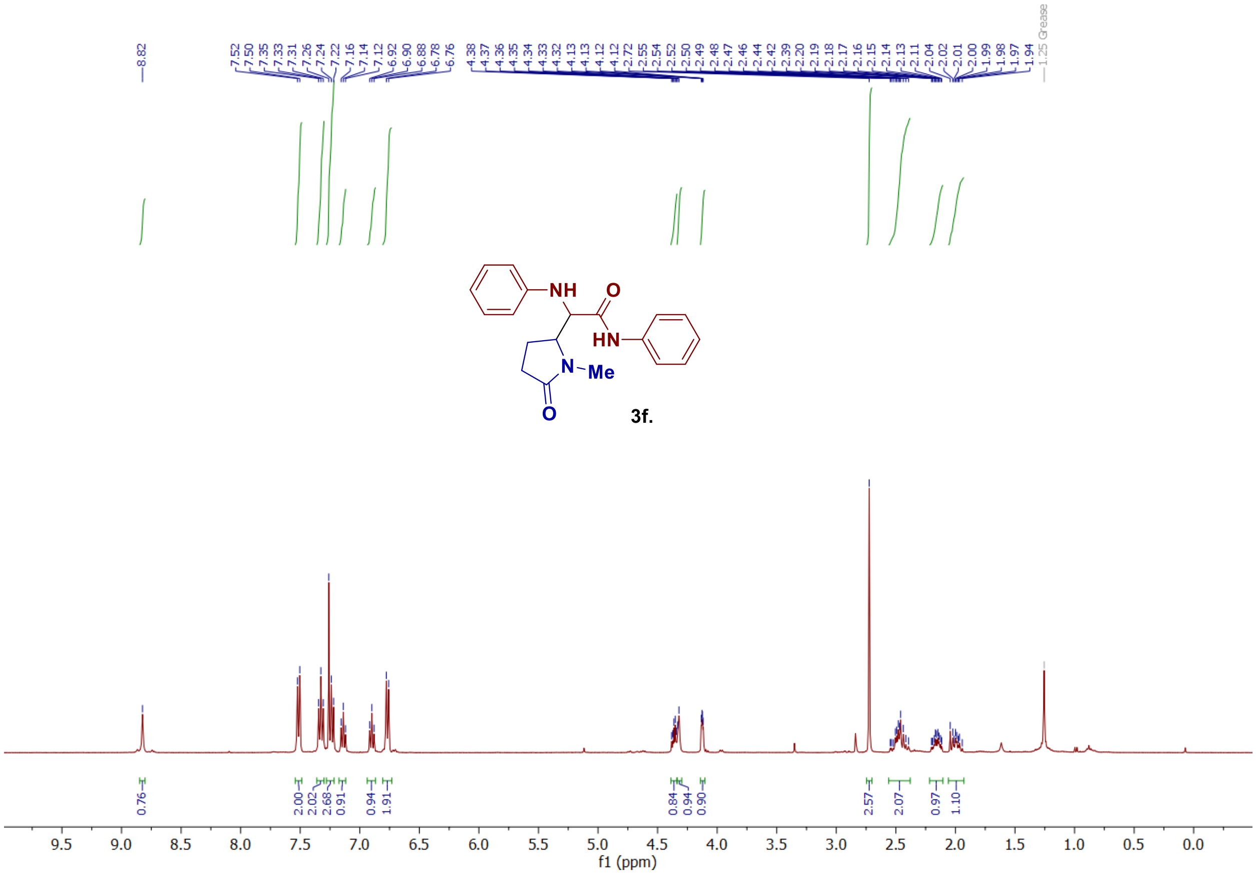

[13]**C NMR** (101 MHz, $CDCl_3$)

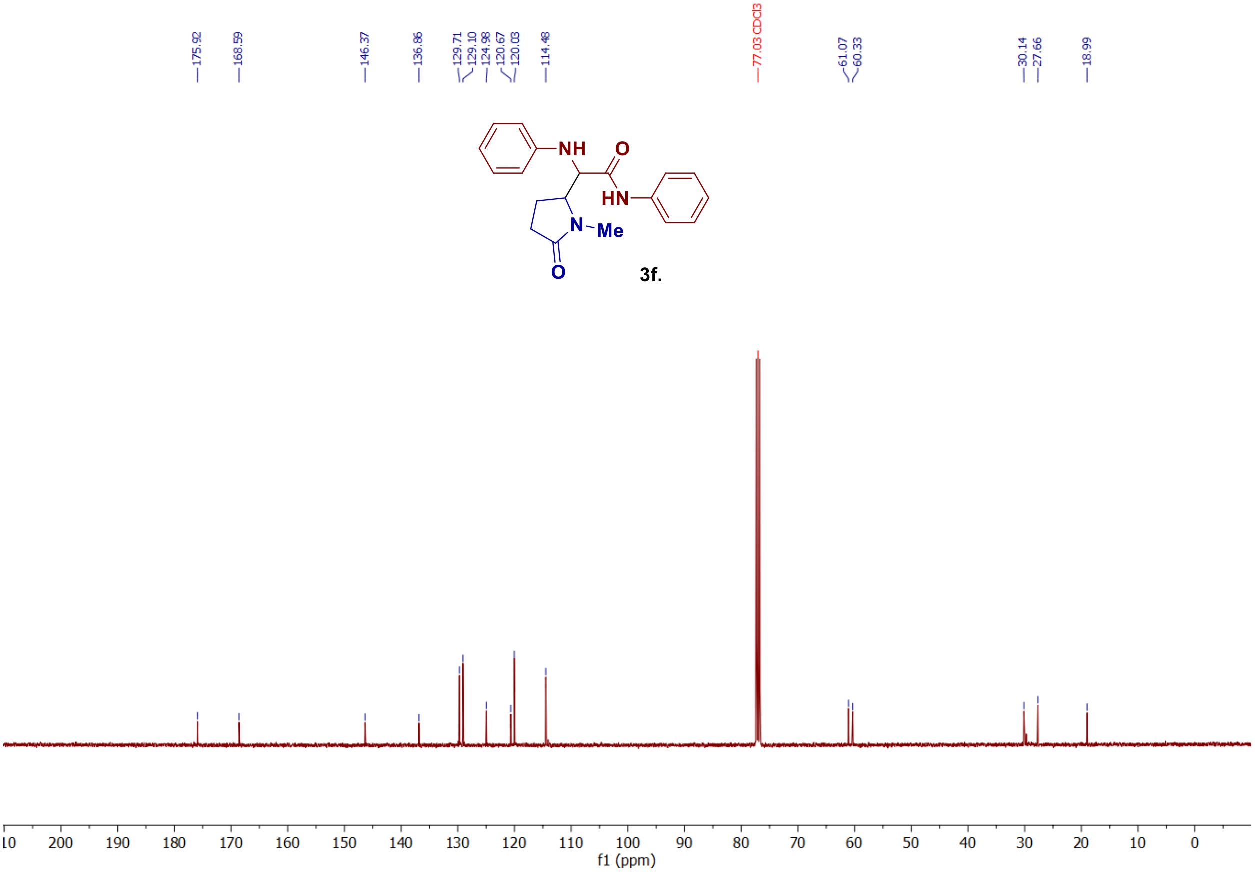

^{1}H NMR (400 MHz, $CDCl_3$)

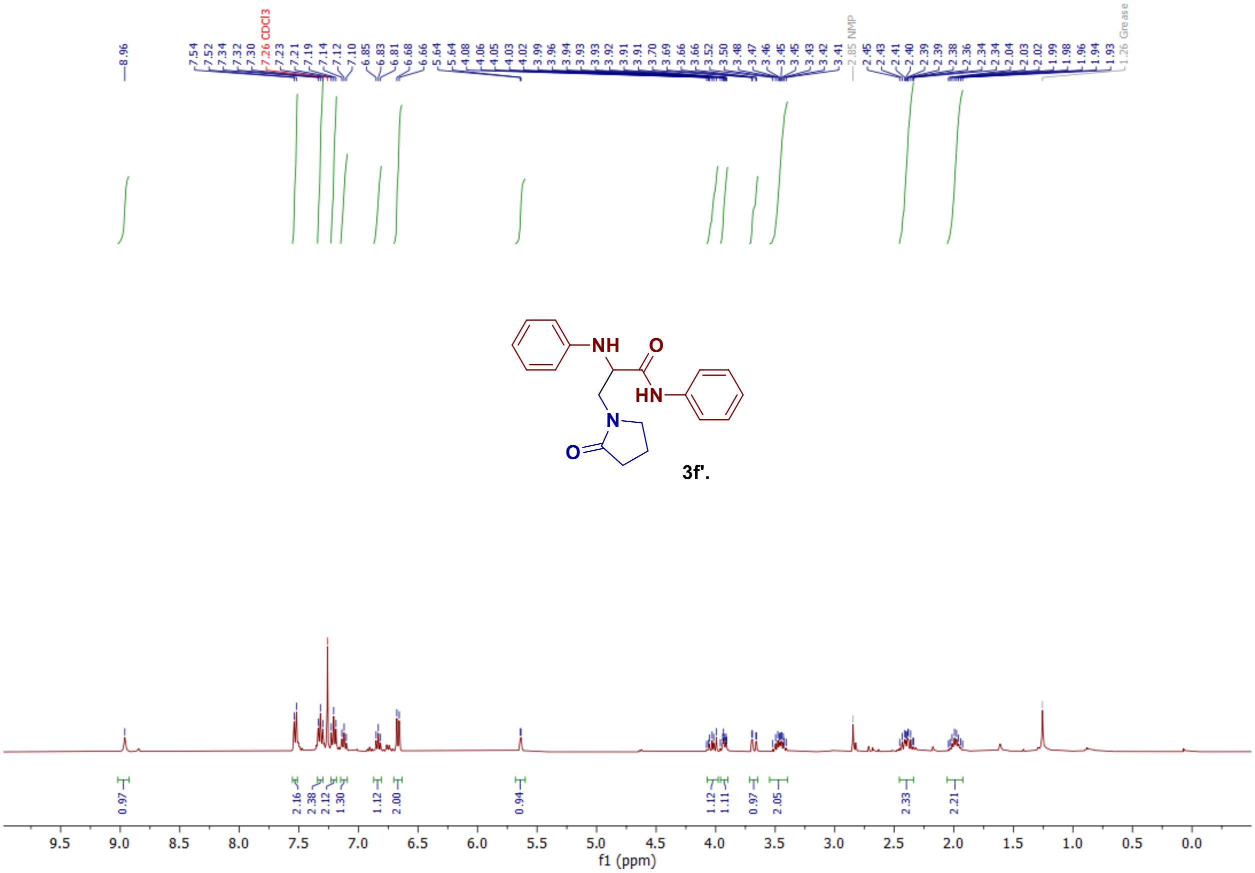

3f'.

^{1}H NMR (400 MHz, $CDCl_3$)

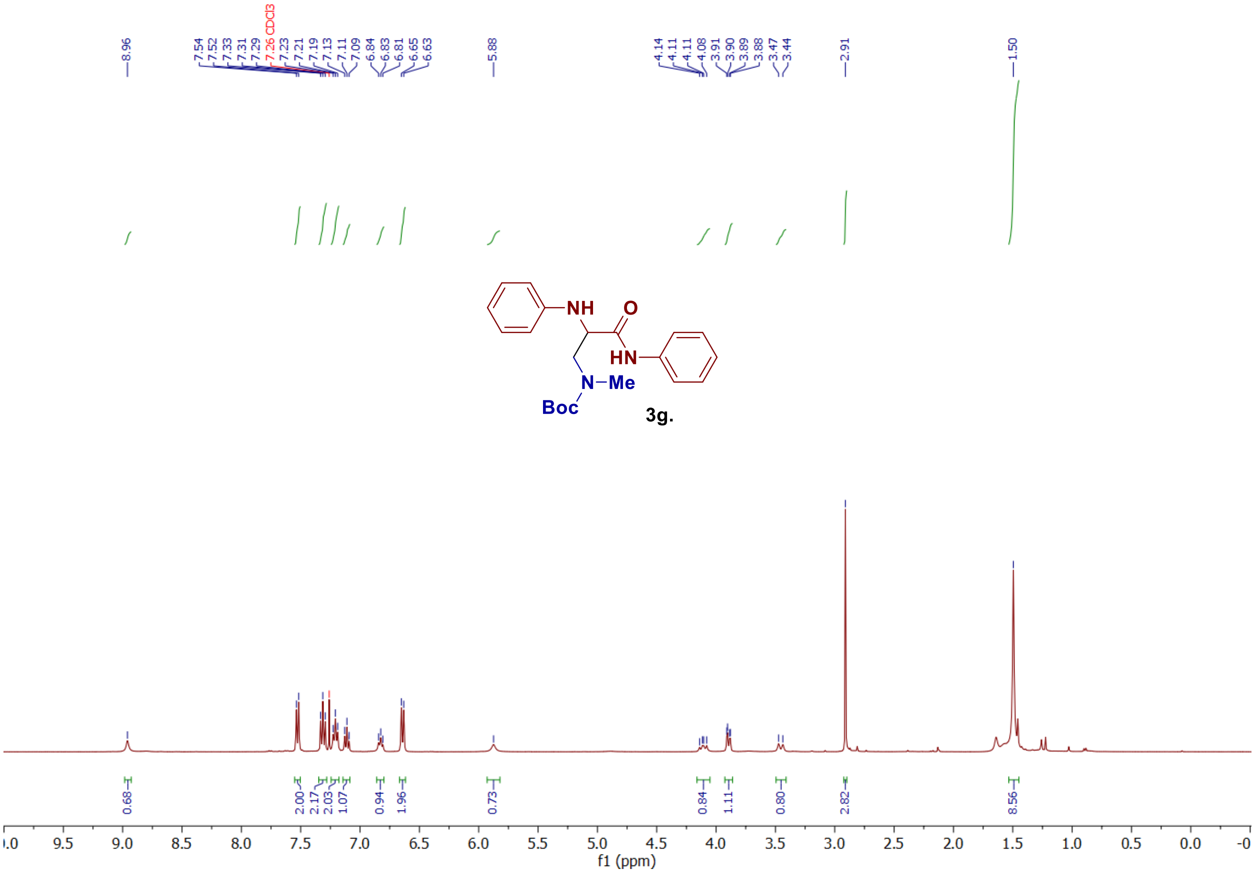

^{13}C NMR (101 MHz, $CDCl_3$)

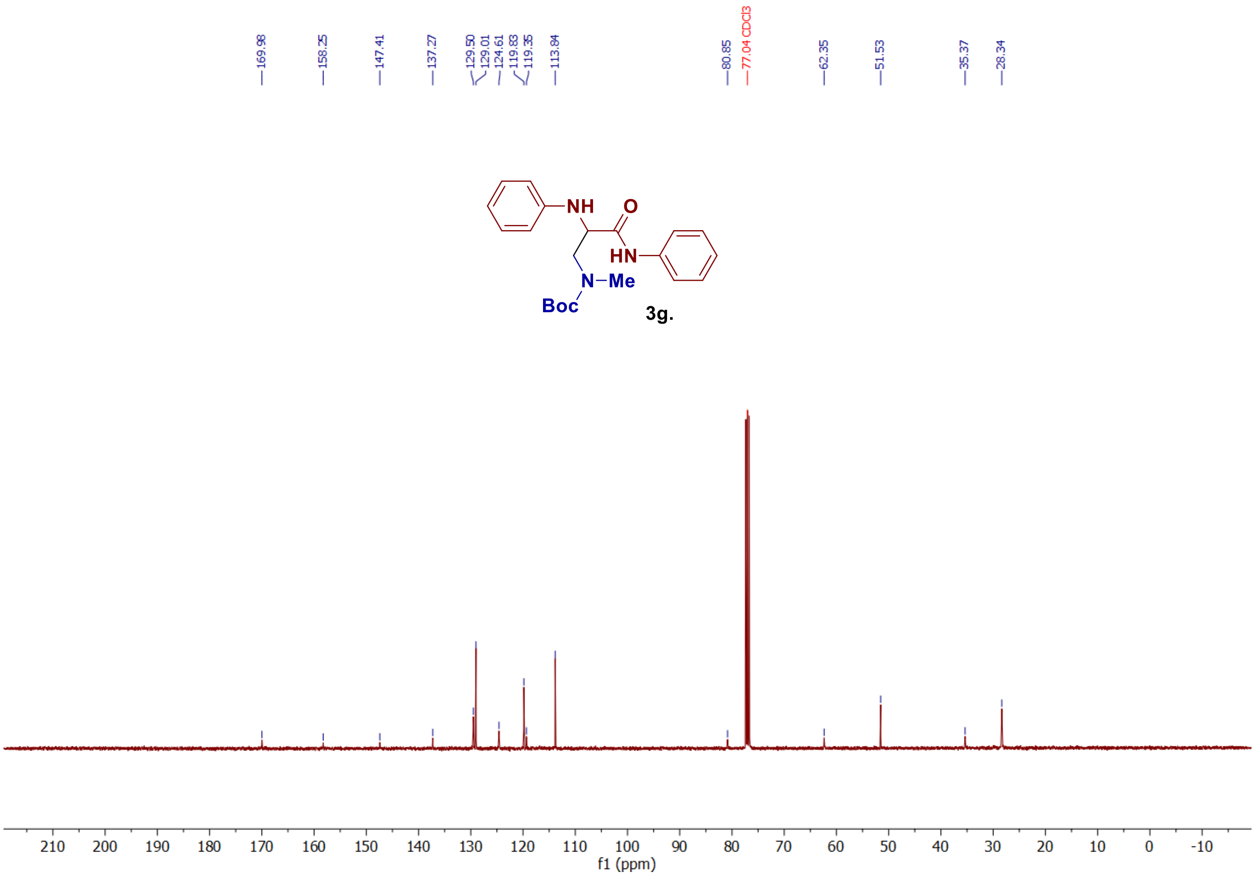

^{1}H NMR (400 MHz, $CDCl_3$)

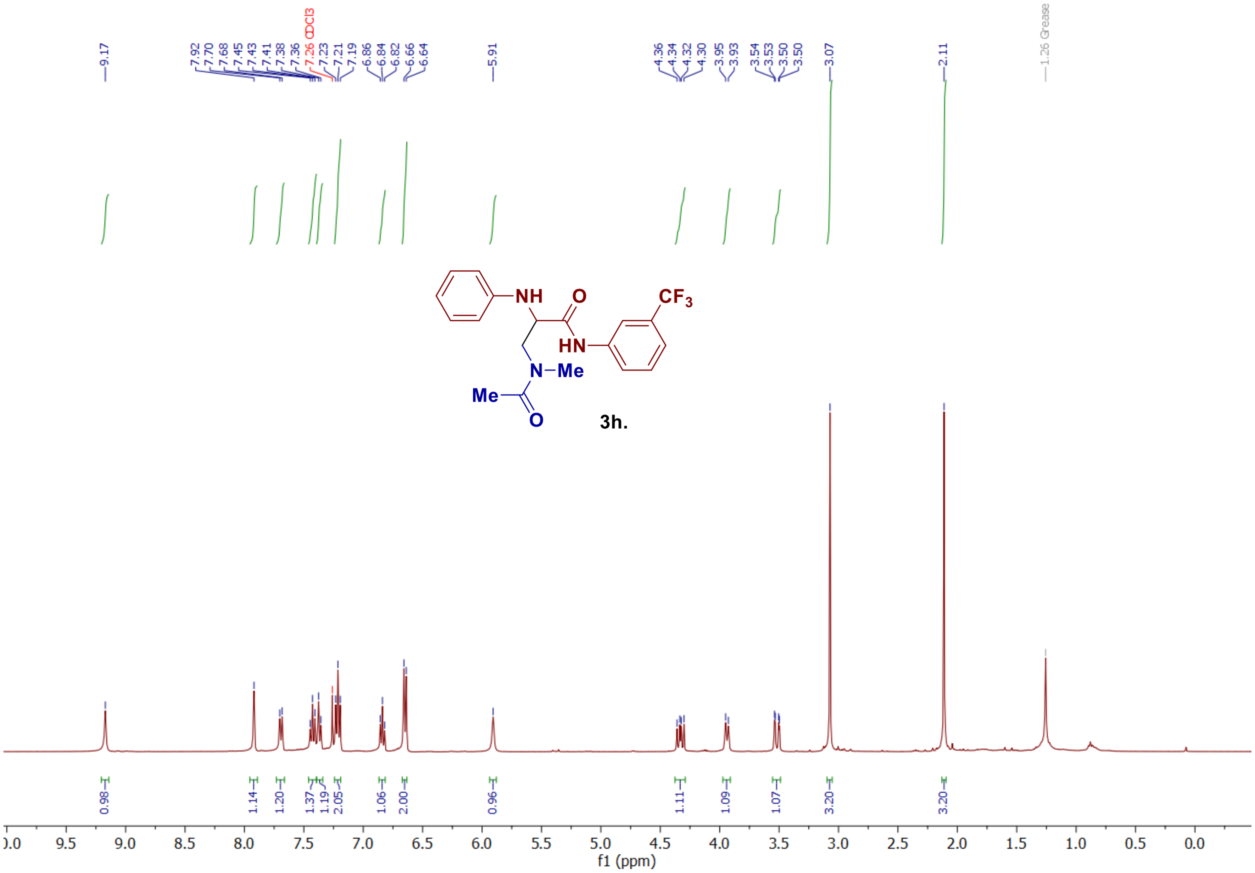

^{13}C NMR (101 MHz, $CDCl_3$)

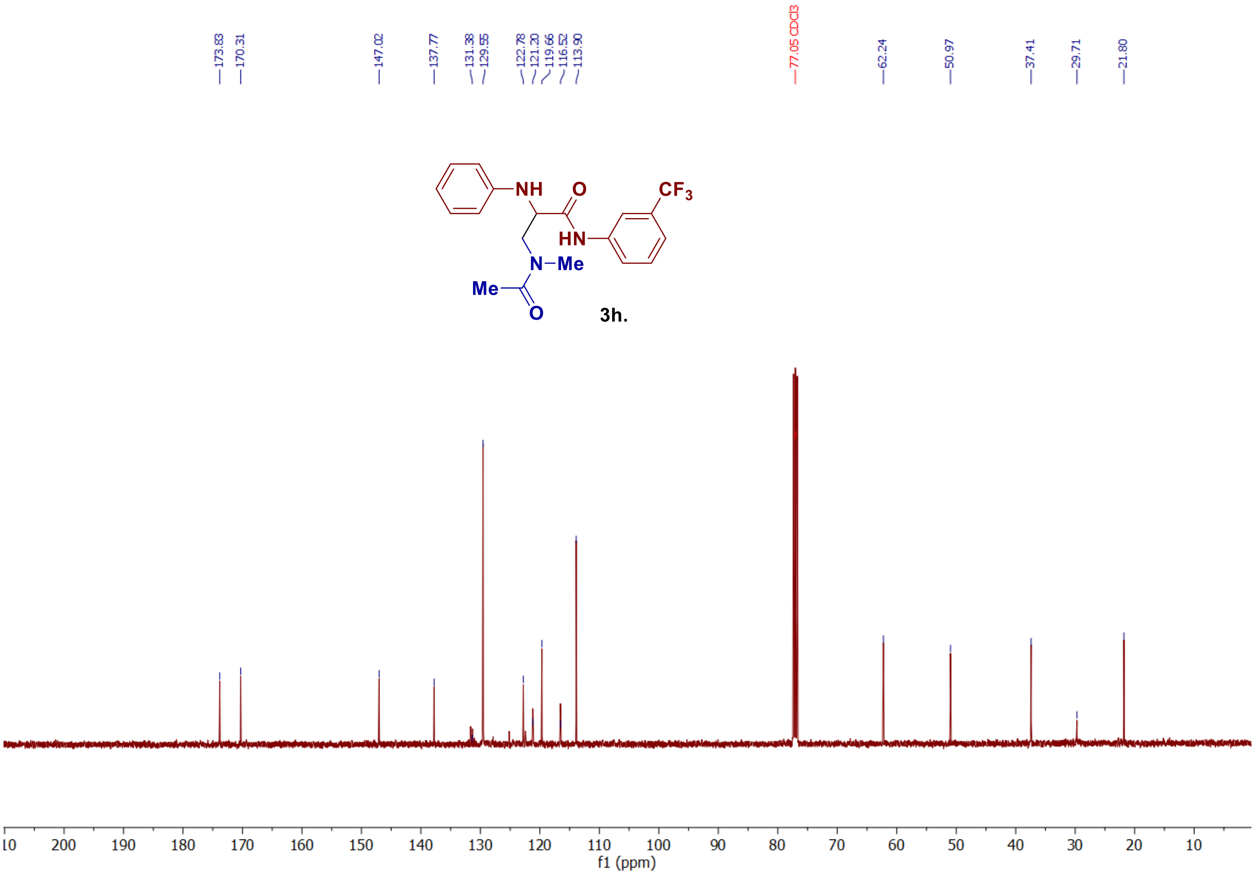

^{19}F NMR (377 MHz, $CDCl_3$)

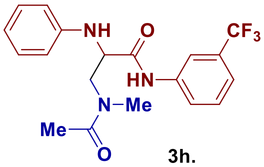

3h.

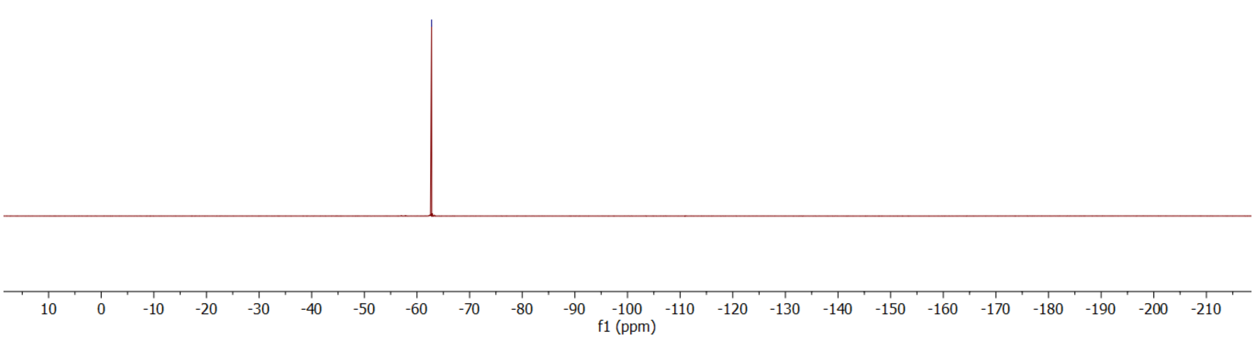

^{1}H NMR (400 MHz, $CDCl_3$)

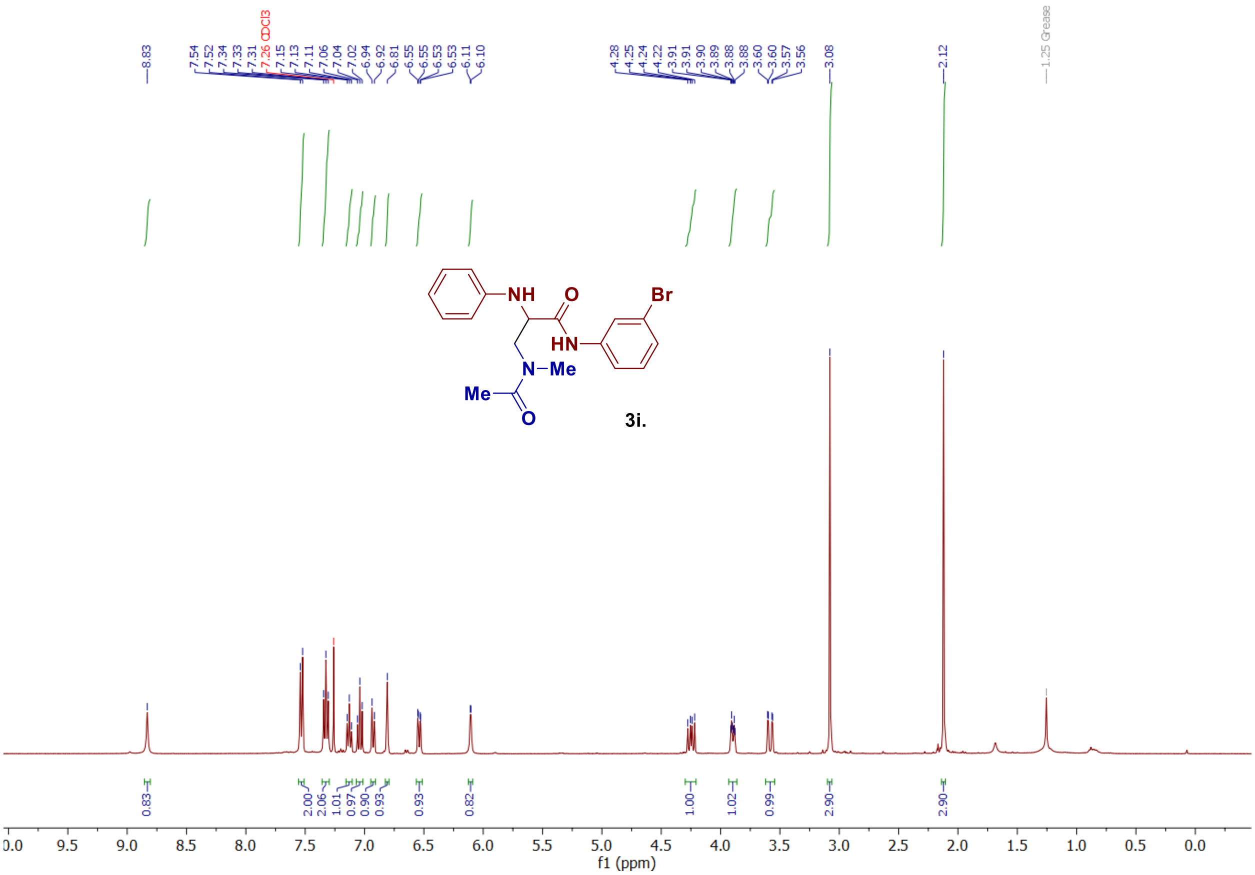

^{13}C NMR (101 MHz, $CDCl_3$)

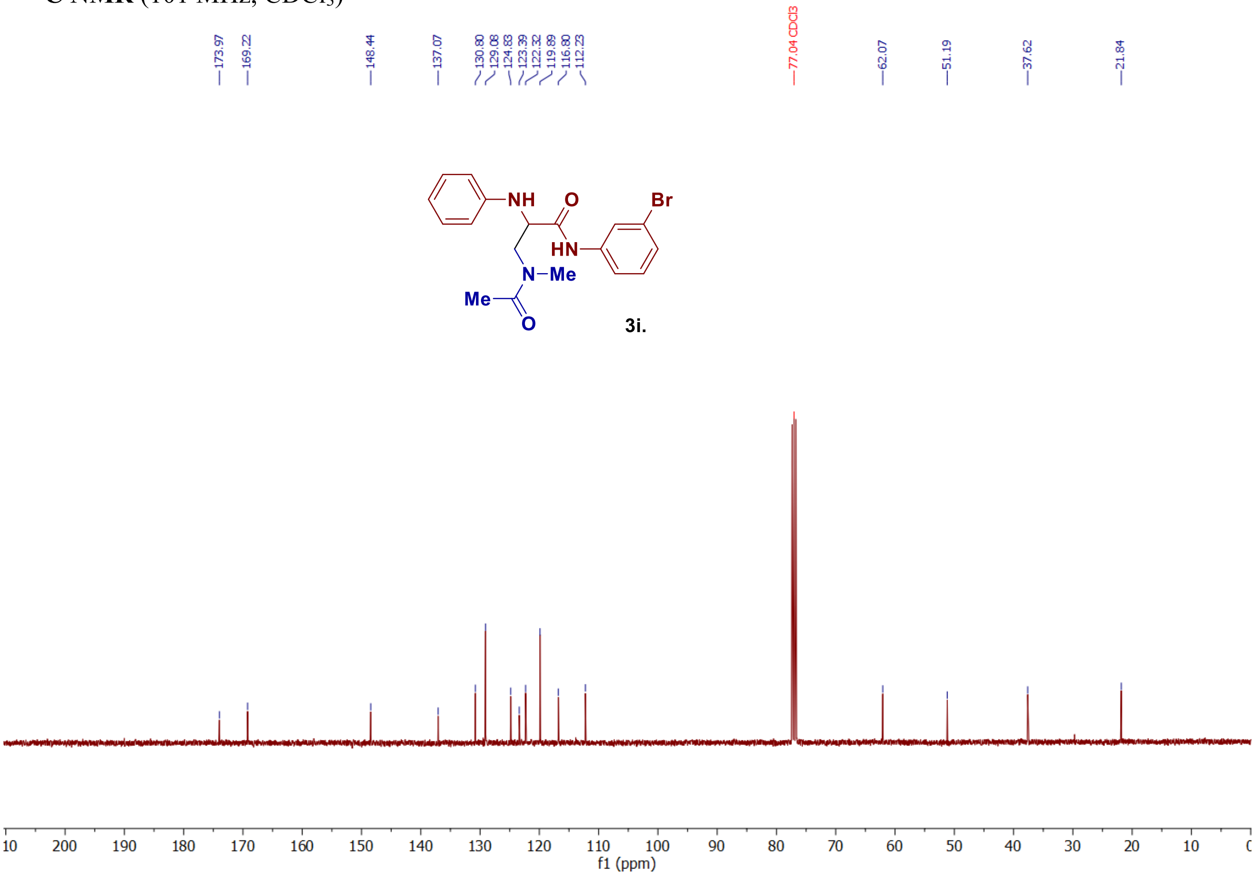

¹H NMR (400 MHz, $CDCl_3$)

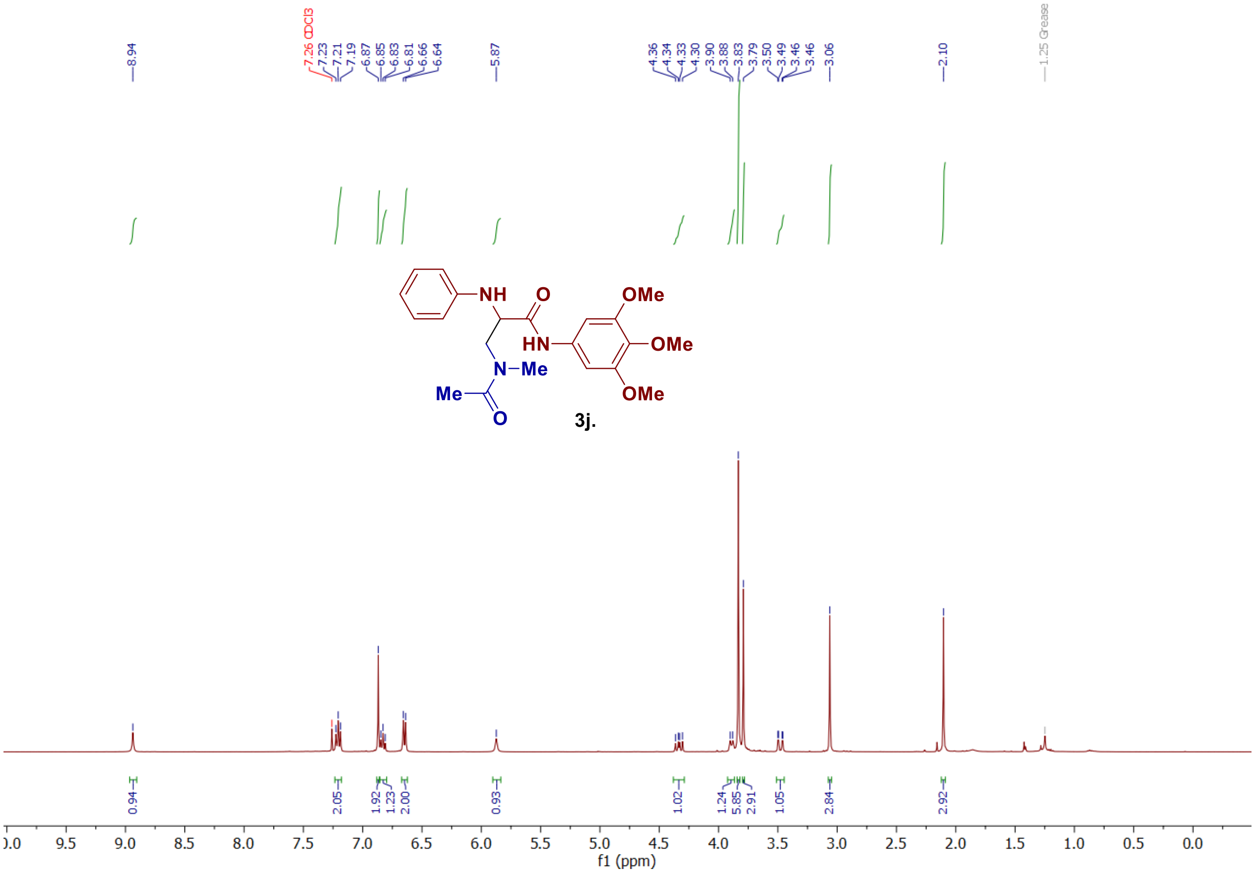

¹³C NMR (101 MHz, $CDCl_3$)

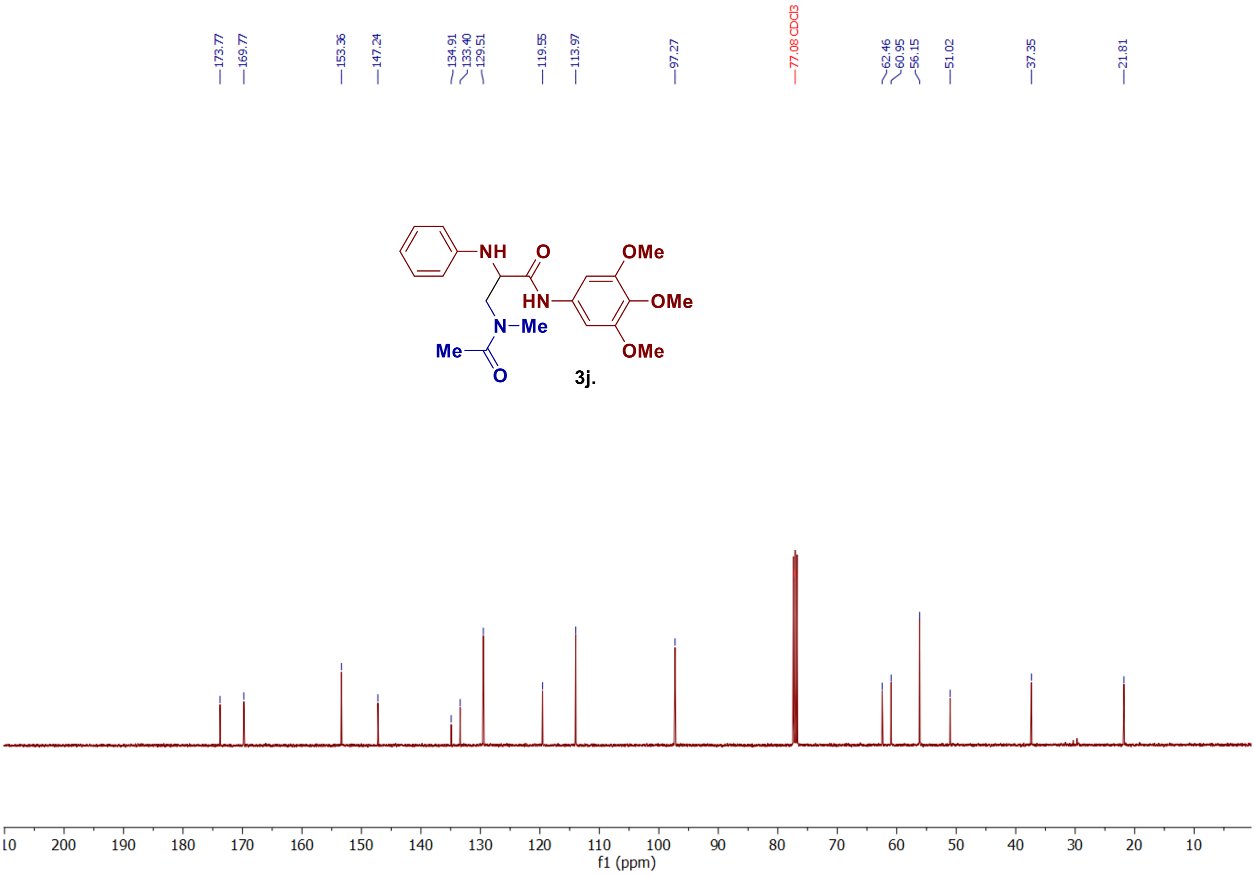

^{1}H NMR (400 MHz, $CDCl_3$)

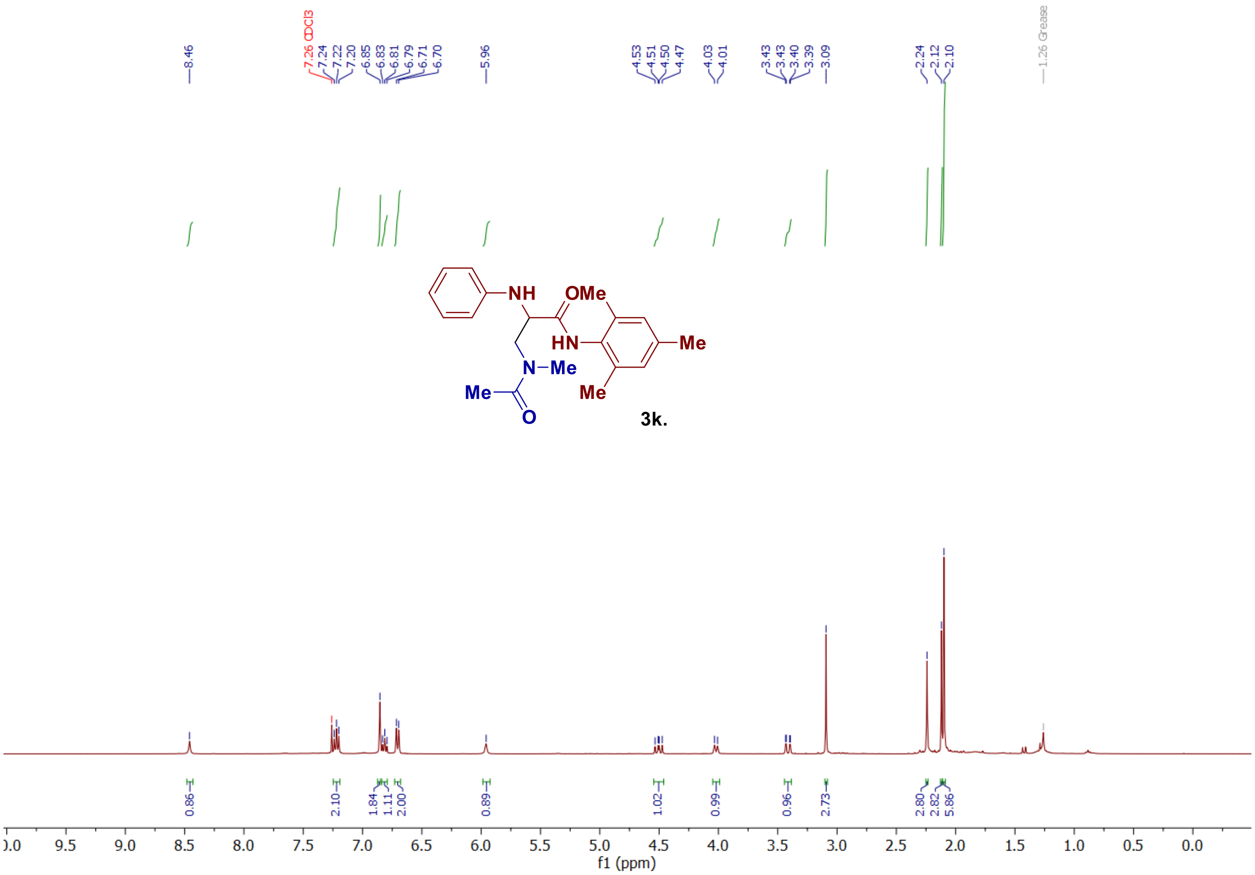

^{13}C NMR (101 MHz, $CDCl_3$)

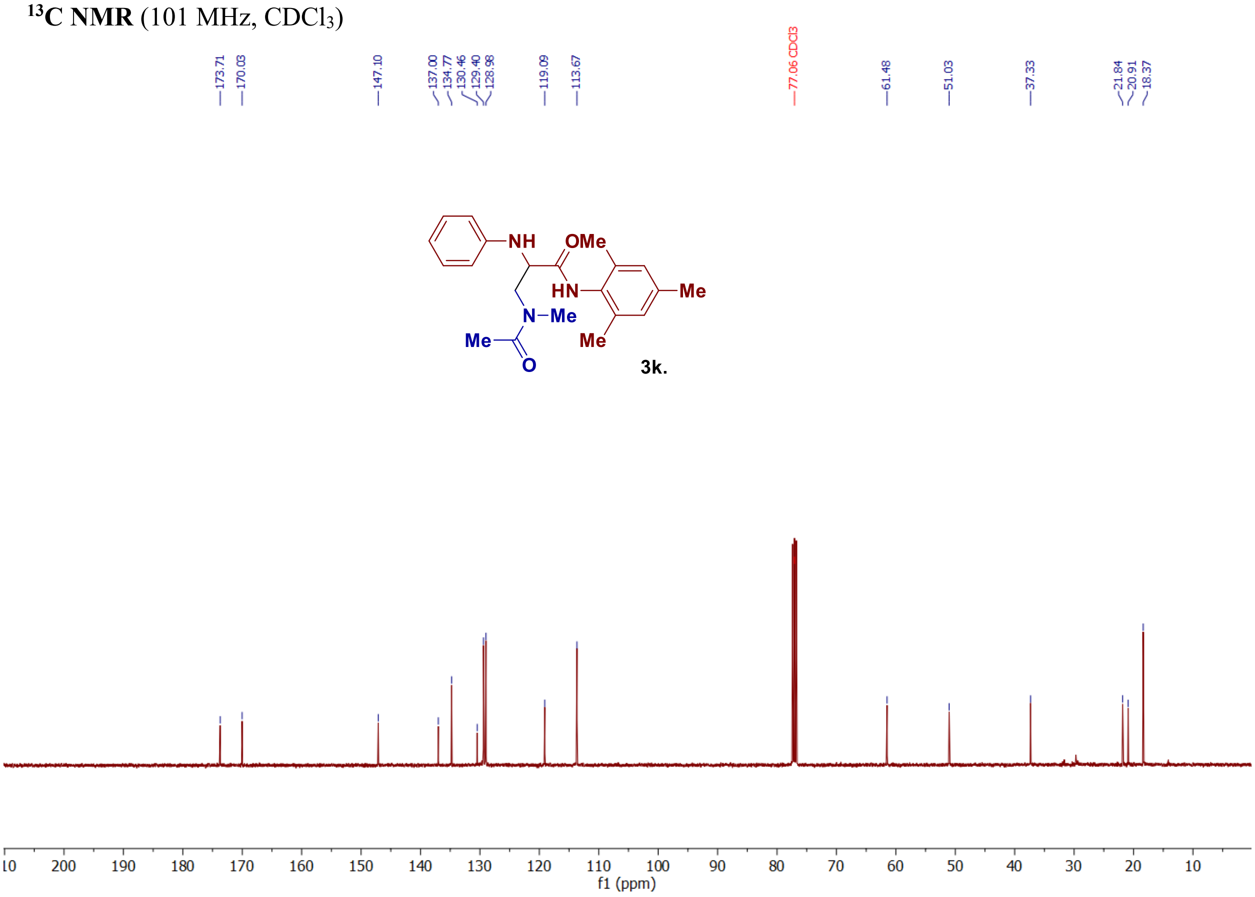

^{1}**H NMR** (400 MHz, $CDCl_3$)

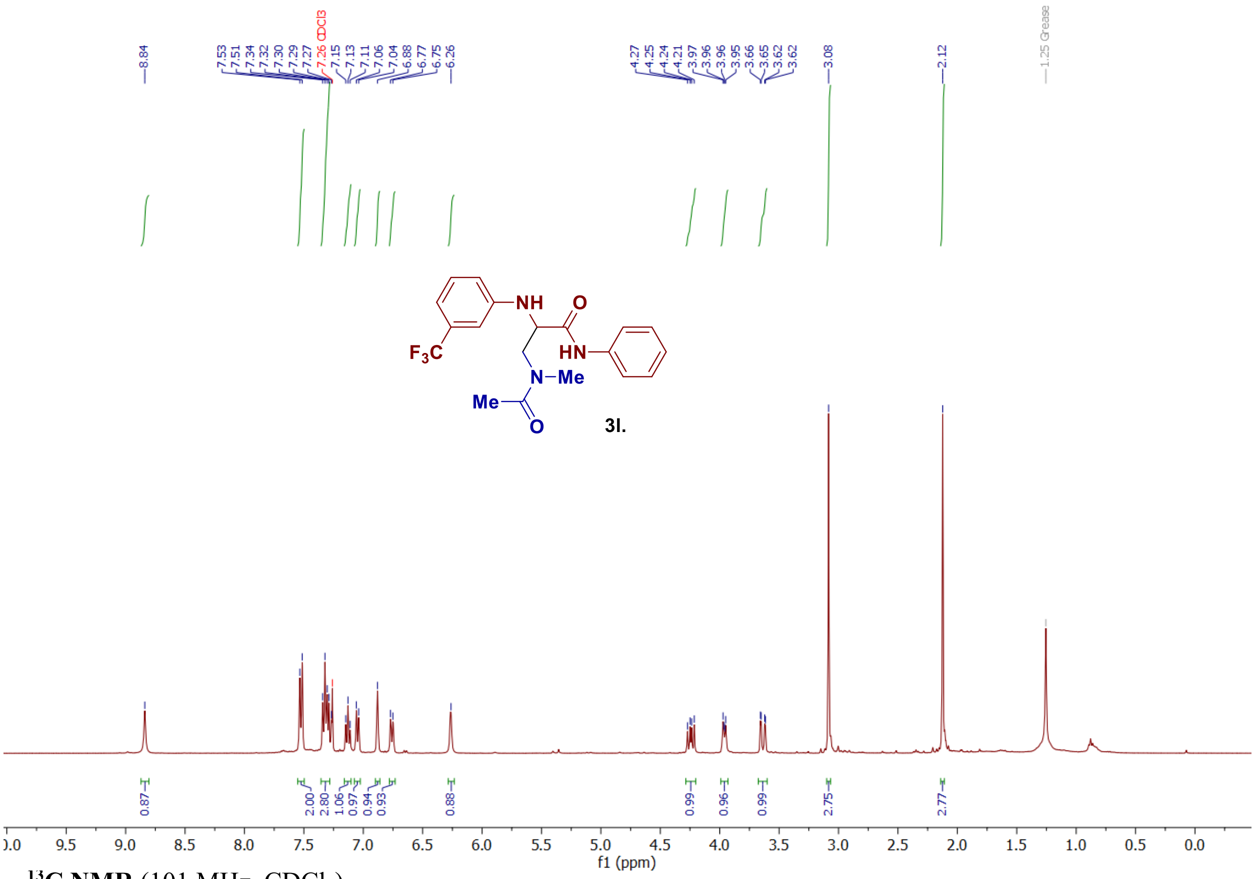

^{13}**C NMR** (101 MHz, $CDCl_3$)

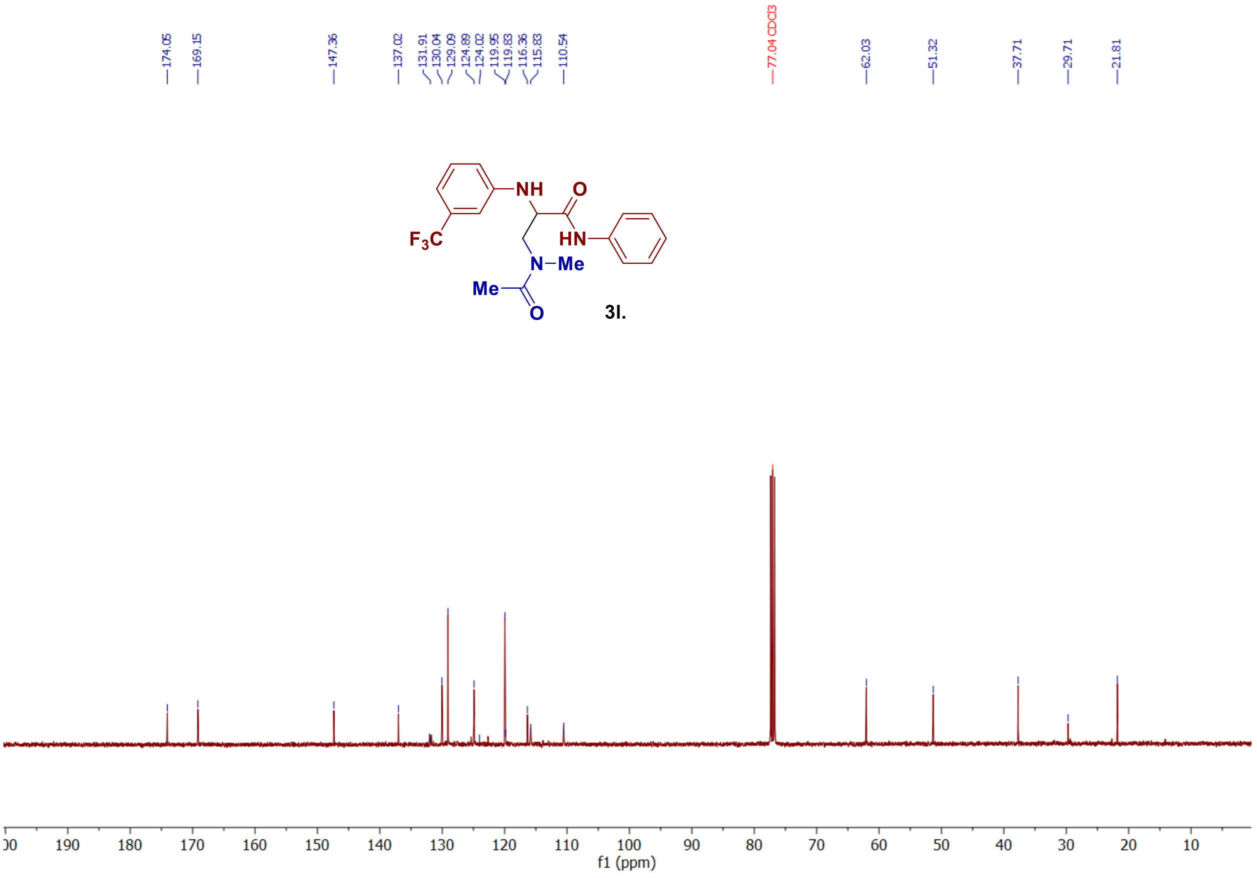

^{19}F NMR (377 MHz, $CDCl_3$)

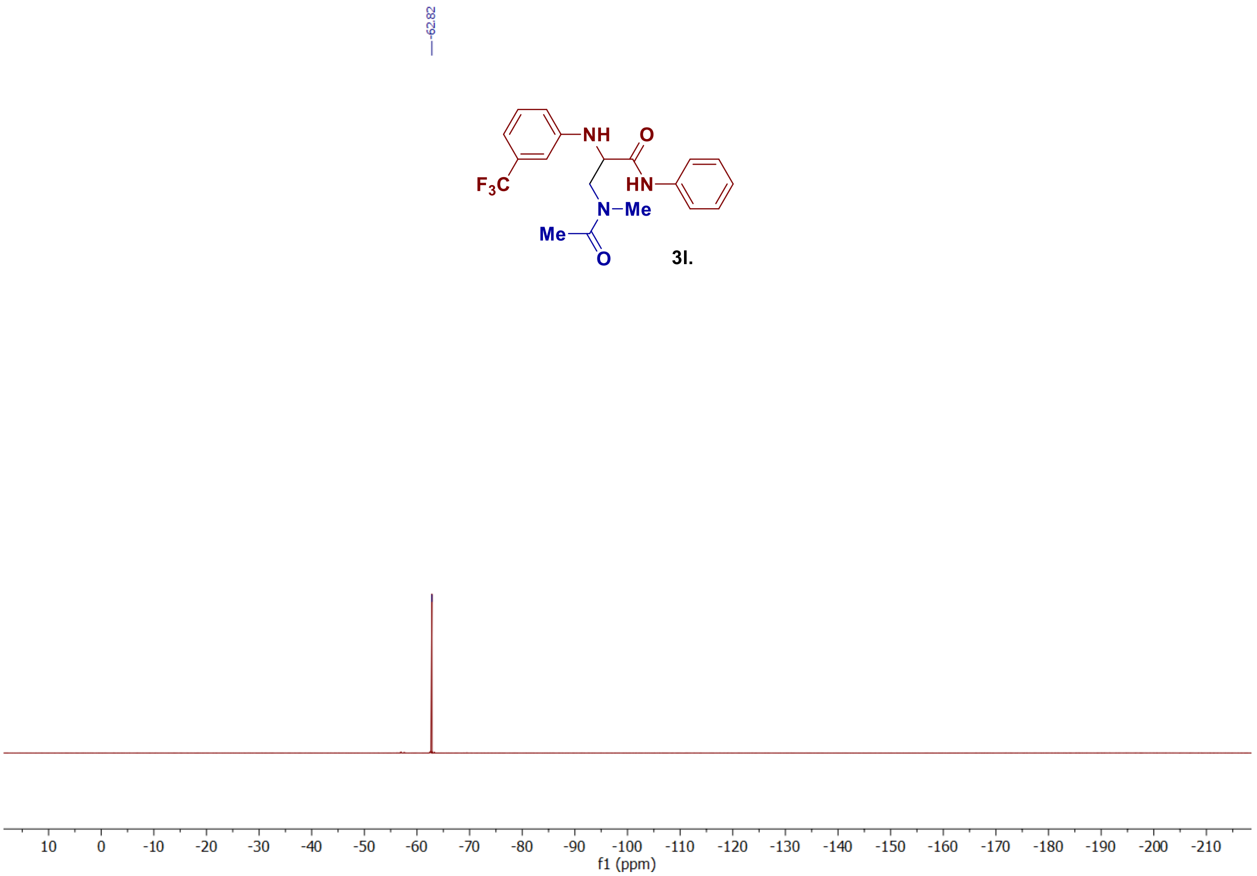

^{1}H NMR (400 MHz, $CDCl_3$)

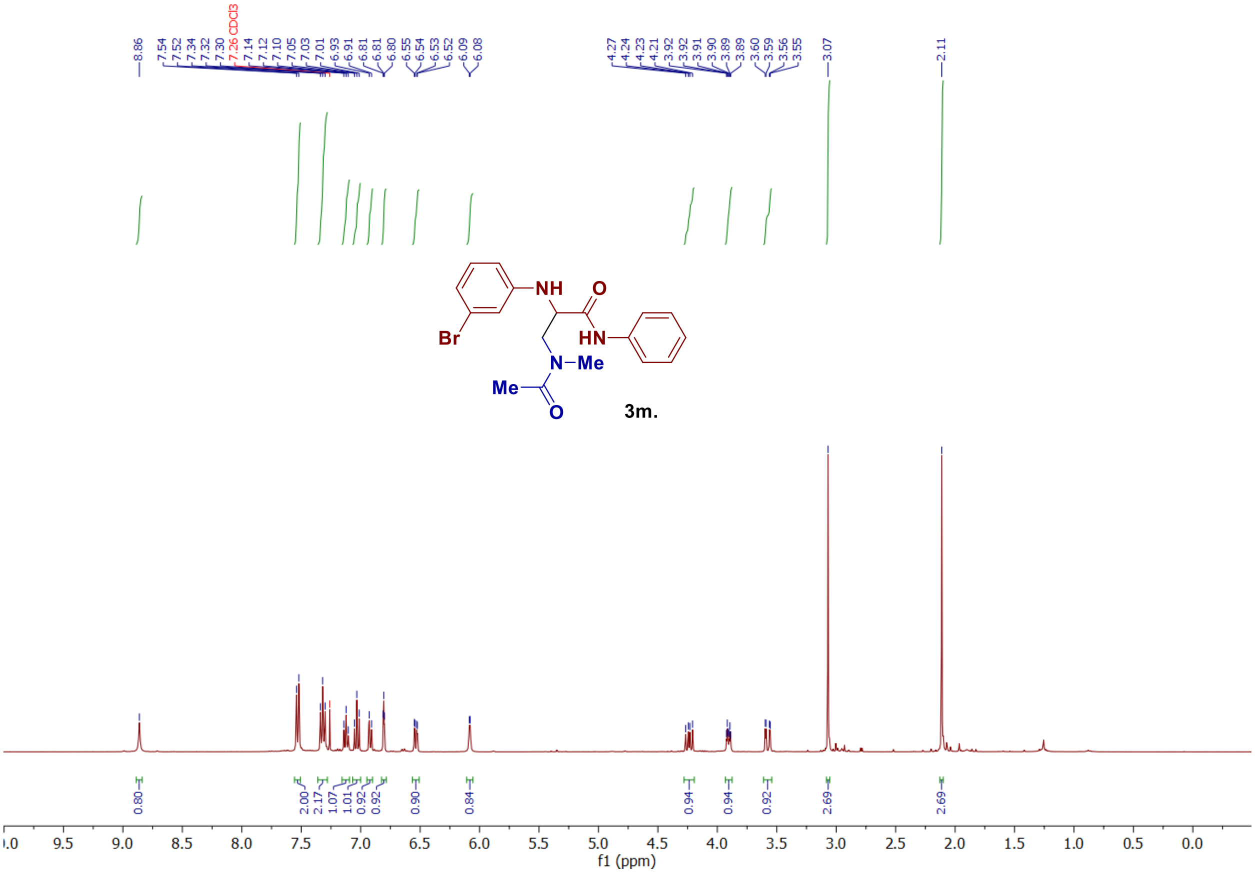

^{13}C NMR (101 MHz, $CDCl_3$)

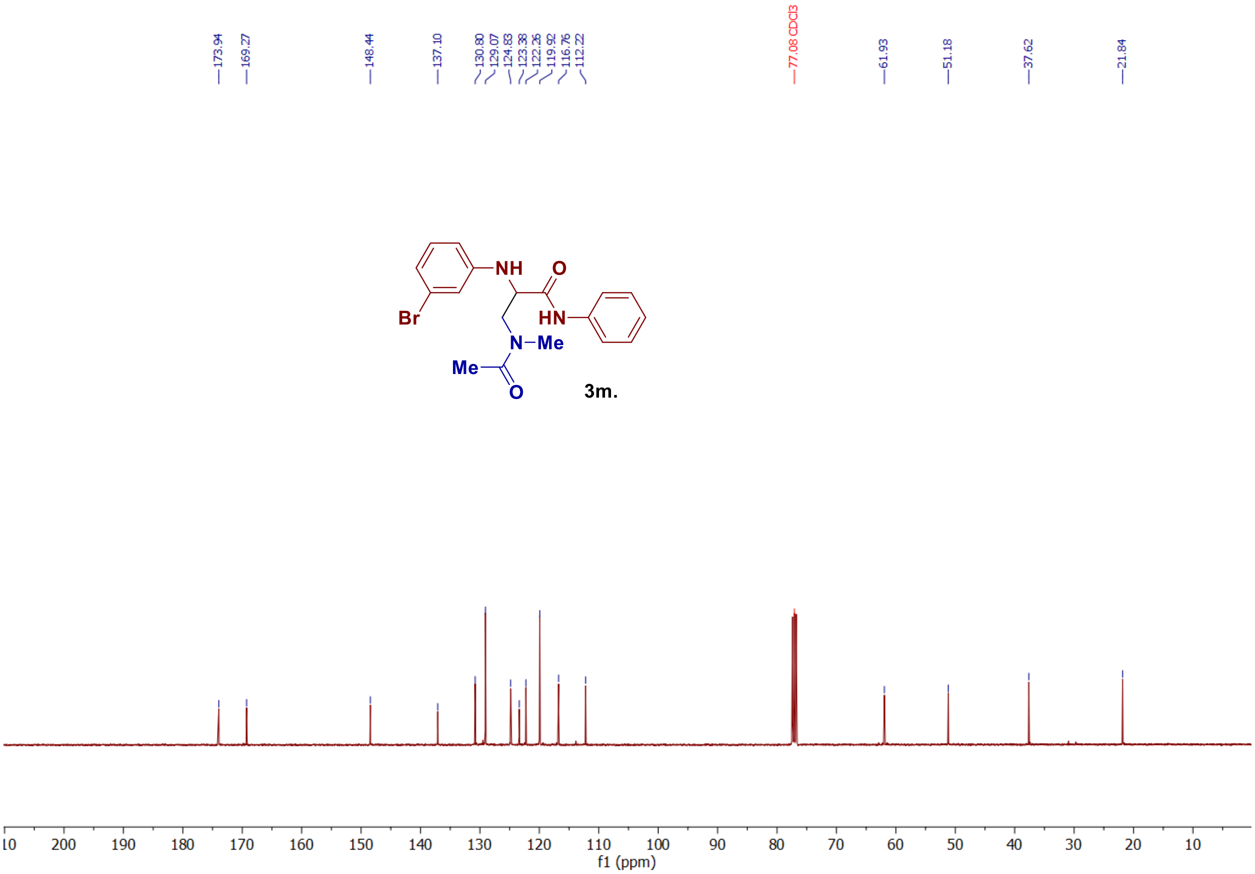

^{1}H NMR (400 MHz, $CDCl_3$)

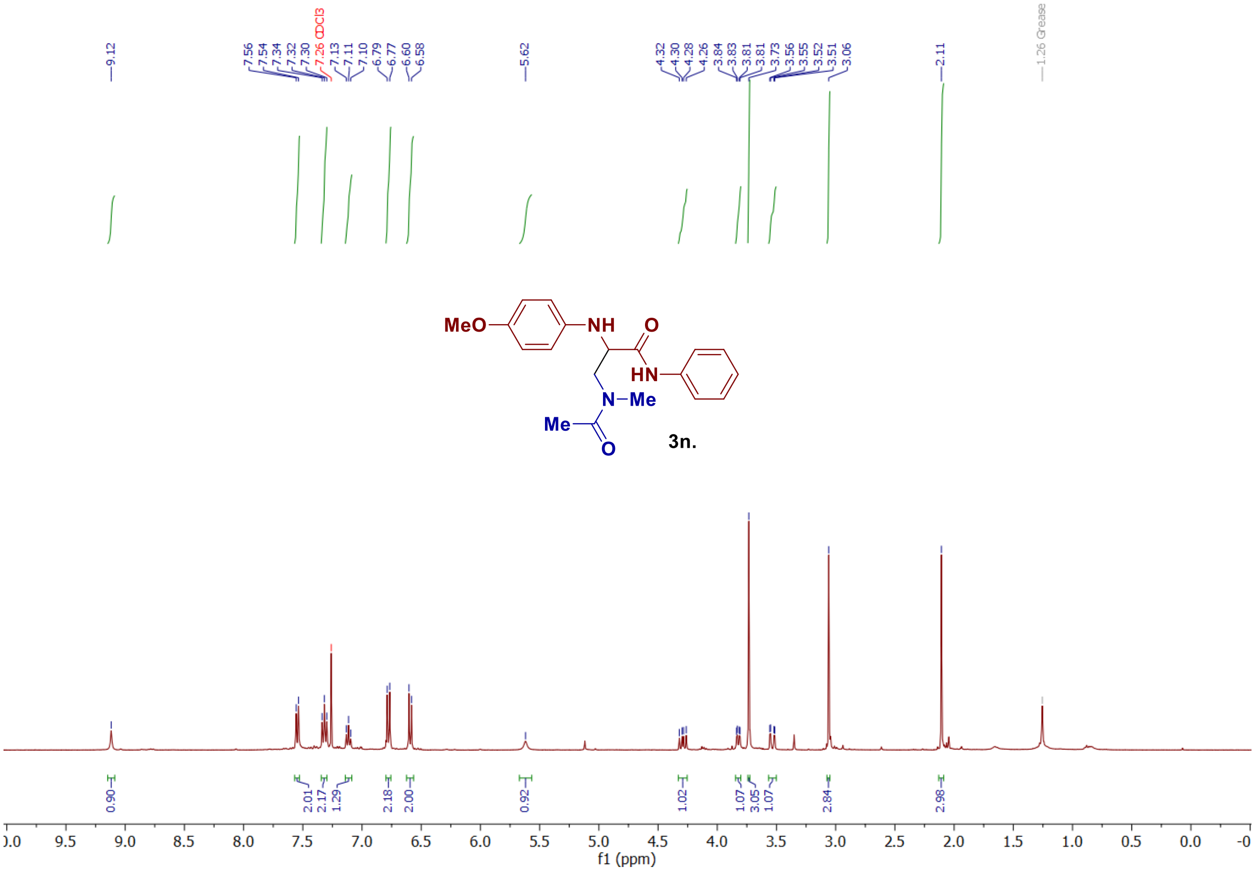

^{13}C NMR (101 MHz, $CDCl_3$)

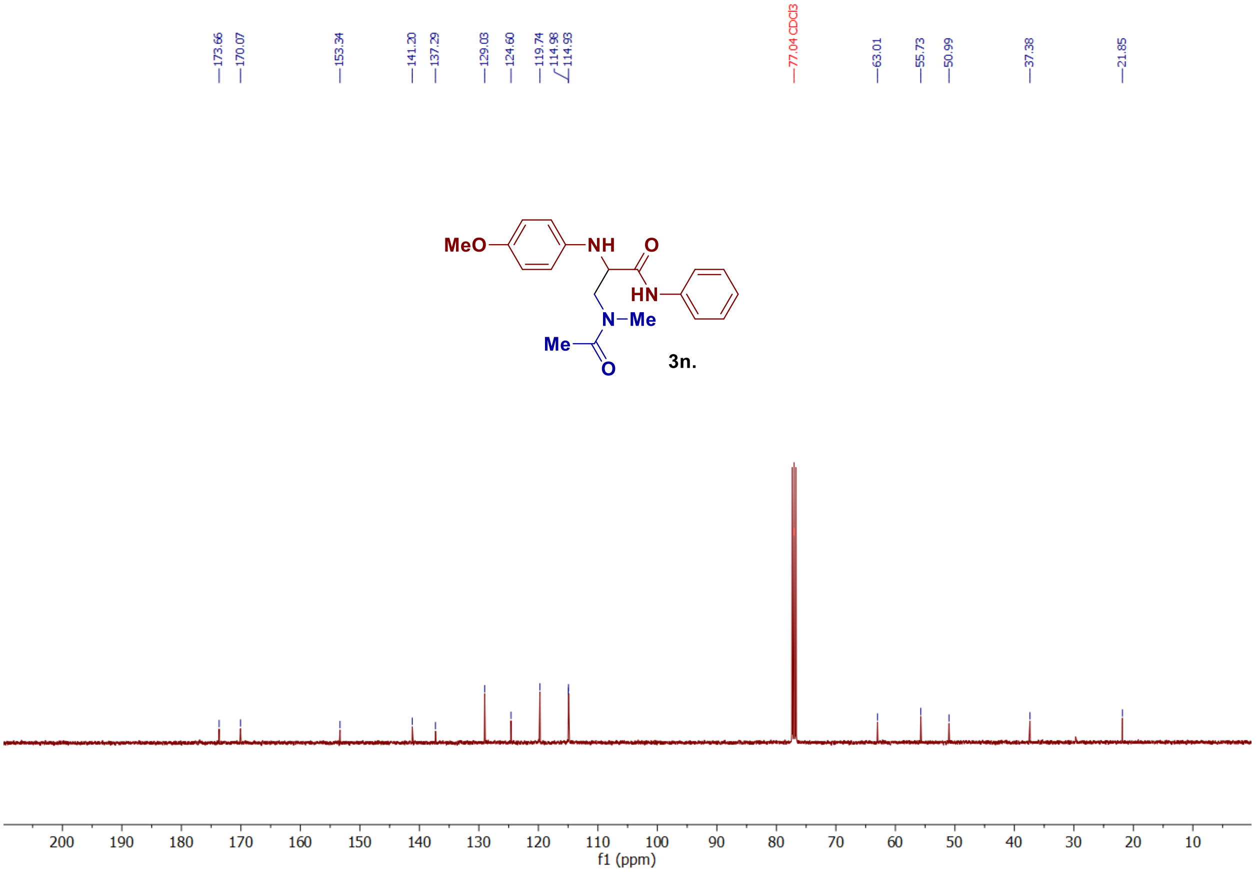

^{1}H NMR (400 MHz, $CDCl_3$)

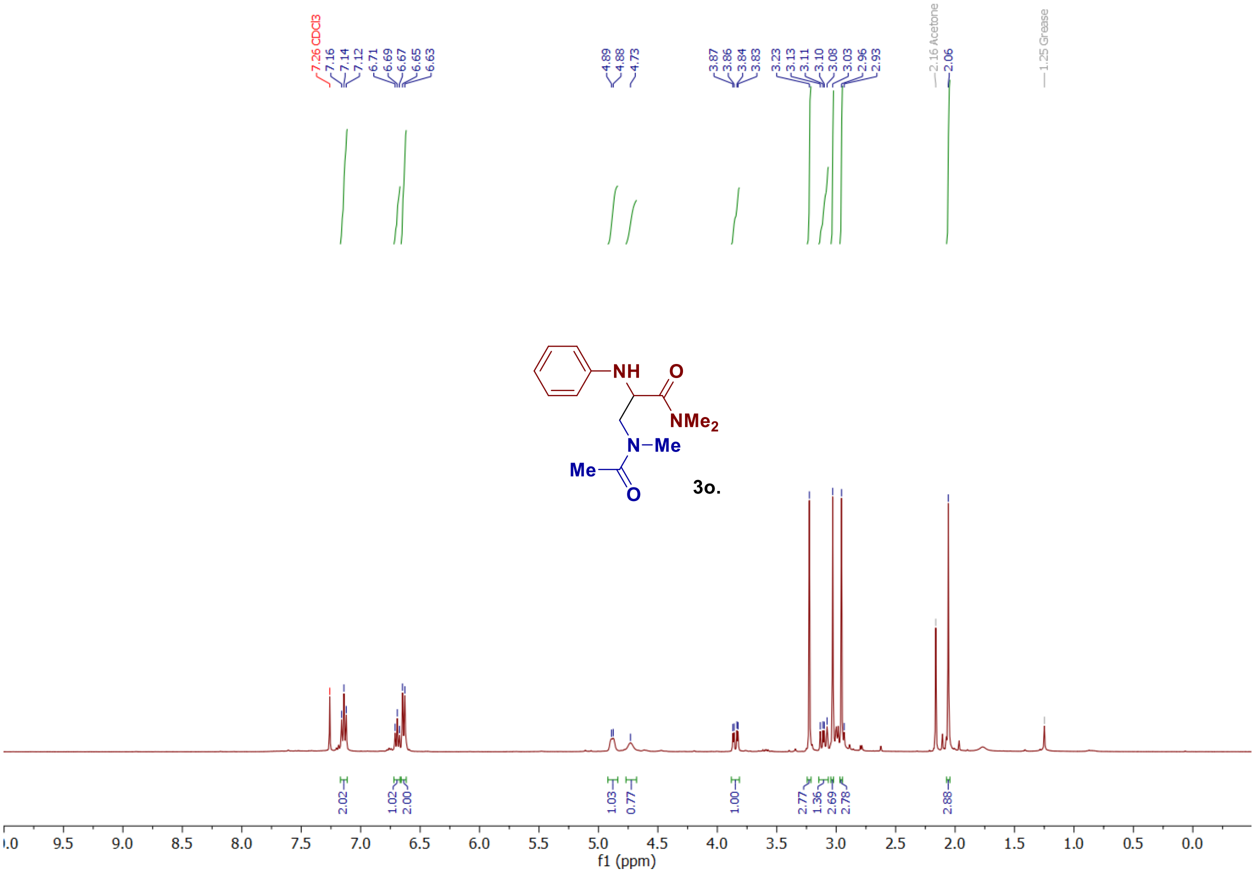

^{13}C NMR (101 MHz, $CDCl_3$)

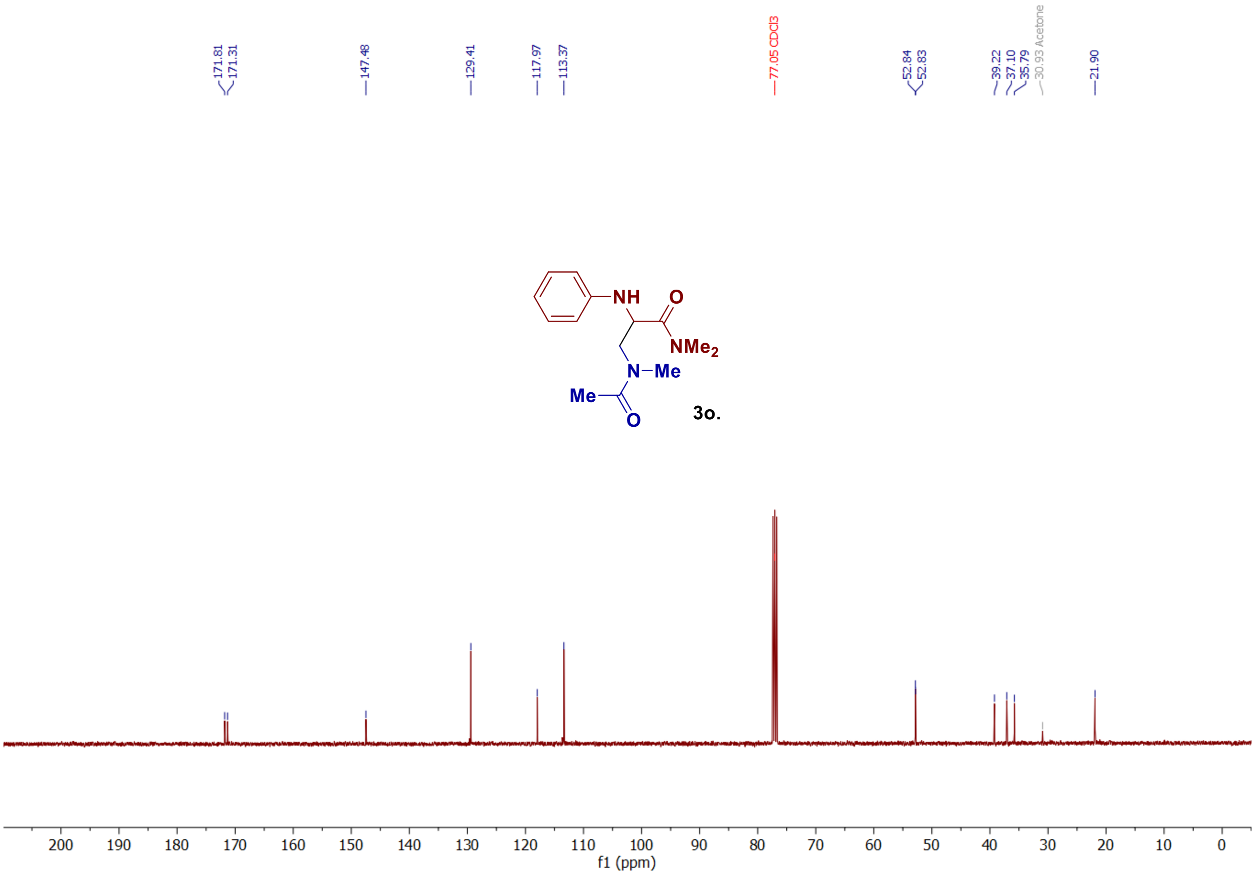

^{1}H NMR (400 MHz, $CDCl_3$)

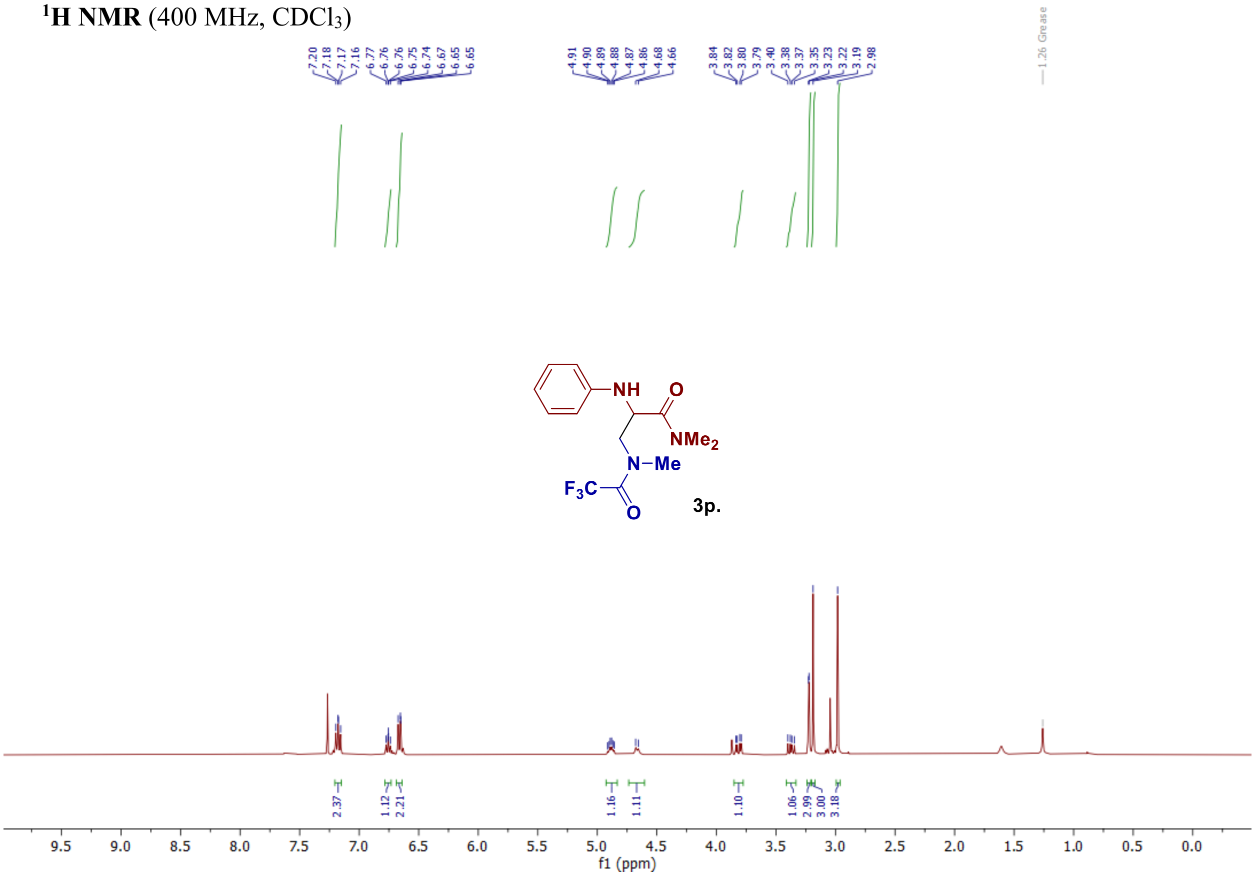

^{13}C NMR (101 MHz, $CDCl_3$)

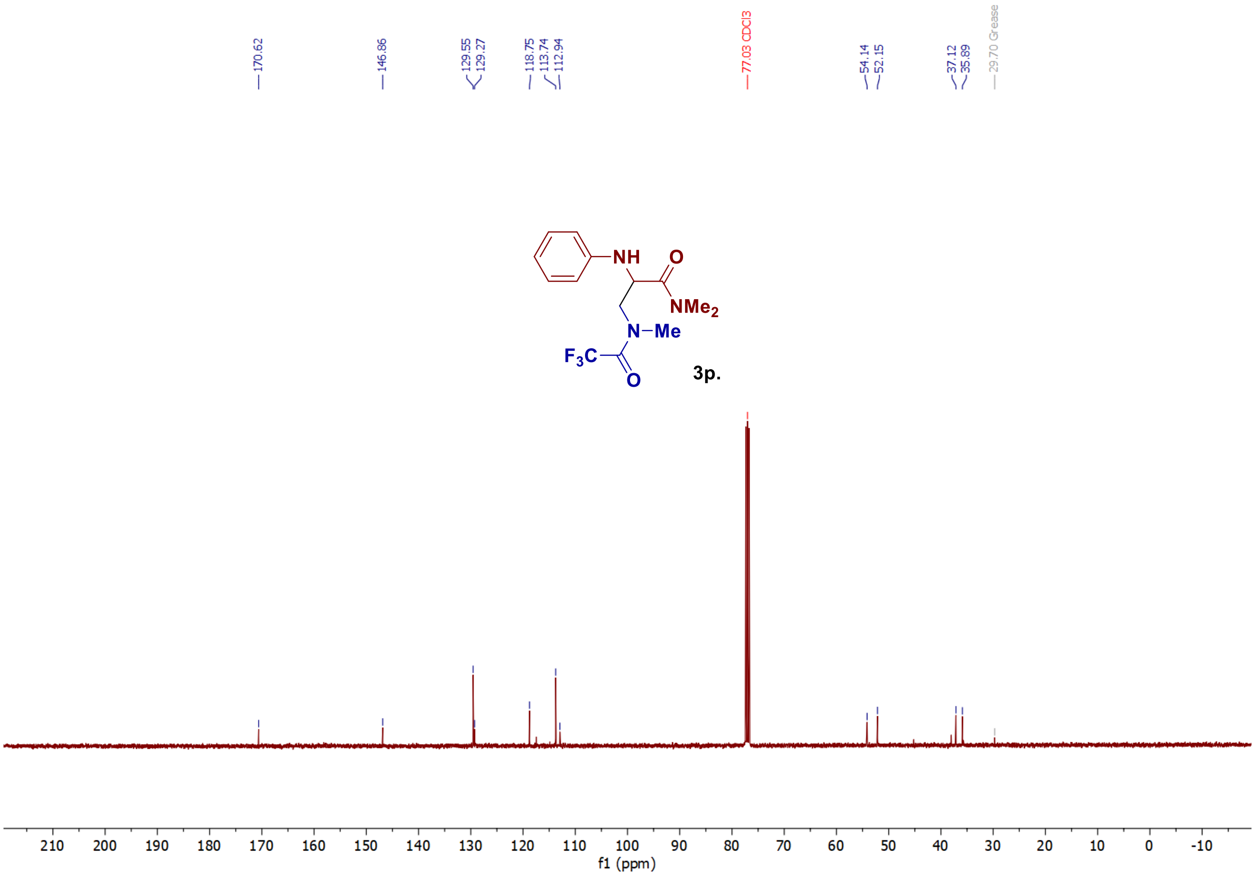

^{19}F NMR (377 MHz, $CDCl_3$)

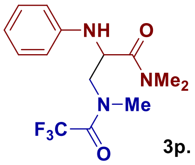

3p.

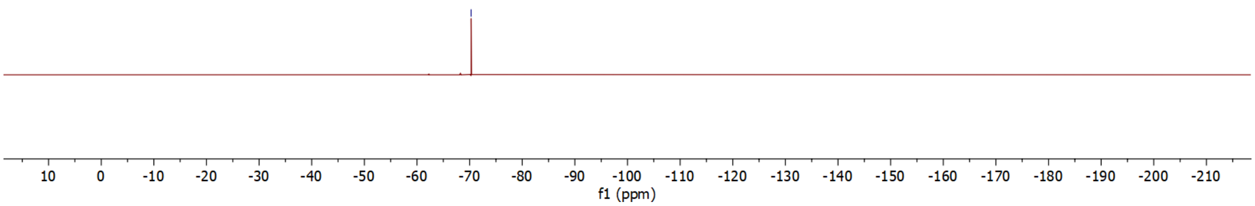

^{1}H NMR (400 MHz, $CDCl_3$)

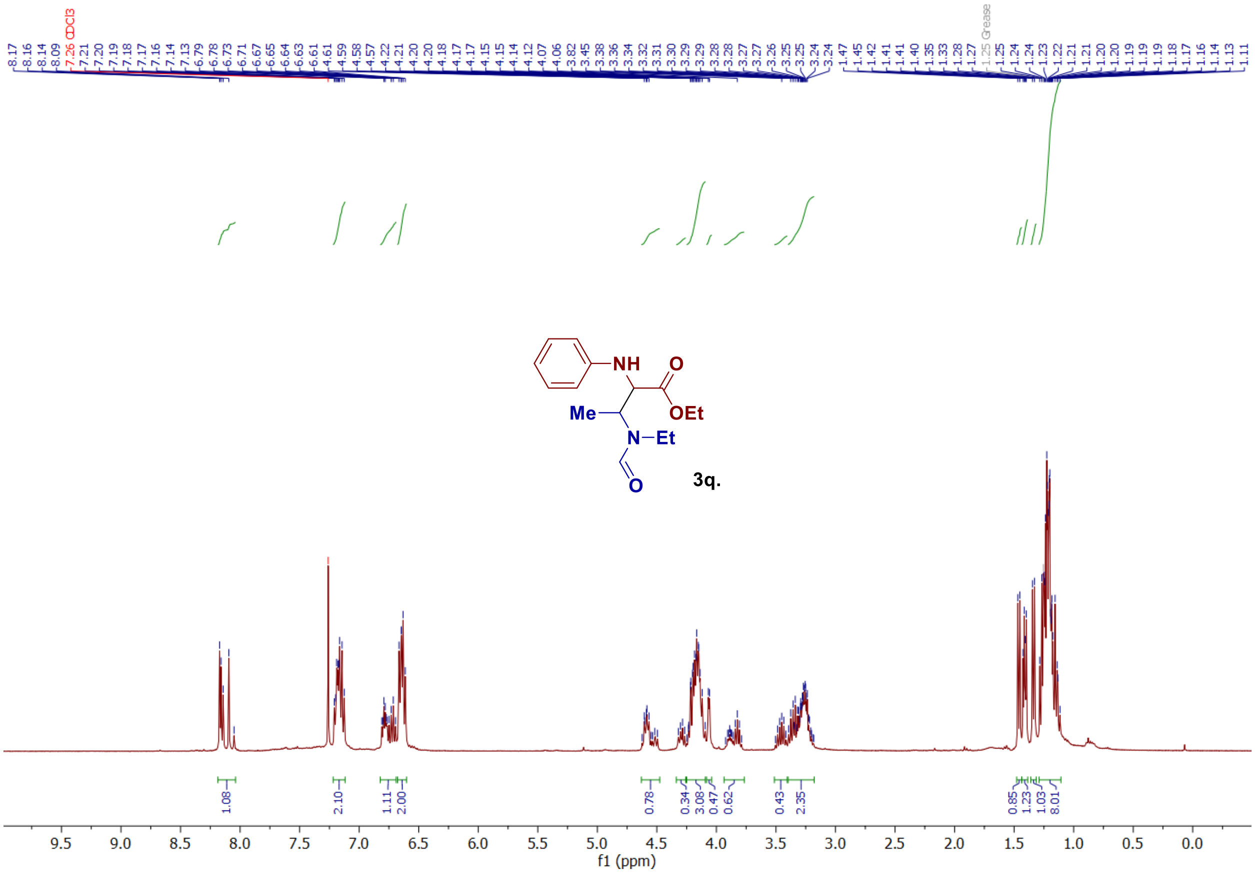

^{13}C NMR (101 MHz, $CDCl_3$)

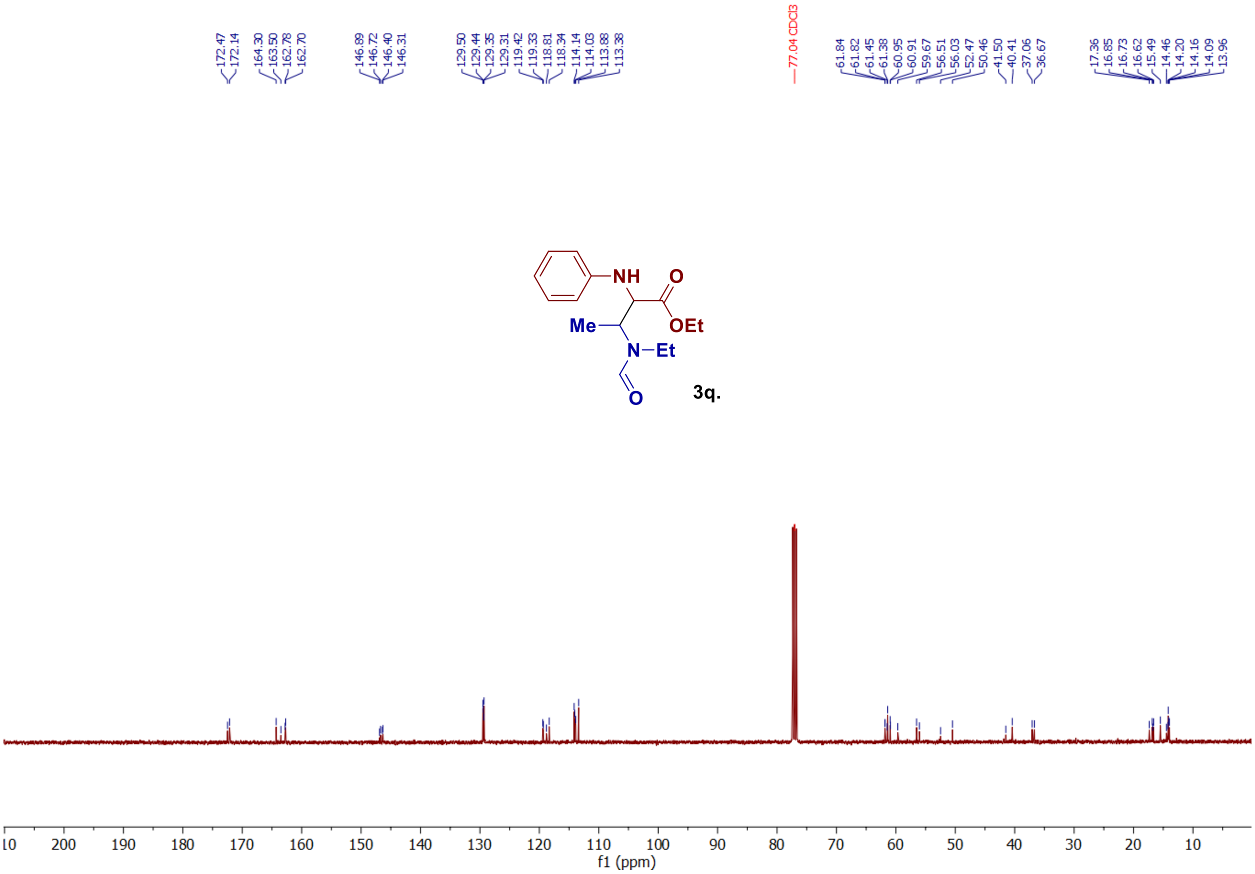

^{1}H NMR (400 MHz, $CDCl_3$)

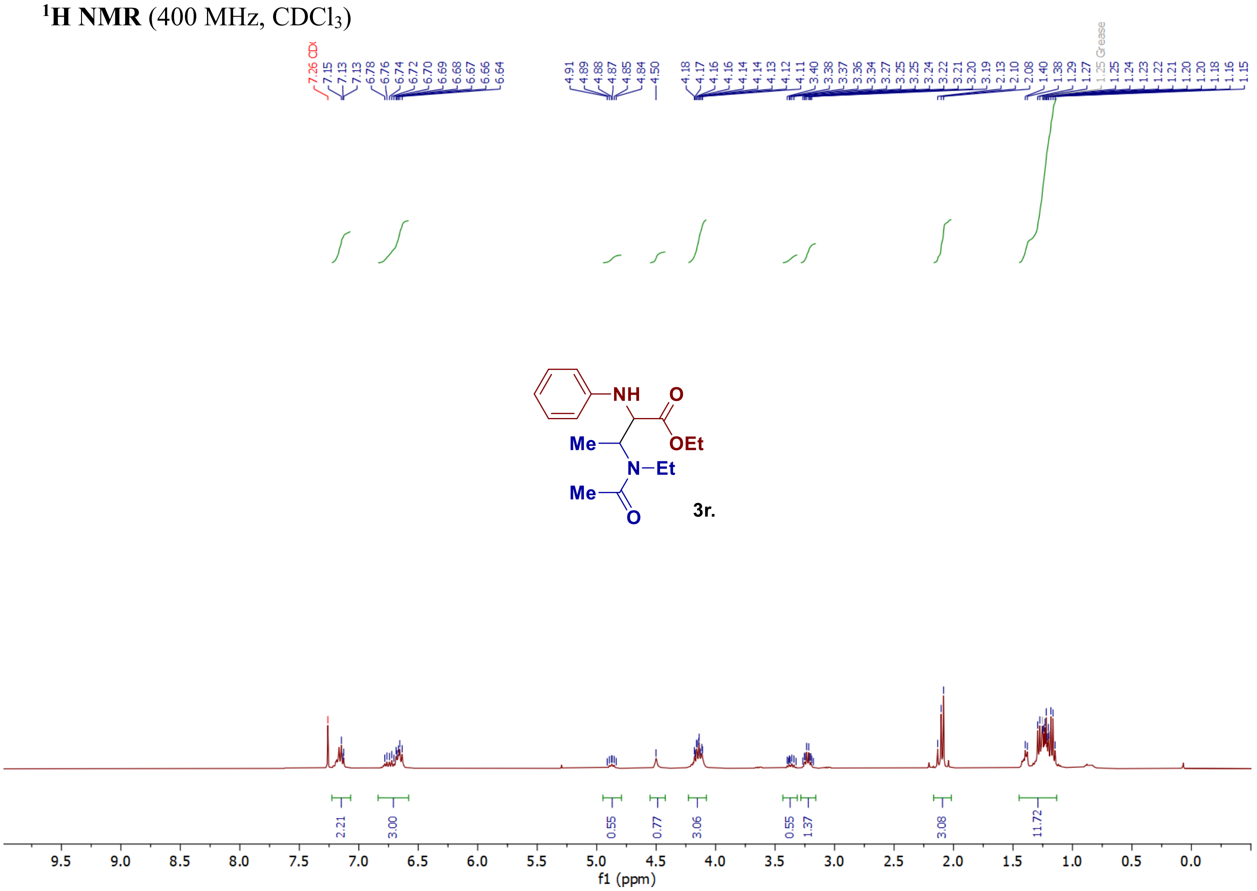

3r.

^{13}C NMR (101 MHz, $CDCl_3$)

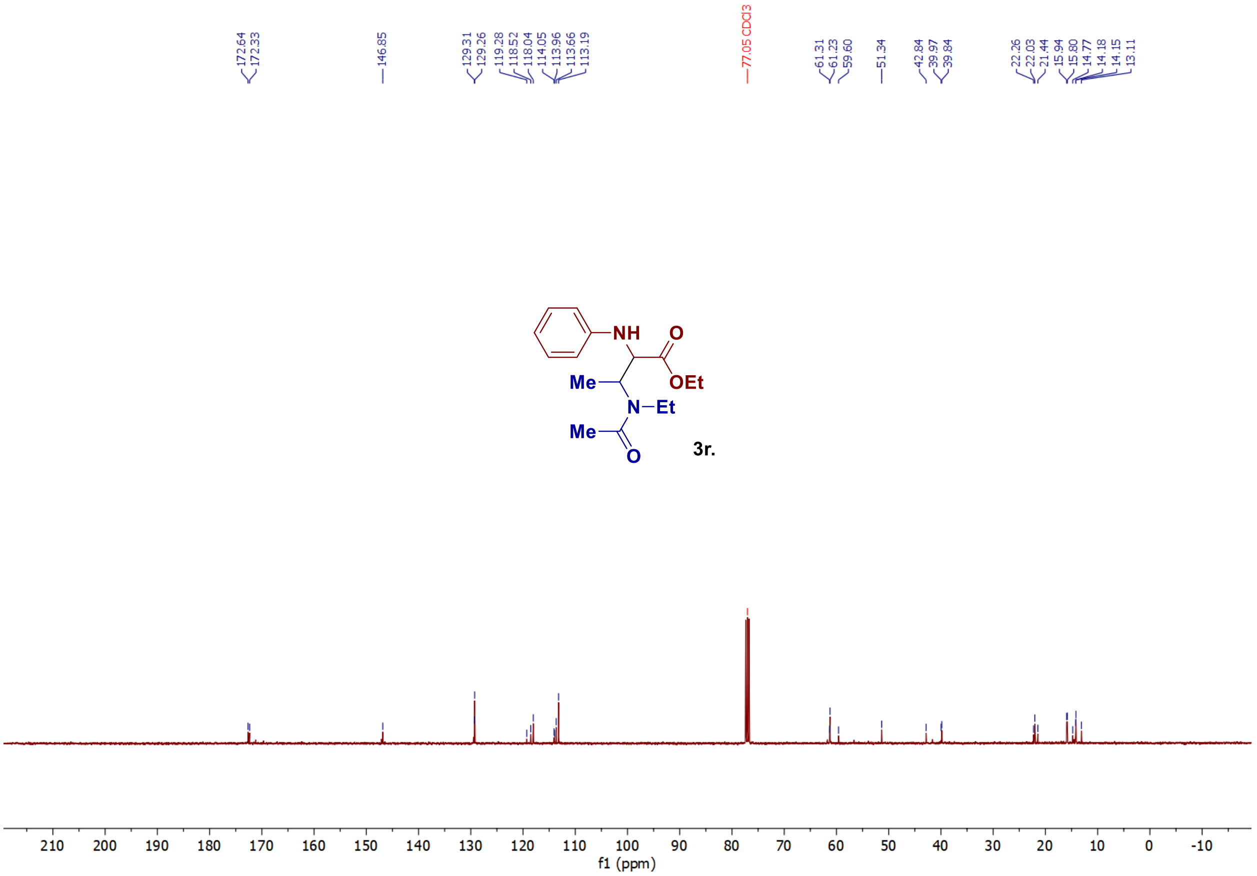

3r.

^{1}H NMR (400 MHz, $CDCl_3$)

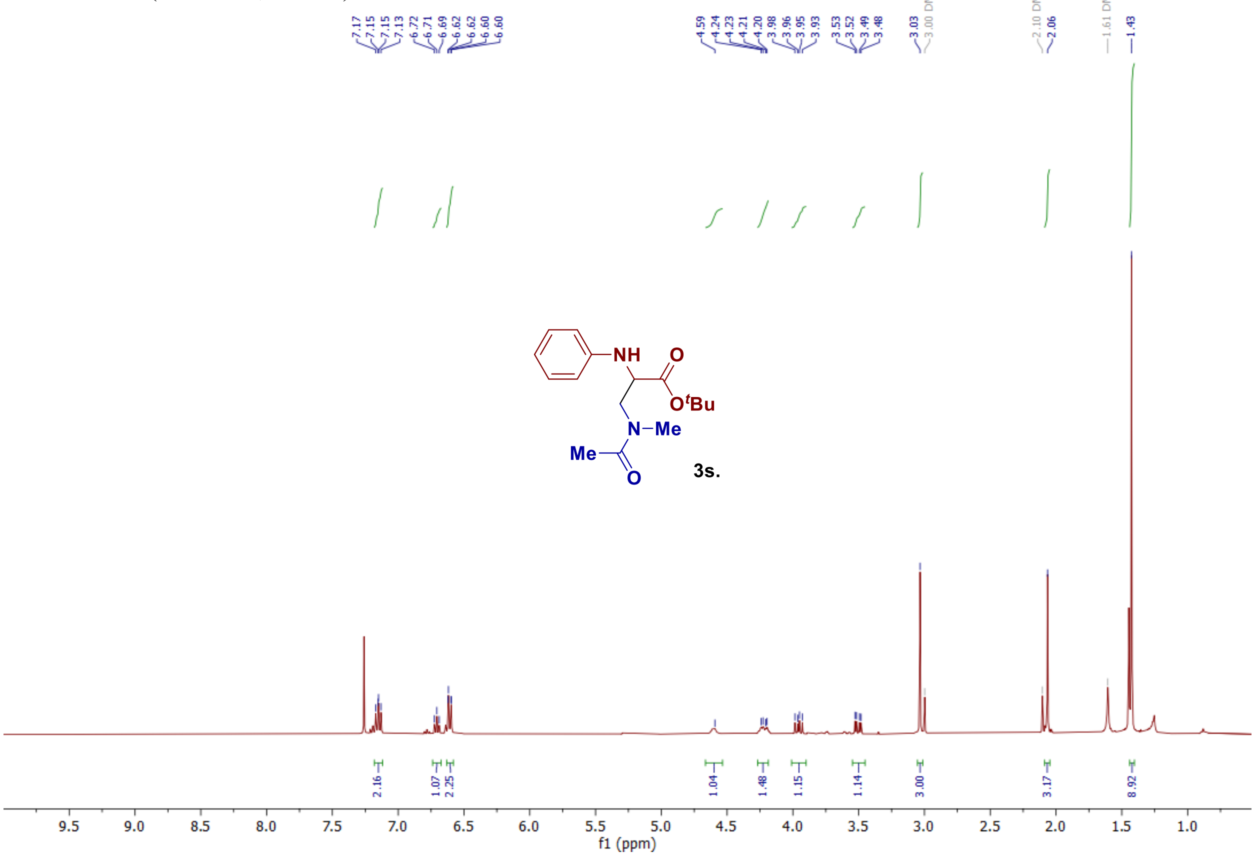

^{13}C NMR (101 MHz, $CDCl_3$)

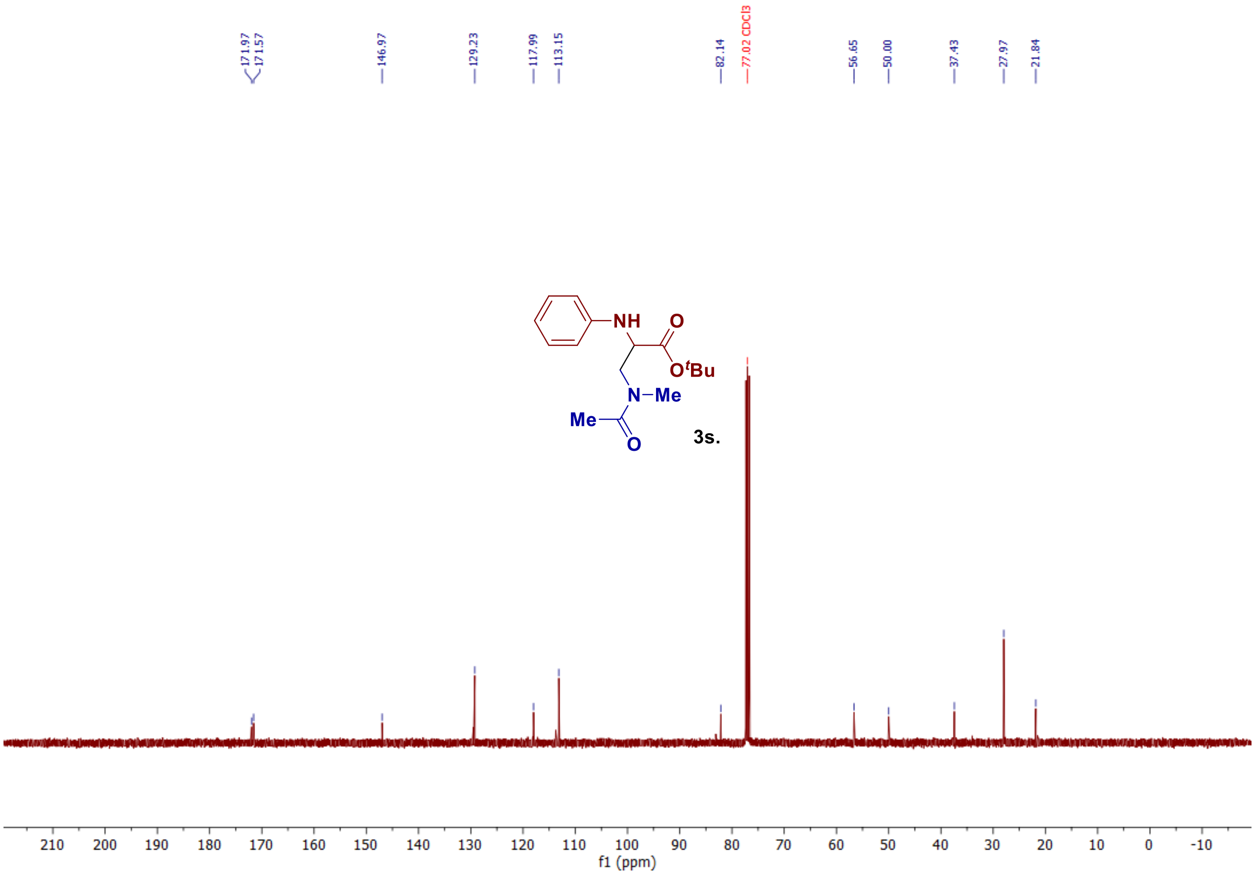

^{1}H NMR (400 MHz, $CDCl_3$)

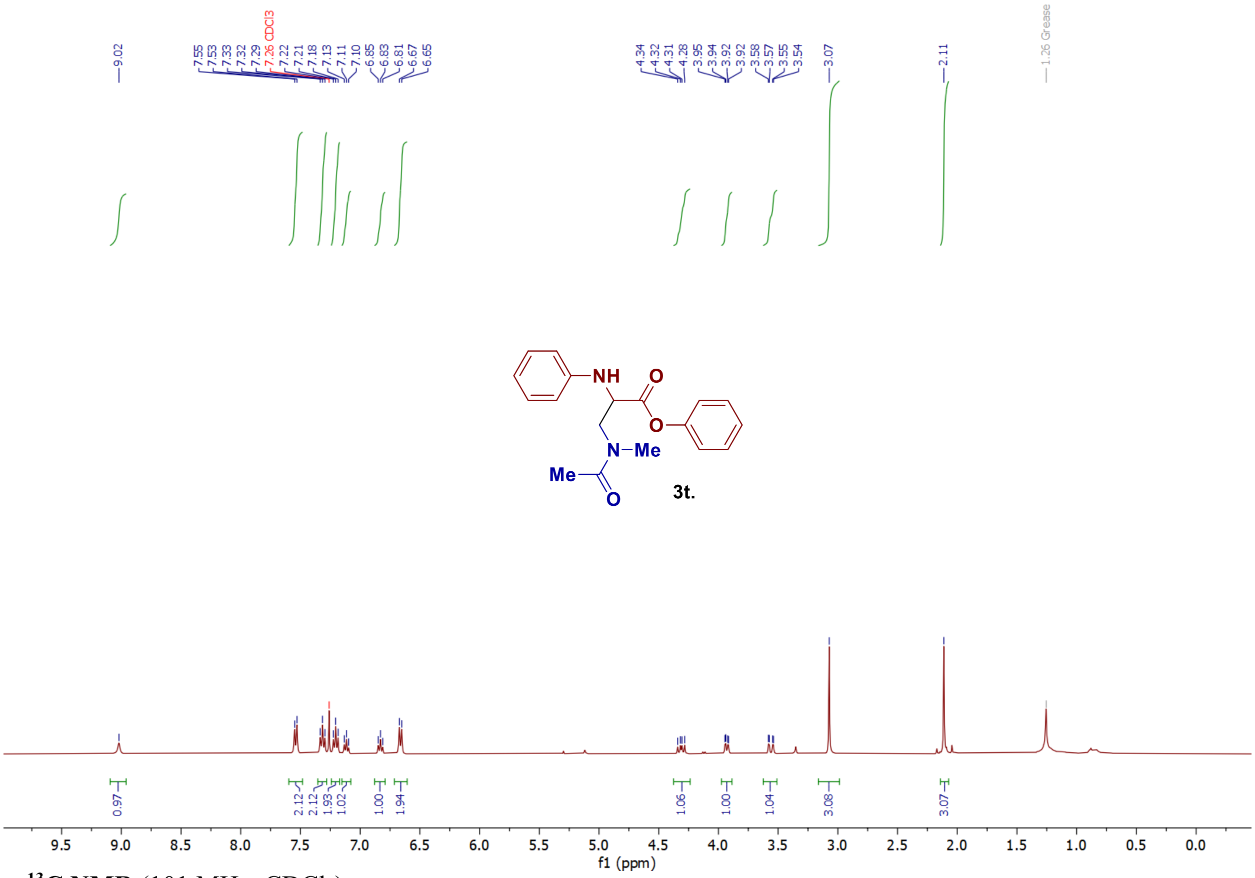

^{13}C NMR (101 MHz, $CDCl_3$)

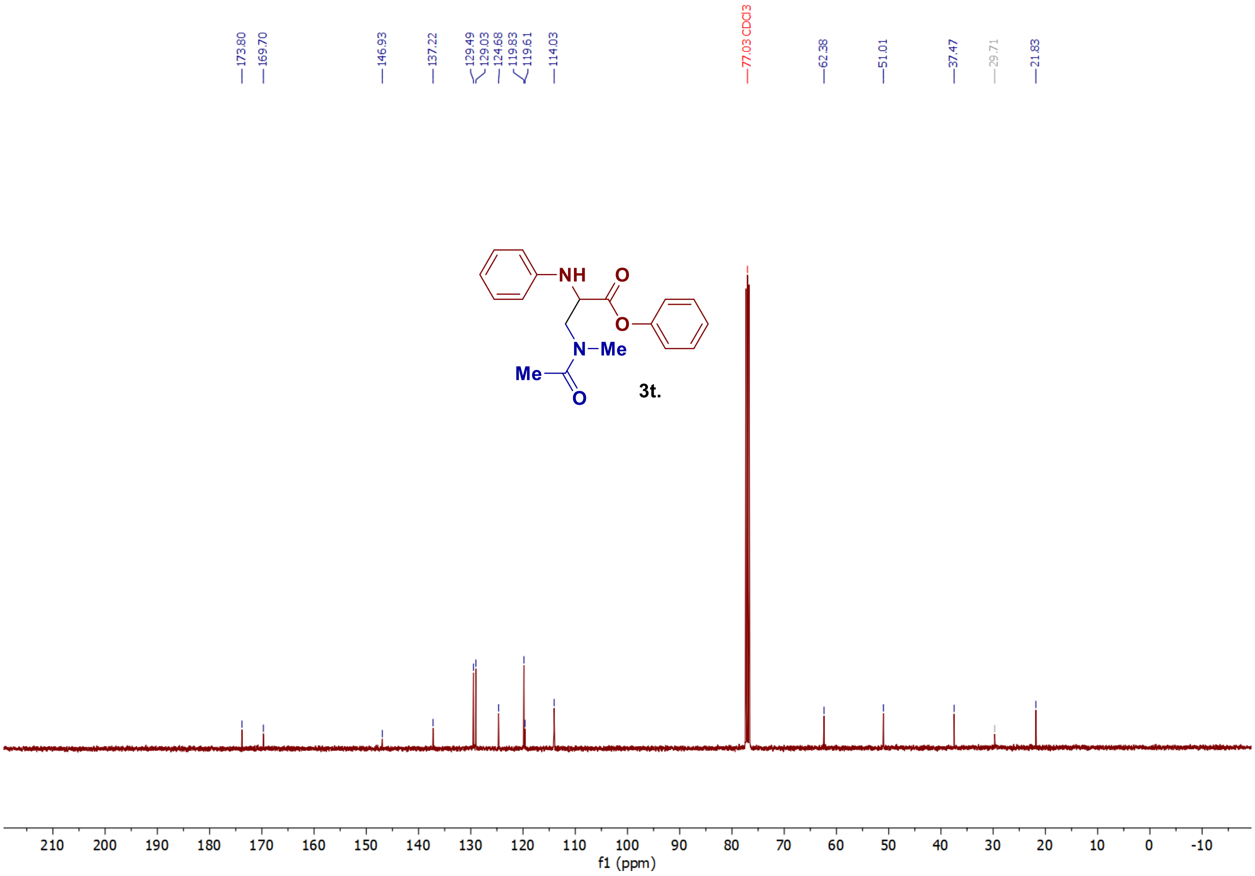

^{1}H NMR (400 MHz, $CDCl_3$)

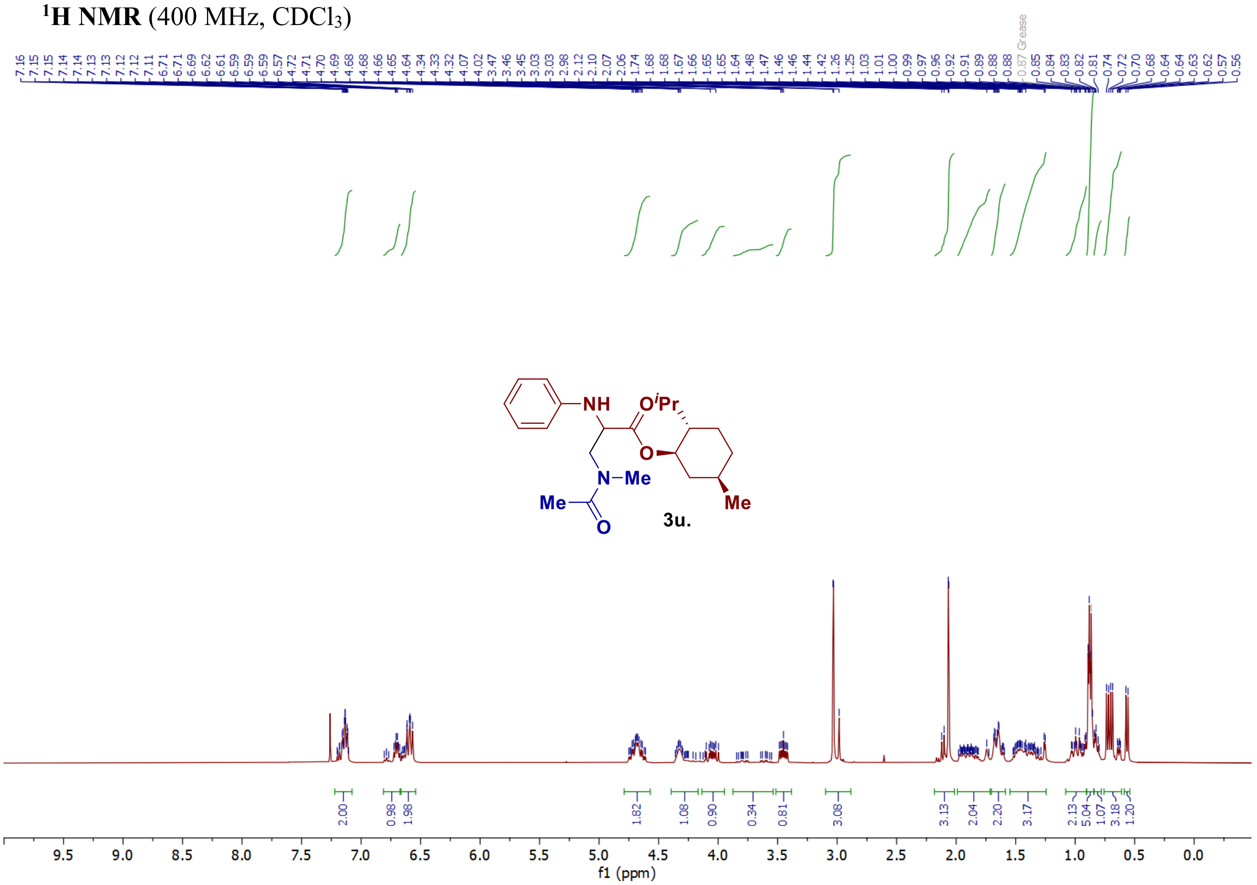

^{13}C NMR (101 MHz, $CDCl_3$)

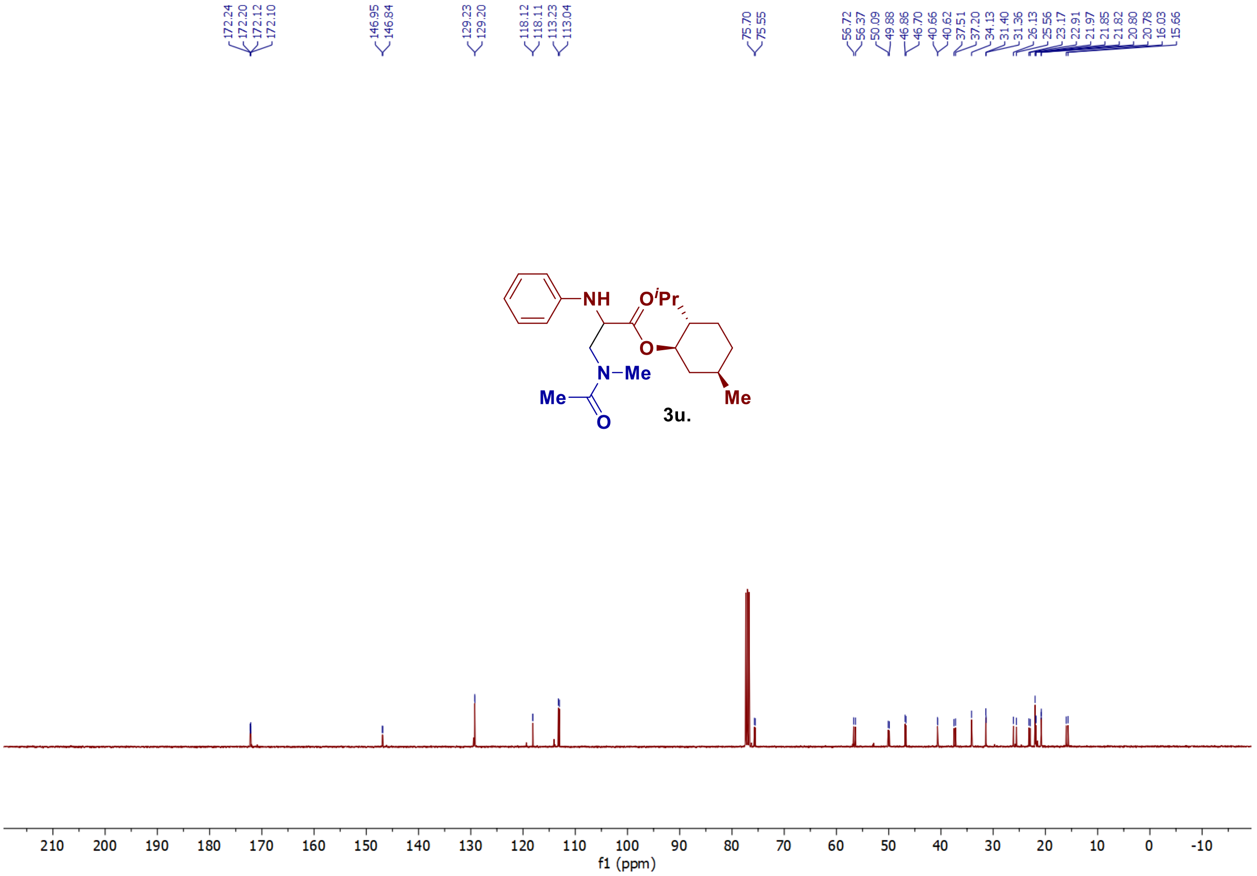

^{1}H NMR (400 MHz, $CDCl_3$)

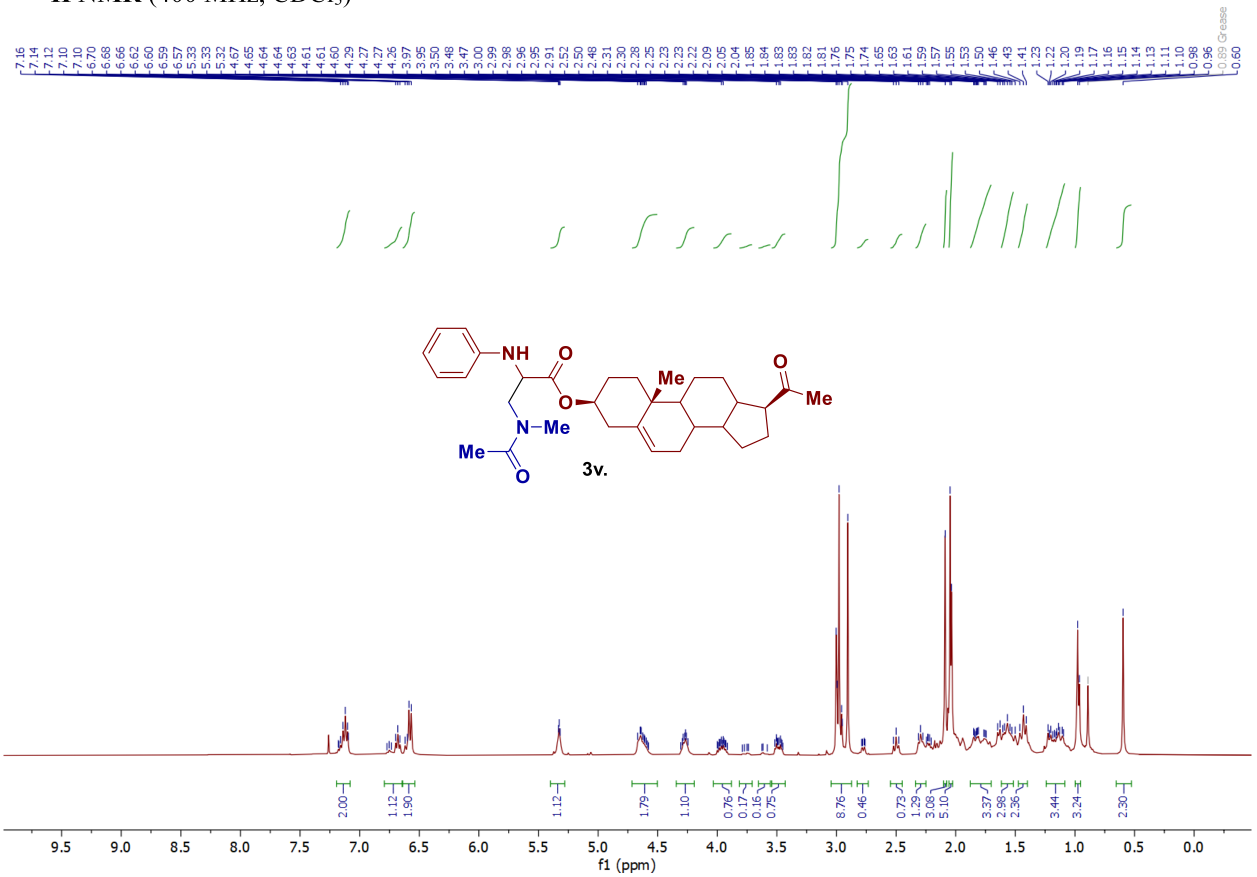

^{13}C NMR (101 MHz, $CDCl_3$)

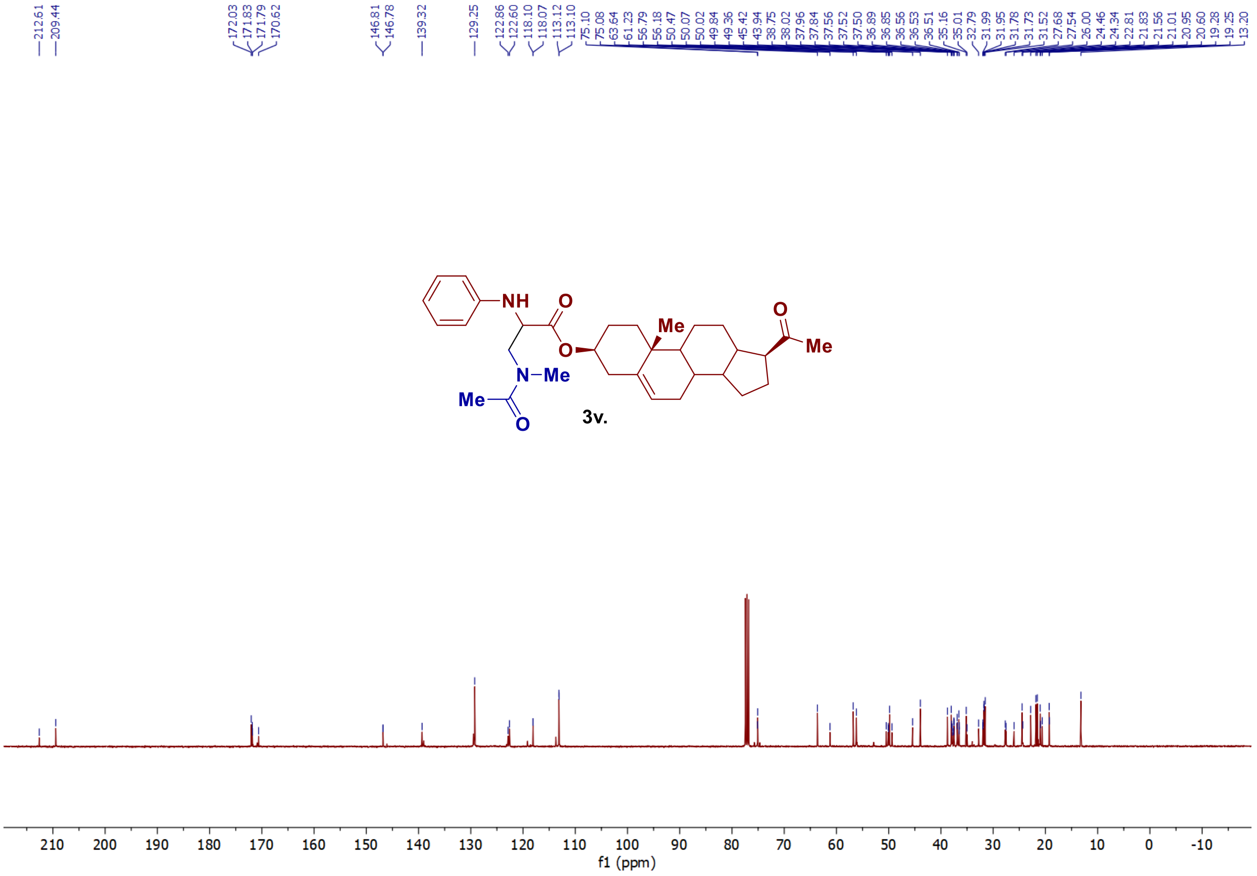

10. References.

17. K. Murugesan, K. Donabauer, R. Narobe, V. Derdau, A. Bauer, B. König, *ACS Catal.* **2022**, *12*, 3974– 3984.
18. B. Kalluraya, S. Aamir, A. Shabaraya, *Eur. J. Org. Chem*. **2012**, *54*, 597-604.
19. H. Zhi, S. P. M. Ung, Y. Liu, L. Zhao, C. J. Li, *Adv. Synth. Catal.* **2016**, *358*, 2553-2557.
